# Total Synthesis of (+)‐Erogorgiaene and the Pseudopterosin A−F Aglycone via Enantioselective Cobalt‐Catalyzed Hydrovinylation

**DOI:** 10.1002/chem.202101863

**Published:** 2021-06-26

**Authors:** Sohajl Movahhed, Julia Westphal, Alexander Kempa, Christian Eric Schumacher, Julia Sperlich, Jörg‐Martin Neudörfl, Nicole Teusch, Matthias Hochgürtel, Hans‐Günther Schmalz

**Affiliations:** ^1^ University of Cologne Greinstrasse 4 50939 Koeln Germany; ^2^ TH Köln, Faculty of Applied Natural Sciences Kaiser-Wilhelm-Allee, G. E39 51373 Leverkusen Germany

**Keywords:** cationic cyclization, cobalt catalysis, diterpenes, glycosides, hydrovinylation, marine natural products

## Abstract

Due to their pronounced bioactivity and limited availability from natural resources, metabolites of the soft coral *Pseudopterogorgia elisabethae*, such as erogorgiaene and the pseudopterosines, represent important target molecules for chemical synthesis. We have now developed a particularly short and efficient route towards these marine diterpenes exploiting an operationally convenient enantioselective cobalt‐catalyzed hydrovinylation as the chirogenic step. Other noteworthy C−C bond forming transformations include diastereoselective Lewis acid‐mediated cyclizations, a Suzuki coupling and a carbonyl ene reaction. Starting from 4‐methyl‐styrene the anti‐tubercular agent (+)‐erogorgiaene (>98 % ee) was prepared in only 7 steps with 46 % overall yield. In addition, the synthesis of the pseudopterosin A aglycone was achieved in 12 steps with 30 % overall yield and, surprisingly, was found to exhibit a similar anti‐inflammatory activity (inhibition of LPS‐induced NF‐κB activation) as a natural mixture of pseudopterosins A−D or *iso*‐pseudopterosin A, prepared by β‐*D*‐xylosylation of the synthetic aglycone.

## Graphical abstract

**Applied metal catalysis**: Exploiting a practical protocol for the asymmetric hydrovinylation of stryrenes in the opening move, the stereoselective total synthesis of bioactive constituents of the soft coral *pseudopterogorgia elisabethae* was efficiently achieved in sequence of metal‐mediated steps to provide sufficient material for further biological investigations.

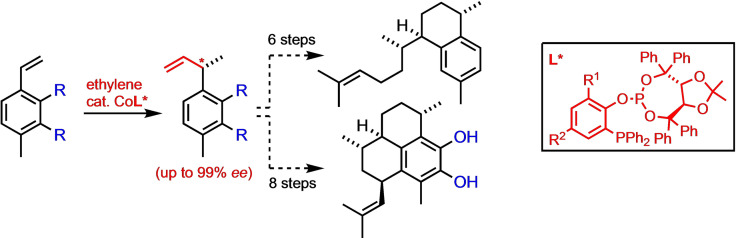

Marine organisms in general and the soft coral *Pseudptero‐gorgia elisabethae* in particular represent a rich source of bioactive natural products, some of which are of high pharmaceutical interest.^[1]^ Prominent examples are (+)‐erogorgiaene (**1**)^[2]^ and the pseudopterosins^[3]^ (e. g. **2**, **3** and **4**), the latter being biosynthetically derived from **1**.^[4]^ While **1** shows promising activity against *Mycobacterium tuberculosis* H_37_Rv,^[2]^ the pseudopterosins have received outstanding scientific and even commercial[^1a^, ^5^] attention due to their strong anti‐inflammatory and analgesic properties.[^3a^, ^3b^, ^3c^, ^3d^, ^6^] More recently, the unique bioactivity of the pseudopterosins was attributed to their ability to block the NFκB pathway through activation of the glucocorticoid receptor.^[7]^ Moreover, these compounds also reveal interesting cytotoxic and antibacterial properties,^[8]^ and pseudopterosin A (**3**) was identified as a potent broad‐spectrum antibiotic agent against different pathogenic strains^[9]^ The same compound (**3**) was also shown to protect synaptic function and to exhibit neuromodulatory properties while being well distributed within mammalian tissues.^[10]^

Due to their promising biological activity and the scarcity of material from natural sources, both erogorgiaene (**1**) and the pseudopterosins represent relevant target molecules for chemical synthesis. Actually, total synthesis currently appears to be the most sustainable way to provide substantial amounts of these compounds for possible pharmaceutical development. Accordingly, several research groups have elaborated synthetic routes towards erogorgiaene,^[11]^ the pseudopterosins^[12]^ and other structurally related marine diterpenes.^[13]^ However, a careful analysis of the reported syntheses reveals that most (if not all) of the known approaches do not reach the required levels of overall efficiency (number of steps, yield, and stereoselectivity). We here disclose a particular short and efficient access to both (+)‐erogorgiaene (**1**) and the pseudopterosin A−F aglycone following a general strategy which exploits an enantioselective Co‐catalyzed hydrovinylation[^14^, ^15^] as chirogenic step ‐ in combination with diastereoselective cationic cyclization steps.

Our strategy, sketched in Scheme 1, is based on the consideration that ring C of the tricyclic pseudopterosins can be formed by a late (“biomimetic”)^[4]^ cationic cyclization. Thus, all target molecules shown in Figure 1 can retrosynthetically be traced back to precursors of type **5**, which in turn should be accessible from calamenenes of type **6** through diastereoselective double bond functionalization. In a previous study we had already shown that compound **6** **b** can be obtained from **7** **b** by *trans*‐selective Lewis acid‐catalyzed cyclization under proton‐free conditions.^[16]^ However, our original method for the preparation of **7** **b** (by enantioselective hydroboration of **9** **b** followed by double Matteson homologation and Suzuki coupling) turned out to be little attractive from an operational point of view and difficult to be scaled up.^[16]^ Therefore, we sought to apply our operationally convenient protocol for the asymmetric cobalt‐catalyzed hydrovinylation^[14]^ to enantioselectively convert the vinyl‐arenes **9** **a** and **9** **b** into the chiral olefins **8** **a** and **8** **b**, respectively. Intermediates of type **7** should then be accessible in a single step by hydroboration of **8** and subsequent Suzuki cross‐coupling.^[17]^

**Scheme 1:**
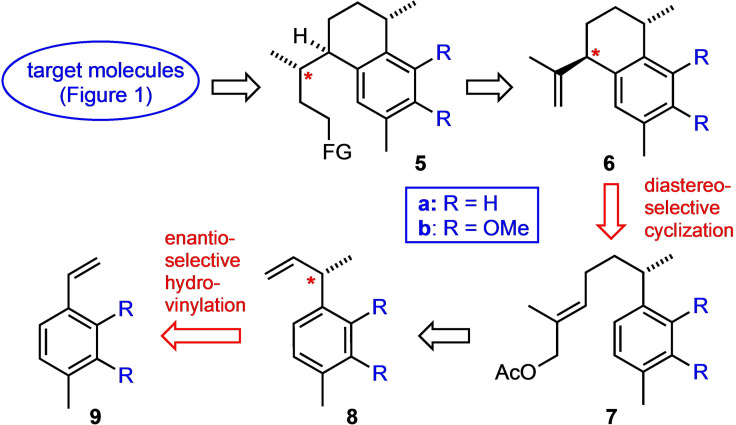
A general strategy towards marine diterpenoids related to erogorgiaene and the pseudopterosins.

**Figure 1:**
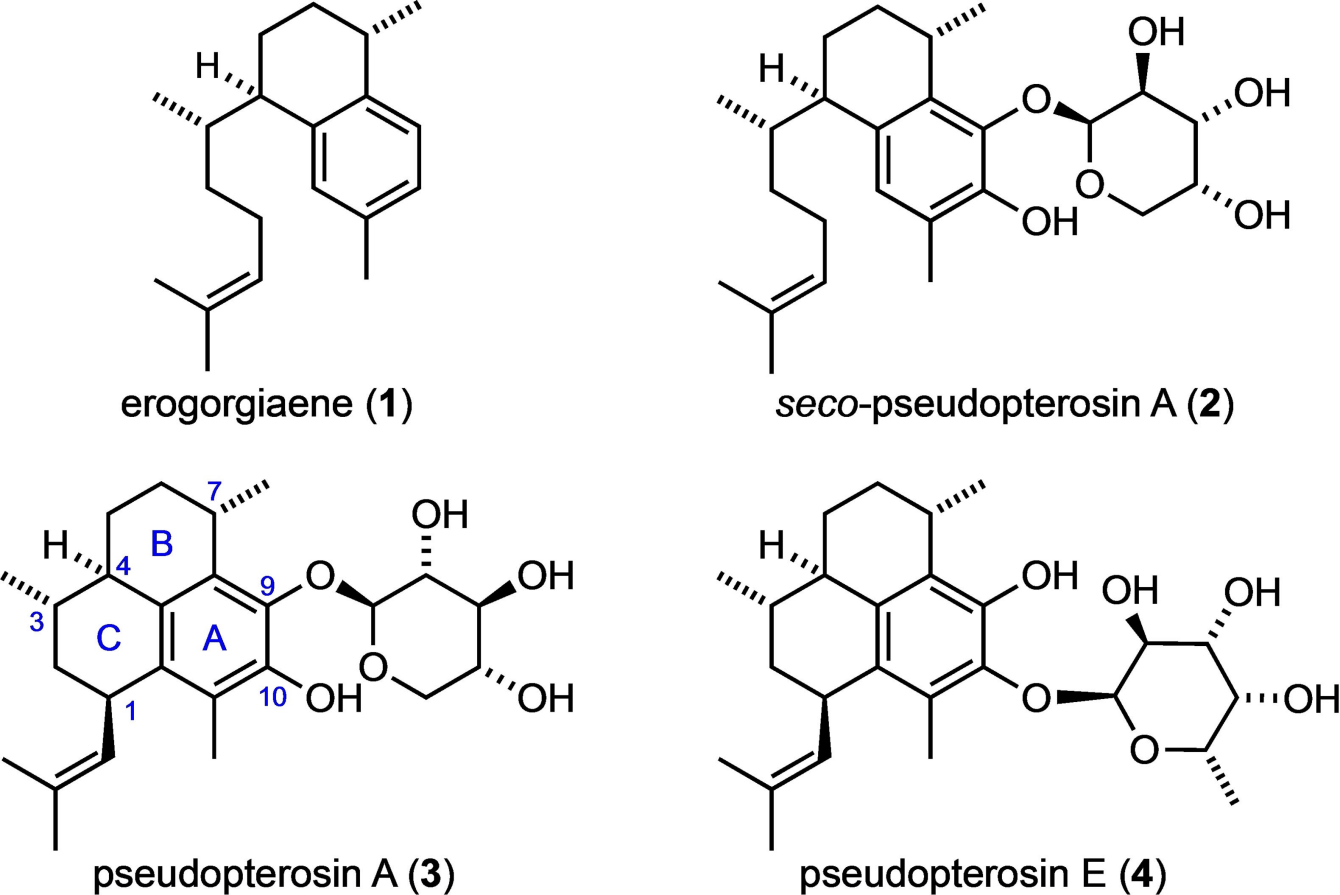
Structure of (+)‐erogorgiane (**1**) and selected members of the pseudopterosin family of marine diterpene gylcosides.

Following this plan, we first investigated the hydrovinylation of **9** **a** employing chiral ligands **L1** and **L2**, which had given the best results with other substrates in our previous study.^[14]^ In addition, we tested the more electron‐rich new ligand **L3**, which differs from **L1** by a methoxy group at the ligand backbone.^[18]^

The air‐stable pre‐catalysts (L*CoCl_2_) were prepared by stirring CoCl_2_ and the respective chiral ligand in THF for 16 h at room temperature. After solvent removal and re‐dissolution in dichloromethane the mixture was cooled to the specified temperature under an atmosphere of ethylene (1.2 bar) before substrate **9** **a** and Et_2_AlCl (as an activator) were injected. As Table 1 shows, the reactions proceeded smoothly at −40 °C within 2–3 h to give the (*R*)‐configurated product (*R*)‐**8** **a** in high yield (entries 1–3).^[19]^ While **L1** and **L2** behaved similarly (89 % *ee*), the methoxy‐substituted ligand **L3** ^[18]^ proved to be even more active in this case and selectively afforded **8** **a** with 93 % *ee*. By lowering the temperature to −60 °C the enantioselectivity could be further improved (entry 4). Under optimized conditions (using only 0.03 mol% of the *ent*‐**L3**‐based catalyst at −65 °C) the reaction could be reliably performed on a multi‐gram scale (5 g) to afford the desired (*S*)‐configurated product (*S*)‐**8** **a** in almost quantitative isolated yield and 98–99 % enantiomeric excess.^[20]^

**Table 1:** Behavior of different ligands and temperature effects in the cobalt‐catalyzed enantioselective hydrovinylation of 4‐methylstyrene (**9** **a**).

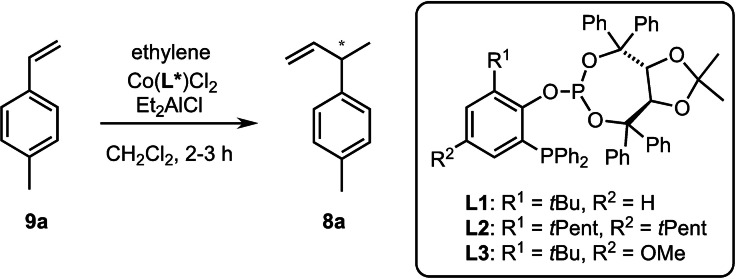					
Entry	L*	Temp. [°C]	**8** **a** ^[a]^ [%]	*ee*^[b]^ [%]	abs. config.^[c]^
1	**L1** (1 mol %)	−40	100	89	*R*
2	**L2** (1 mol %)	−40	97	89	*R*
3	**L3** (1 mol %)	−40	100	93	*R*
4	**L3** (1 mol %)	−60	100	97	*R*
5	**L3** (1 mol %)	−78	0	–	–
6	*ent‐***L3** (0.03 mol %)	−65	98^[d]^	98‐99^[e]^	*S*

Reaction conditions: **9** **a** (1.0 eq), CH_2_Cl_2_, ethylene (1.2 bar), Co(L*)Cl_2_, Et_2_AlCl (Co : Al=1 : 6). [a] GC yield of **8** **a** as determined by FID‐GCMS; [b] Determined by FID‐GC on a chiral stationary phase; [c] Determined by comparison of the optical rotatory values with those given in Ref. [19]. [d] Isolated yield (gram scale); [e] The reaction was performed several times to reproducibly afford (*S*)‐**8** **a** with 98–99 % *ee*.

According to the chosen strategy, the next task was the elongation of the side chain to convert (*S*)‐**8** **a** into the allylic acetate **7** **a** (Scheme 2). For this purpose, (*S*)‐**8** **a** was first hydroborated with 9‐BBN and the in situ formed intermediate **10** **a** was directly coupled under Suzuki conditions with the vinyl iodide **12** to give **7** **a** in high yield. Building block **12** was prepared from propargylic alcohol (**11**) through Zr‐catalyzed methyl alumination/iodination^[21]^ followed by acetylation of the alcohol function according to a previously described protocol.[^13h^, ^17^]

**Scheme 2:**
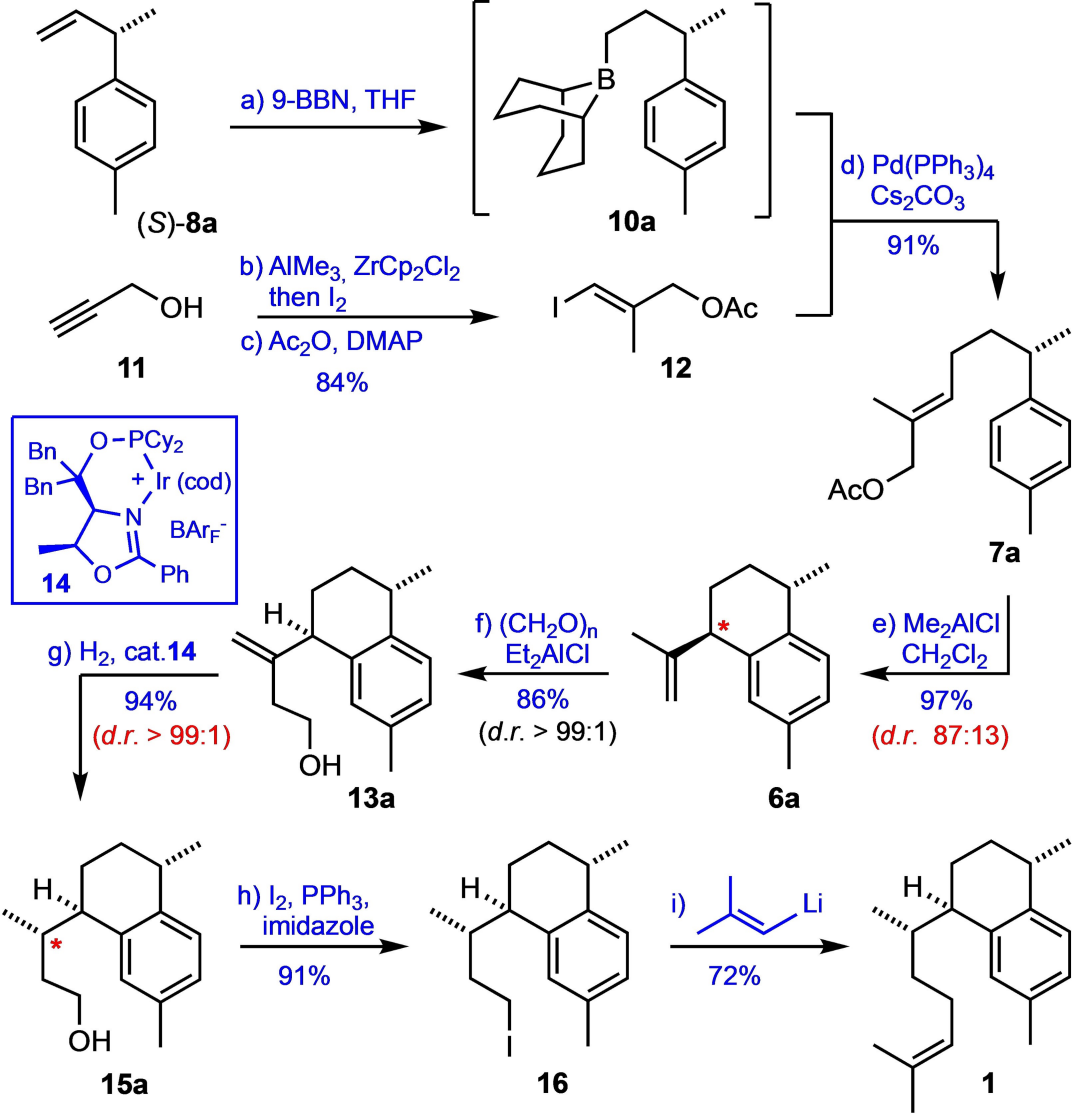
Total synthesis of (+)‐erogorgiaene (**1**). Reagents and conditions: (a) 9‐BBN, THF, r.t., 6 h; (b) Me_3_Al, Cp_2_ZrCl_2_ (25 mol%), CH_2_Cl_2_, then I_2_, THF, 0 °C→rt; (c) NEt_3_, cat. DMAP, Ac_2_O, CH_2_Cl_2_, 0 °C→r.t.; (d) addition of **10** **a** in THF to a suspension of **12**, Cs_2_CO_3_ and Pd(PPh_3_)_4_ (5 mol%) in DMF, H_2_O, 40 °C, 18 h; (e) Me_2_AlCl, CH_2_Cl_2_, −78 °C, 6 h; (f) (CH_2_O)_n_, Et_2_AlCl, CH_2_Cl_2_, −70 °C, 1.5 h; separation of diastereomers by preparative HPLC; (g) *Pfaltz* catalyst **14** (2 mol%), H_2_ (35 bar), CH_2_Cl_2_, r.t., 2 d; separation of diastereomers by column chromatography; (h) I_2_, PPh_3_, imidazole, CH_2_Cl_2_, r.t., 30 min; (i) isocrotyl‐lithium, THF, −78 °C→r.t., 18 h. 9‐BBN=9‐borabicyclo[3.3.1]‐nonane; DMAP=4‐*N,N*‐dimethylaminopyridin.

After screening several reaction conditions (see Table SI‐1) we found that the Lewis acid‐mediated cyclization of nuciferyl acetate (**7** **a**) was best performed by treating a solution of **7** **a** in dichloromethane at −78 °C with Me_2_AlCl. This way, the *trans*‐calamenene **6** **a** was obtained in high yield and good diastereoselectivity (87 : 13 *d.r*.). After elongation of the side chain by means of a carbonyl Alder‐ene reaction (using paraformaldehyde in the presence of Et_2_AlCl)^[22]^ the diastereomers could be separated by preparative HPLC to give the stereochemically pure alcohol **13** **a** in 86 % isolated yield. Introduction of the side chain stereocenter was then achieved by diastereoselective hydrogenation of **13** **a** using the Ir‐catalyst **14** developed by Pfaltz and coworkers.^[23]^ The crude product (containing 4 % of the undesired diastereomer) could be readily purified through flash chromatography to afford pure **15** **a** in 94 % yield. With this compound in hand, the synthesis of the target molecule **1** was then efficiently concluded in only two steps, i. e. by iodination of the alcohol function (I_2_, PPh_3_, imidazole) and coupling of the resulting iodide **16** with isocrotyl‐lithium.[^11a^, ^24^] This way, (+)‐erogorgiaene (**1**) was obtained in 46 % overall yield over seven steps starting from commercial 4‐methylstyrene (**9** **a**). At this point, the configurational assignments were confirmed by careful comparison of the NMR data with those reported by Aggarwal, who had prepared all four diastereomers of **1**.^[11d]^ In addition, the optical rotation of our synthetic sample ([α]_D_=+22°; c=0.18) matched the reported value for the natural product.^[2a]^

Following the same general strategy (Scheme 1), we next tackled the synthesis of the pseudopterosin A−F aglycone (**20**) as a second target molecule in this study (Scheme 3). Starting from the styrene derivative **9** **b**, which is available from veratrole in three steps,^[16]^ the first task was to achieve the Co‐catalyzed hydrovinylation to **8** **b**.^[25]^ According to our previous experience, this proved to be more challenging (as compared to the hydrovinylation of **8** **a**) due to the additional methoxy‐substituent in *ortho*‐position to the vinyl group.[^14^, ^26^] However, after careful optimization of the conditions (Table SI‐2) the desired transformation could be successfully performed on a gram scale (2.7 g) using a **L2**‐derived catalyst to afford the alkene **8** **b** (84 % *ee*) in 87 % isolated yield (after distillation). Noteworthy, ligand **L2**, prepared from (*R*,*R*)‐Taddol afforded the desired (*S*)‐configurated product **8** **b**, while the same ligand gave rise to the (*R*)‐configurated product when substrate **9** **a** was employed (see Table 1).^[27]^

**Scheme 3:**
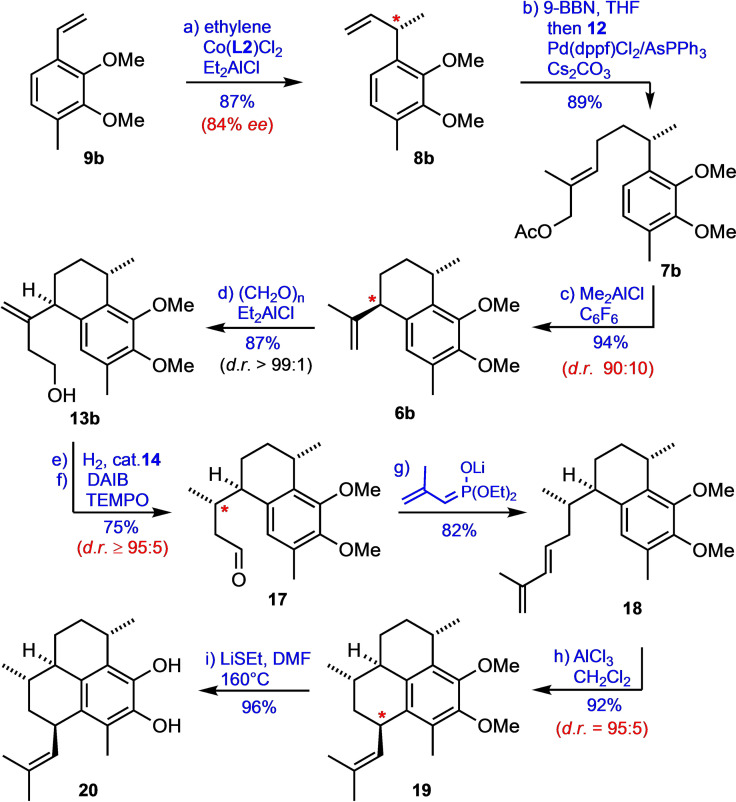
Total synthesis of the pseudopterosin A−F agylcone (**20**). Reagents and conditions: (a) ethylene (1.2 bar), Co(**L2**)Cl_2_ (5 mol%), Et_2_AlCl (30 mol%), CH_2_Cl_2_, −20 °C, 6 h; (b) 9‐BBN, THF, r.t., 24 h; then transfer to a suspension of **12**, [Pd(dppf)Cl_2_] • CH_2_Cl_2_ (5 mol%), AsPh_3_ (5 mol%) and Cs_2_CO_3_ in H_2_O/DMF, then μW (50 W), 40 °C, 2 h; (c) Me_2_AlCl, C_6_F_6_, 5 °C, 1.5 h; (d) (CH_2_O)_n_, Et_2_AlCl, CH_2_Cl_2_, 20 °C, sonification, 4.5 h; separation of diastereomers by column chromatography; (e) **14** (1 mol%), H_2_ (20 bar), CH_2_Cl_2_, r.t., 48 h; (f) DAIB, TEMPO (20 mol%), CH_2_Cl_2_, r.t., 1 h, separation of diastereomers by column chromatography; (g) diethyl (2‐methylallyl)phosphonate, *n*‐BuLi, TPPA, THF, −78 °C to r.t., 6 h.; (h) AlCl_3_ (20 mol%), CH_2_Cl_2_, −5 °C, 4.5 h; (i) LiSEt (10 equiv), DMF, 160 °C, 3 h. dppf=1,1’‐bis(diphenylphosphino)ferrocene; TEMPO=2,2,6,6‐tetramethylpiperidin‐1‐yl‐oxyl; DAIB=(diacetoxy)iodobenzene; TPPA=tripyrrolidinophosphoric acid triamide.

The conversion of **8** **b** into the allylic acetate **7** **b** was achieved in 89 % yield by hydroboration (9‐BBN) and microwave‐assisted Suzuki coupling of the resulting borane intermediate with the vinyl iodide **12**. The following Friedel‐Crafts‐type cyclization of **7** **b** (under strictly aprotic conditions to avoid the undesired disproportionation of the product)^[16]^ reproducibly afforded **6** **b** with high yield and *trans*‐diastereoselectivity (*d.r*.=9 : 1) using Me_2_AlCl as a Lewis acid in hexafluorobenzene as a solvent. As in the synthesis of **1** (Scheme 2) the diastereomers were not separable at this stage. However, after elongation of the side chain by Et_2_AlCl‐mediated carbonyl‐ene reaction with paraformaldehyde under sonification, the isomers could be separated by flash column chromatography, and the diastereomerically pure alcohol **13** **b** was isolated in 87 % yield. Hydrogenation of the double bond, again using the Ir‐catalyst **14**, proceeded highly diastereoselectively (*d.r*.=94 : 6).^[28]^ After TEMPO‐mediated oxidation of the alcohol with diacetoxyiodobenzene^[29]^ the aldehyde **17** was obtained in 75 % yield over two steps. To complete the serrulatane skeleton, **17** was transformed with diethyl (2‐methylallyl)phosphonate^[30]^ in a Horner‐Wadsworth‐Emmons olefination to afford the (*E*)‐configurated diene **18**.

As the final critical step of the synthesis of the pseudopterosin A−F aglycone (**20**) we carefully investigated the diastereoselective cationic cyclization of **18** to the amphilectane **19**. In this context we first tested various acids and Lewis acids using CH_2_Cl_2_ as a solvent (see Table S3). While most of these reagents only gave low conversions and/or selectivities, the by far best result was obtained with anhydrous aluminum chloride. Under optimized conditions the cyclization of **18** proceeded efficiently in the presence of 20 mol% of AlCl_3_ in CH_2_Cl_2_ at −5 °C to afford the desired product **19** in 92 % isolated yield and with high diastereoselectivity (*d.r*.=95 : 5).^[31]^ The relative and absolute configuration of the cyclization product **19** was unambiguously proven by X‐ray analysis of a single crystal obtained from a melt (Figure 2).^[32]^ The concluding cleavage of both methoxy groups then proceeded smoothly upon heating of **19** with an excess of LiSEt in DMF[^12f^, ^33^] to afford the target molecule **20** in high yield (40 % overall from **8** **b** over 8 steps).

**Figure 2:**
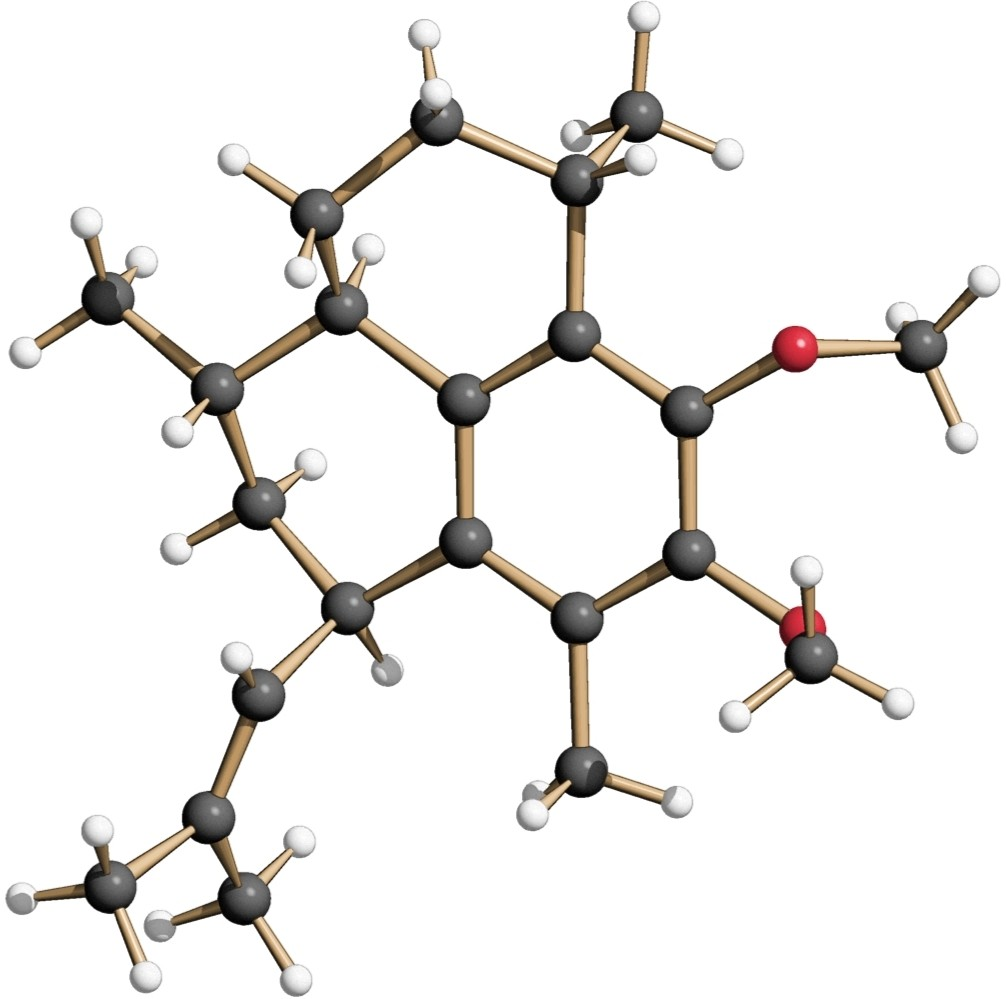
Structure of **19** in the crystalline state.

While the conversion of the aglycone **20** into pseudopterosins A (**3**) and E (**4**), respectively, has been previously reported,[^12a^, ^12b^] we synthesized the new glycosidic compound *is*o‐pseudopterosin A (*iso‐* **3**) in a 6 : 1 mixture with **3** by treatment of **20** (freshly prepared from the more stable and storable precursor **19**) with the *D*‐xylose‐derived α‐trichloroacetimidate **21** in the presence of BF_3_ etherate under strictly anhydrous conditions (Scheme 4).^[34]^

**Scheme 4:**
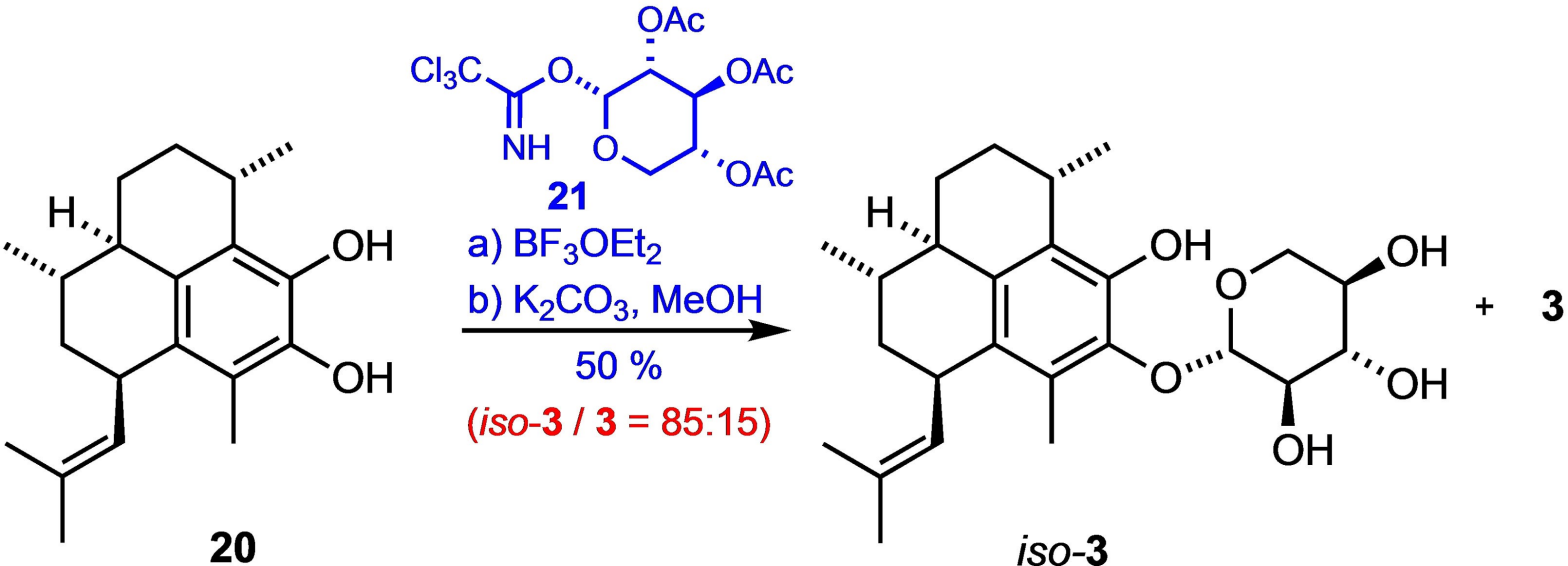
Synthesis of *iso*‐pseudopterosin A (*iso*‐**3**): Reagents and conditions: a) **21**, BF_3_^.^Et_2_O, 4 Å MS, CH_2_Cl_2_, −78 °C, 30 min; b) K_2_CO_3_, MeOH, r.t., 18 h.

The resulting mixture of β‐xylosides (*iso*‐**3**/**3**=85 : 15) was finally assessed with respect to its ability to inhibit the NFκB pathway^[7]^ in comparison with compounds **19** and **20** as well as a mixture of pseudopterosins A−D from natural sources as a control. We found that the synthetic *iso*‐**3**/**3** mixture is at least as active as the mixture of pseudopterosins A−D (Figure 3). Interestingly, the aglycone **20** proved to be equally potent as the corresponding glycosides in this assay,^[7]^ while the dimethyl ether **19** was nearly inactive.

**Figure 3:**
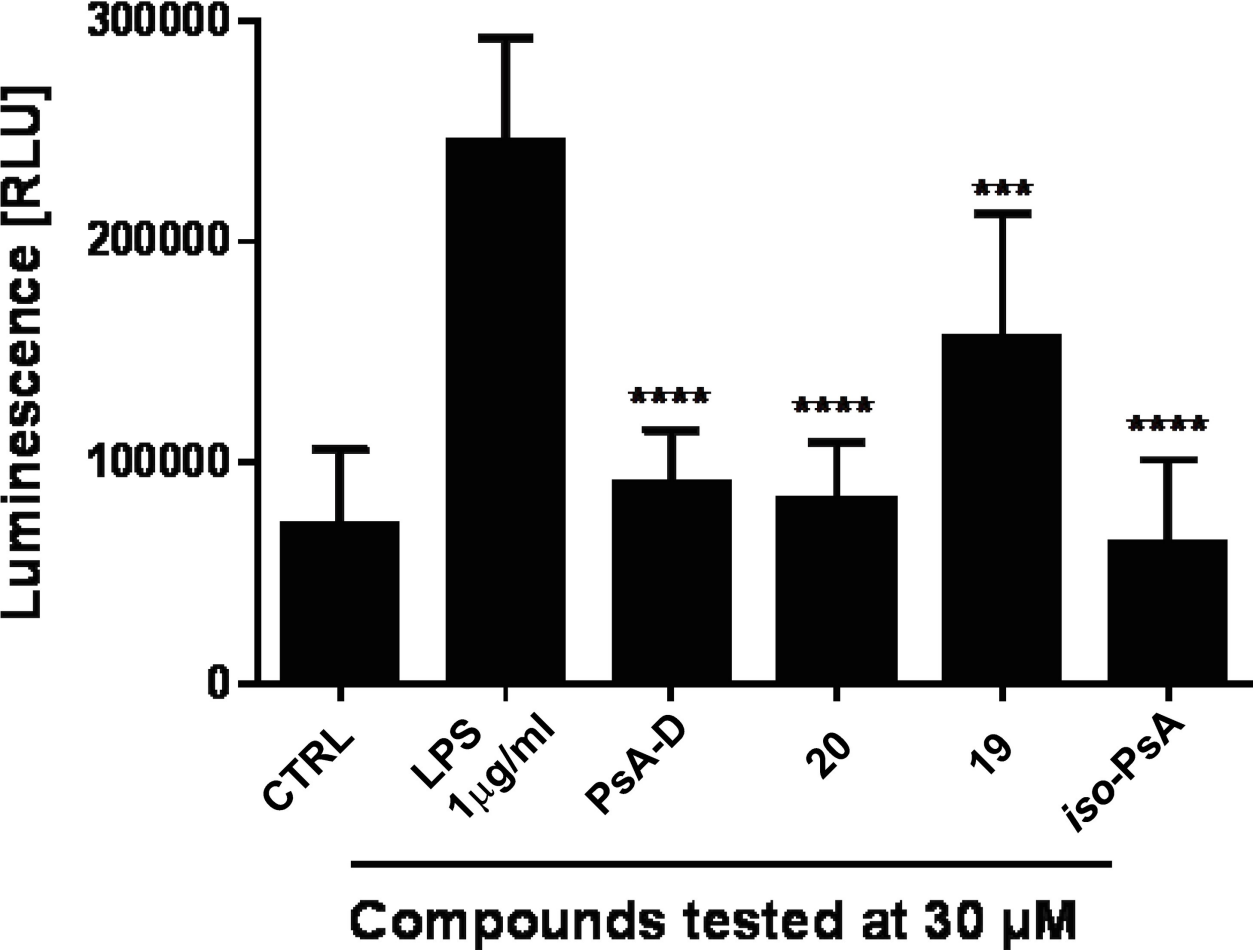
Anti‐inflammatory activity of natural pseudopterosins A−D (mixture) in comparison to the synthetic compounds **19**, **20** (pseudopterosin A−F aglycone), and *iso*‐pseudopterosin A (*iso*‐**3**/**3**=6 : 1) as reflected by the inhibition of the NFκB pathway in LPS‐stimulated MDA‐MB‐231 breast cancer cells.

This result somewhat contradicts earlier reports on structure‐activity relationships of the pseudopterosins and derivatives, which had indicated the importance of the sugar unit for the anti‐inflammatory activity in mouse ear tests.[^3a^, ^3b^, ^3c^, ^3d^, ^6^] This indicates that a major function of the glycoside moiety may be to protect the sensitive catechol moiety of the aglycone from oxidation, besides improving the water solubility of the natural product.

In summary, we have developed a powerful, general strategy for the stereoselective total synthesis of the marine natural products erogorgiaene and the pseudopterosins. In the chirogenic opening step, we exploited a Co‐catalyzed enantioselective hydrovinylation, thus demonstrating the practicality of this methodology in the context of total synthesis. The synthetic sequences, mainly based on metal‐catalyzed or ‐mediated transformations, also feature highly selective cationic cyclizations and the diastereoselective elaboration of the serrulatane side chain by substrate‐controlled Pfaltz hydrogenation of the carbonyl‐ene products **15** **a** and **15** **b**, respectively. Both target molecules were obtained in less than 10 steps with high overall yield. The efficient access to the pseudopterosin aglycone (**20**) enabled us to also prepare *iso*‐pseudopterosin A, a novel anti‐inflammatory compound, which proved to be equally active as a mixture of natural pseudopterosins in the inhibition of the NFκB pathway. Thus, we are optimistic that this work will stimulate research into the pharmaceutical exploitation of pseudopterosins and related natural products in the future ‐ without the need to harvest the corals.

## Conflict of interest

The authors declare no conflict of interest.

## Supporting information

As a service to our authors and readers, this journal provides supporting information supplied by the authors. Such materials are peer reviewed and may be re‐organized for online delivery, but are not copy‐edited or typeset. Technical support issues arising from supporting information (other than missing files) should be addressed to the authors.

Supporting Information
