# Supplementary material for: Total Synthesis of (+)‐Erogorgiaene and the Pseudopterosin A−F Aglycone via Enantioselective Cobalt‐Catalyzed Hydrovinylation
Source: Chemistry. 2021 Jun 26;27(45):11574–9. doi: 10.1002/chem.202101863 (PMC8456859; doi:10.1002/chem.202101863)
Supplement: Supplementary file 1 — Supporting Information [file CHEM-27-11574-s001.pdf]

# Chemistry–A European Journal

Supporting Information


## Total Synthesis of (+)-Erogorgiaene and the Pseudopterosin A–F Aglycone via Enantioselective Cobalt-Catalyzed Hydrovinylation


Sohajl Movahhed[+], Julia Westphal[+], Alexander Kempa, Christian Eric Schumacher, Julia Sperlich, Jörg-Martin Neudörfl, Nicole Teusch, Matthias Hochgürtel, and Hans-Günther Schmalz*

# Content

1. General Experimental ........ 2
2. Screening for reaction conditions ........ 4
3. Synthetic procedures and substance data ........ 7
   3.1. Synthesis of the chiral phosphine-phosphite ligand L3 ........ 7
   3.2. Synthesis of the styrene derivative 9b ........ 10
   3.3. Synthesis of (+)-erogorgiaene (1) ........ 13
   3.4. Synthesis of the pseudopterosin A-F aglycone (20) ........ 23
   3.5. Synthesis of iso-pseudopterosin A (*iso*-3) ........ 35
4. $^{1}$H and $^{13}$C NMR Spectra ........ 41
5. X-Ray Crystal Data ........ 63
6. Enantiomeric analysis through chiral GC ........ 64
7. Determination of anti-inflammatory activities ........ 67

# 1. General Experimental

All oxygen- or moisture-sensitive reactions were carried out under an argon-atmosphere using Schlenk technique. Glassware was flame-dried under vacuum and allowed to cool down under an argon atmosphere. Solids were added in continuous flow of argon. Syringes and cannula were flushed with argon prior to use. Oxygen or water sensitive substances were stored and handled in a *Braun* Labmaster 130 glovebox. Tetrahydrofuran (THF) and diethylether were freshly distilled under an argon atmosphere from sodium/benzophenone. Dichloromethane, tetrachloromethane and hexafluoro-benzene were distilled from calcium hydride under argon. All other reagents were obtained from *ABCR, Acros*, *Sigma Aldrich*, *Alfa Aesar* or *Rockwood Lithium* and used without further purification, unless otherwise indicated.

**Hydrogenations** were carried out using a 50 ml *Parr Instrument Company* 5500 pressure reactor.

**Column chromatography** as well as flash chromatography was performed using *Silica gel for chromatography,* (0.035-0.070 mm, 60 Å) from *Acros*. Analytical thin-layer chromatography (TLC) was performed with commercial aluminum plates coated with Silica Gel 60-F254 from *Merck*. Chromatograms were visualized by UV light at 254 nm, or by staining with a “$KMnO_4$ reagent” (prepared from 1.5 g of $KMnO_4$, 10 g of $K_2CO_3$ and 1.25 ml 10% NaOH in 200 ml of $H_2O$) or a cerium molybdenum solution (2 g phosphomolybdic acid, 1 g $Ce(SO_4)_2$, 10 ml $H_2SO_4$ in 90 ml $H_2O$) and subsequent heating.

**Nuclear magnetic resonance** (NMR) spectra ($^1H$, $^{13}C$, $^{31}P$) were recorded in $CDCl_3$ or $CD_3OD$ on *Bruker* instruments Avance II 300 or DPX 300 (both 300 MHz), Avance HD400N (400 MHz), Avance II 500 (500 MHz) or Avance II 600 (600 MHz). Chemical shifts (δ) are reported in in parts per million (ppm) relative to tetramethylsilane or the corresponding solvent signal. The fine structure of proton signals is given as s (singlet), d (doublet), t (triplet), q (quartet), p (quintet), h (hextet) or m (multiplet) or br (broad). In the case of $^{13}C$ NMR, either standard spectra or APT (attached proton test) spectra were recorded. $^{13}C$ NMR spectra were recorded proton decoupled. To unambiguously assign the signals, H,H-COSY, HMQC and HMBC spectra were recorded. The atom numbering for assignment is specified in each case and does not necessarily follow the IUPAC nomenclature. If possible, spin systems of higher order are described phenomenologically as pseudo (Ψ) fine structures.

**Infrared spectra** (IR) were recorded on a *Perkin–Elmer* Paragon 100 FT-IR spectrometer or a *Bruker* Platinum both using the ATR technique. The intensity of absorption bands is given as s (strong), m (medium) or w (weak); b additionally indicates broad signals.

**Mass spectra** (MS) and high resolution mass spectra (HRMS) were measured with a MAT Incos 50 Galaxy System (EI) from *Finnigan* with an ionization potential of 70 eV or a Thermo LTQ Orbitrap XL from *Thermo Fisher Scientific* (ESI). Gas-chromatography with mass selective detection (GC–MS) was

carried out using an *Agilent* GC6890 instrument using a HP 5973 detector on 30 m x 0.25 mm capillary columns (Optima-1-MS from *Macherey-Nagel*) with $H_2$ as a carrier gas (1.7 ml/min, 1.2 bar). As a routine, the following temperature program was used: 50 °C (2 min), then heating to 300 °C (12 min), finally 300 °C (5 min). Signals are referred to m/z ratio; the intensities are specified relatively to the height of the highest peak (100%).

**Enantiomeric analyses** by chiral GC were performed on an *Agilent* HP 6890 instrument using a MEGA-DEX BSE or a BGB 176 SE (both 30 m x 0.25mm) as capillary columns and $H_2$ as carrier gas.

**Optical rotation** was measured at a *Anton Paar* MCP200 polarimeter, a *Perkin-Elmer* 343 or a *IBZ Messtechnik* POLAR LμP polarimeter, at 20 °C and with defined wavelength. Concentration is given in g/100 ml of solvent.

**X-Ray** measurements were carried out using a *Bruker* D8 Venture four circle diffractometer.

## 2. Screening for reaction conditions

**Table SI-1**: Screening of *Lewis* acids (top) and solvents (bottom) for the diastereoselective *Lewis* acid-mediated ring closure of **7a**.

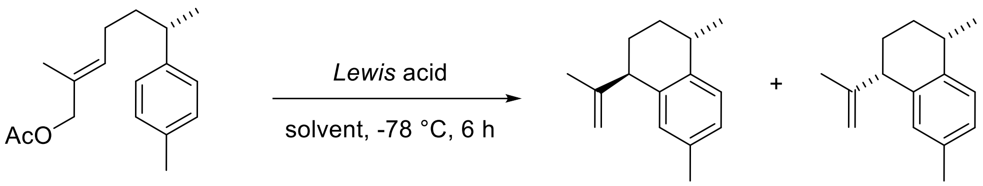


**7a** *trans*-**6a** *cis*-**6a**

| entry | *Lewis* acid | conversion [%][a] | 6a [%][a] | *trans/cis*[b] |
|---|---|---|---|---|
| **1** | **$Me_2AlCl$** | **100** | **100** | **87: 13** |
| **2** | $Et_2AlCl$ | 100 | 100 | 62 : 38 |
| **3** | $Et_3Al_2Cl_3$ | 77 | 16 | 62 : 38 |
| **4** | *i*$Bu_3Al$ | 100 | 45 | 66 : 35 |
| **5** | $Me_3Al$ | 100 | 2 | n. d. |
| **6** | $AlCl_3$ | 100 | 26 | 86 : 14 |
| **7** | TMSOTf | 100 | 74 | 84 : 16 |
| **8** | $FeCl_3$ | 13 | 0 | - |

Reaction conditions: Allylacetate **7a** (1.0 eq), time of addition of the Lewis acid (2.5 eq): 30 min; $CH_2Cl_2$ as solvent, 6 h, -78 °C [a] Determined by FID-GCMS. [b] Determined by FID-GC on a chiral stationary phase.

| entry | solvent | temperature | conversion [%][a] | 6a [%][a] | *trans/cis*[b] |
|---|---|---|---|---|---|
| **1** | toluene | -78 °C | 100 | 100 | 80 : 20 |
| **2** | *n*-heptane | -78 °C | 29 | 4 | 79 : 21 |
| **3** | *n*-pentane | -78 °C | 0 | 0 | - |
| **4** | cyclohexane | -78 °C | 0 | 0 | - |
| **5** | THF | -78 °C | 0 | 0 | - |
| **6** | Acetonitrile | -40 °C | 16 | 13 | 65 : 35 |
| **7** | **$CH_2Cl_2$** | **-78 °C** | **100** | **100** | **87 : 13** |
| **8** | $CHCl_3$ | -60 °C | 100 | 81 | 74 : 26 |
| **9** | $CCl_4$ | 0 °C | 100 | 100 | 67 : 33 |
| **10** | $C_2H_4Cl_2$ | -30 °C | 100 | 100 | 80 : 20 |
| **11** | $C_6F_6$ | 0 °C | 100 | 100 | 68 : 32 |

Reaction conditions: Allylacetate **7a** (1.0 eq), time of addition of $Me_2AlCl$ as *Lewis* acid (2.5 eq): 30 min, solvent, 6 h, -78 °C [a] Determined by FID-GCMS. [b] Determined by FID-GC on a chiral stationary phase.

**Table SI-2**: Screening of several phosphine-phosphite ligands in the hydrovinylation of vinylarene **9b**.

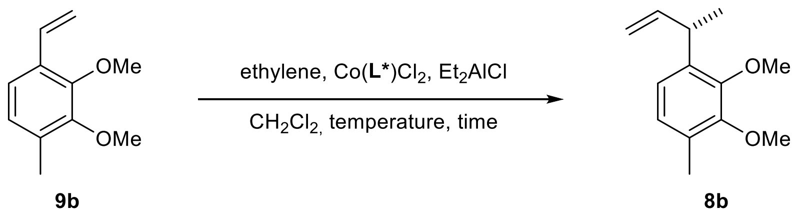


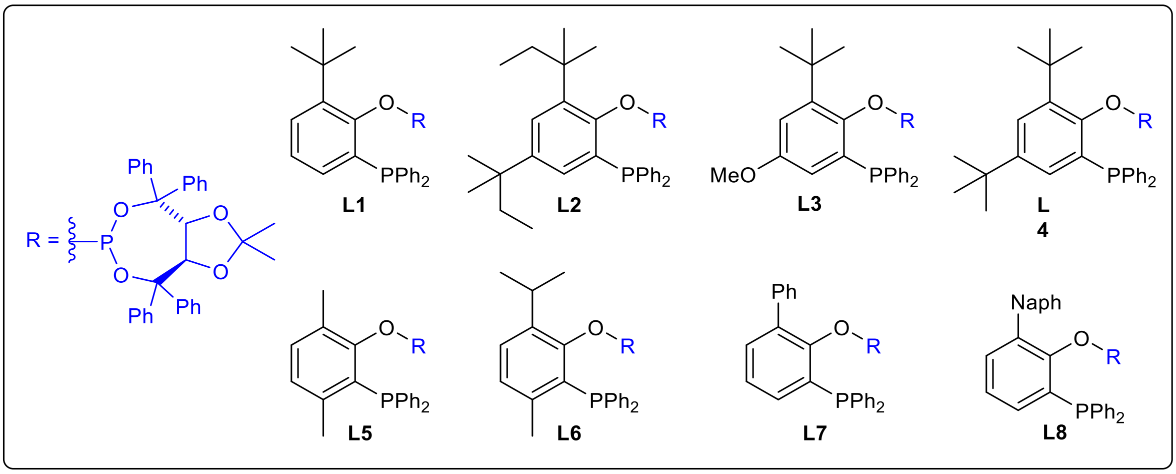


| entry | ligand | temp. [°C] | time [h] | conv. [%][a] | conv. to 8b [%][a] | *ee* [%][b,c] |
|---|---|---|---|---|---|---|
| **1** | **L5** | 20 | 4 | 0 | - | - |
| **2** | **L6** | 20 | 4 | 0 | - | - |
| **3** | **L7** | 0 | 6 | 26 | 26 | 15 |
| **4** | **L8** | -15 | 8 | 52 | 52 | 60 |
| **5** | **L1** | 0 | 8 | 23 | 23 | 60 |
| **6** | **L3** | -15 | 8 | 52 | 52 | 70 |
| **7** | **L4** | -15 | 24 | 83 | 77 | 80 |
| **8** | **L2** | -15 | 3 | 81 | 81[d] | 81 |
| **9** | **L2** | -20 | 6 | 83 | 87[d] | 84 |

Conditions: Substrate **9b** (1 equiv, 0.2 M in $CH_2Cl_2$), ethylene (1.2 bar), Co(**L***)$Cl_2$ (5 mol%), $Et_2AlCl$ (30 mol%). [a] Determined by GC-FID. [b] Determined by GC-FID on a chiral stationary phase. [c] Ligand screenings with equal substitution patterns at the phenol backbone utilizing Tartrol or BINOL instead of Taddol as chiral unit led to significantly lower enantiomeric excesses at comparable conversions. [d] Isolated yield.

**Table SI-3:** Cationic cyclization to tricyclic compound **19**.

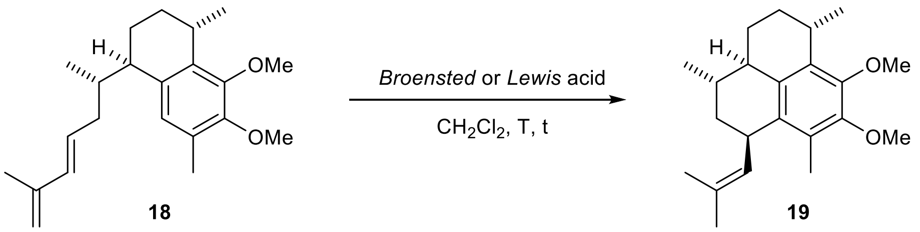


| entry | ***Brønsted* acid** | **Temperature, time** | **Conversion [%][a]** | ***d.r.* [%][a]** |
|---|---|---|---|---|
| **1** | $H_3CSO_3H$ (1.0 eq) | -78 °C → 0 °C, 3 h | 61 | 60 :40 |
| **2** | $F_3CSO_3H$ (1.0 eq) | -78 °C, 4 h | 90 | 42 : 58 |
| **3** | Nafion-H D20 | rt, 4 h | 0 | - |

Reaction conditions: Diene **18** (1.0 eq), 0.1 M in $CH_2Cl_2$, acid, T, t. [a] Determined by FID-GC.

| entry | ***Lewis* acid (40 mol%)** | **Conversion [%][a]** | ***d.r.* [%][a]** |
|---|---|---|---|
| **1** | Li(OTf) | 15 | 76:24 |
| **2** | $Sn(OTf)_2$ | 73 | 44:56 |
| **3** | $Al(OTf)_3$ | 55 | 42:58 |
| **4** | $Fe(OTf)_3$ | 73 | 41:59 |
| **5** | $Y(OTf)_3$ | 24 | 48:52 |
| **6** | $Sm(OTf)_3$ | 28 | 50:50 |
| **7** | $Eu(OTf)_3$ | 29 | 52:48 |
| **8** | $Yb(OTf)_3$ | 22 | 57:43 |
| **9** | $Me_2AlCl$ (2.0 eq) | 0 | - |
| **10** | **$AlCl_3$** | **100** | **93:7** |
| **11** | $FeCl_3$ | 100 | 85:15 |
| **12** | $InCl_3$ | 73 | 88:12 |
| **13** | $BF_3{\cdot}Et_2O$ (1.0 eq) | 92 | 89:11 (-78 to 0°C) |

Reaction conditions: Diene **18** (1.0 eq), 6 mM in $CH_2Cl_2$, *Lewis* acid (0.4 eq), 0 °C, 5 h. [a] Determined by FID-GC.

# 3. Synthetic procedures and substance data

## 3.1. Synthesis of the chiral phosphine-phosphite ligand L3

### 3.1.1. 2-Bromo-6-(*tert*-butyl)-4-methoxyphenol (S1)

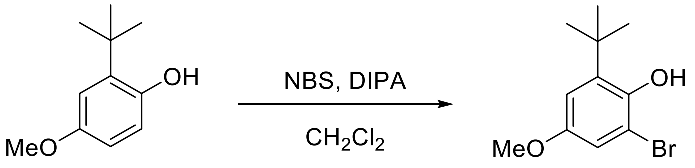


**S2** **68 %** **S1**

A round-bottom flask was equipped with a Soxhlet extractor and a reflux condenser with a drying tube. The Soxhlet extractor was fitted with an extraction thimble which was filled with 5.43 g (30.0 mmol, 1.1 eq) of *N*-bromosuccinimide. The flask was charged with phenol **S2** (5.00 g, 33.4 mmol, 1.0 eq), which was dissolved in 75 ml of abs. $CH_2Cl_2$. Then 0.39 ml (2.80 mmol, 0.1 eq) of di*iso*propylamine was added. The solution was then heated to reflux for 20 h. After cooling to r.t., the mixture was treated with 60 ml of 2 M $H_2SO_4$, the layers were separated and the aqueous layer was extracted three times with MTBE. The combined organic layers were washed with brine, dried over $MgSO_4$ and filtered. Removal of solvent under reduced pressure afforded the crude product as a yellow oil which was purified by column chromatography (CyHex/EtOAc 100:1). The desired bromide **S1** could be isolated in a yield of 4.90 g (18.9 mmol, 68 %) as colourless oil.

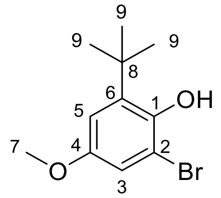


| | |
|---|---|
| **M** ($C_{11}H_{15}BrO_2$) | 259.14 g/mol. |
| **$R_f$** | 0.4 (CyHex/EtOAc 100:1). |
| **$^1$H NMR** | (300 MHz, $CDCl_3$): δ [ppm] = 6.86 (d, *J* = 3.0 Hz, 1 H, H5), 6.84 (d, *J* = 3.0 Hz, 1 H, H3), 5.41 (s, 1 H, O*H*), 3.74 (s, 3 H, H7), 1.38 (s, 9 H, H9). |
| **$^{13}$C NMR** | (75 MHz, $CDCl_3$): δ [ppm] = 152.9 (C4), 144.8 (C1), 138.4 (C6), 114.4 (C2), 113.1 (C3), 111.5 (C5), 55.9 (C7), 35.5 (C8), 29.2 (C9). |
| **FT-IR** (ATR) | ν [$cm^{-1}$] = 3511 (br), 2999 (m), 2953 (m), 2908 (s), 2870 (m), 1570 (s), 1470 (s), 1419 (vs), 1362 (m), 1292 (s), 1216 (vs); 1165 (s), 1053 (vs), 923 (br), 874 (m), 770 (s), 736 (m), 624 (w). |
| **GC-MS** (70 eV) | m/z (%) = 258 (45, $[M]^+$), 243 (80, $[M-CH_3]^+$), 215 (50, $[M-C_2H_4]^+$), 180 (45), 165 (100, $[M-CH_3]^+$), 150 (20, $[M-CH_3]^+$), 137 (90), 121 (15), 107 (20), 91 (50, $[C_7H_7]^+$), 77 (50, $[C_6H_5]^+$), 53 (30). |

### 3.1.2. 2-(Diphenylphosphinyl)-4-methoxy-6-*tert*-butylphenol Borane Complex (S3)

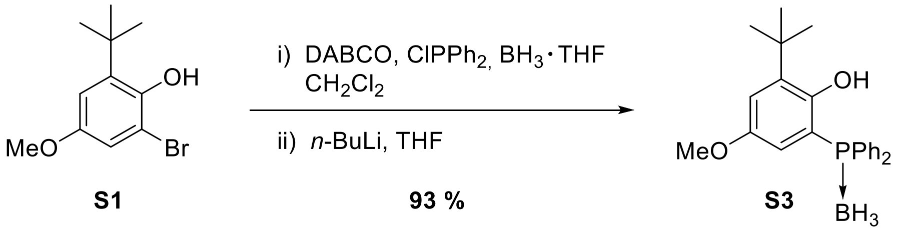


A Schlenk flask was charged with 2.30 g (8.93 mmol, 1.0 eq) of bromide **S1** and 1.20 g (10.7 mmol, 1.2 eq) of DABCO in 5 ml of abs. $CH_2Cl_2$. The resulting solution was stirred for 15 min at rt and then cooled to 0 °C. Then 2.00 ml (10.7 mmol, 1.2 eq) of $ClPPh_2$ were added dropwise by using a syringe pump over a period of 30 min. The resulting white suspension was stirred for 30 min at this temperature, then allowed to warm to rt and stirred for 2 h. The reaction mixture was cooled to 0 °C again and 17.9 ml (17.9 mmol, 2.0 eq) of $BH_3$ • THF (1.0 M in THF) were added. The suspension was stirred for 10 min at 0 °C, then allowed to warm to rt and stirred for 1 h. The reaction mixture was quenched with 10 ml of deionized $H_2O$, the layers were separated and the aqueous layer was extracted three times with MTBE. The combined organic layers were washed with brine, dried over $MgSO_4$, filtered and concentrated under reduced pressure. The crude product was dried and then dissolved without further purification in 20 ml of abs. THF under argon atmosphere. The solution was cooled to 0 °C and 6.80 ml (17.9 mmol, 2.0 eq) of *n*-BuLi (2.6 M in hexanes) were added using a syringe pump over 30 min. The resulting red solution was stirred for 2 h at 0 °C and then quenched with 60 ml of deionized $H_2O$. The layers were separated and the aqueous layer was extracted three times with MTBE. The combined organic layers were washed with sat. aq $NH_4Cl$, dried over $MgSO_4$ and the solvent was removed under reduced pressure. The crude product was purified by column chromatography (CyHex/EtOAc 100:1) to yield 3.14 g (8.31 mmol, 93 %) of borane-protected phosphine **S3** as a colourless oil.

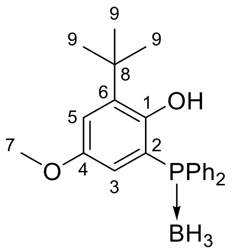


| | |
|---|---|
| **M** ($C_{23}H_{28}BO_2P$) | 378.25 g/mol. |
| **$R_f$** | 0.3 (CyHex/EtOAc 50:1). |
| **$^1$H NMR** | (300 MHz, $CDCl_3$): δ [ppm] = 7.59-7.43 (m, 10 H, $H_{Ar}$), 7.21 (s, 1 H, O*H*), 7.05 (d, *J* = 3.0 Hz, 1 H, H5), 6.18 (dd, *J* = 11.3, 3.0 Hz, 1 H, H3), 3.54 (s, 3 H, H7), 1.39 (s, 9 H, H9). |
| **$^{13}$C NMR** | (75 MHz, $CDCl_3$): δ [ppm] = 153.6 (d, *J* = 8.9 Hz, C1), 152.5 (d, *J* = 10.3 Hz, C4), 140.6 (d, *J* = 6.3 Hz, C6), 133.0 (d, *J* = 9.8 Hz, $C_{Ar}$), 131.6 ($C_{Ar}$), 128.9 (d, *J* = 10.5 Hz, $C_{Ar}$), 128.1 (d, *J* = 61.3 Hz, $C_q$), 118.0 (C5), 115.0 (C3), 112.80 (d, *J* = 58.5 Hz, C2), 55.4 (C7), 35.4 (C8), 29.4 (C9). |
| **$^{31}$P NMR** {1H} | (121 MHz, $CDCl_3$): δ [ppm] = 14.59 (d, *J* = 57.3 Hz, $PR_3$). |

**FT-IR** (ATR) ν [$cm^{-1}$] = (3372 (br), 2957 (br), 2375 (br), 1597 (m), 1436 (s), 1363 (m), 1296 (m), 1221 (vs), 1107 (s), 1058 (s), 999 (w), 740 (vs), 693 (vs).

**GC-MS** (70 eV) m/z (%) = 380 (20, $[M]^+$), 364 (100, $[M\text{-}BH_3]^+$), 347 (60), 322 (20), 207 (10), 183 (10), 162 (10), 133 (10), 109 (10), 91 (20, $[C_7H_7]^+$), 61 (10).

### 3.1.3. (3a*R*,8a*R*)-6-(2-(*tert*-Butyl)-6-(diphenylphosphanyl)-4-methoxyphenoxy)-2,2-dimethyl-4,4,8,8-tetraphenyltetrahydro-[1,3]dioxolo[4,5-*e*][1,3,2]dioxaphosphepine (L3)

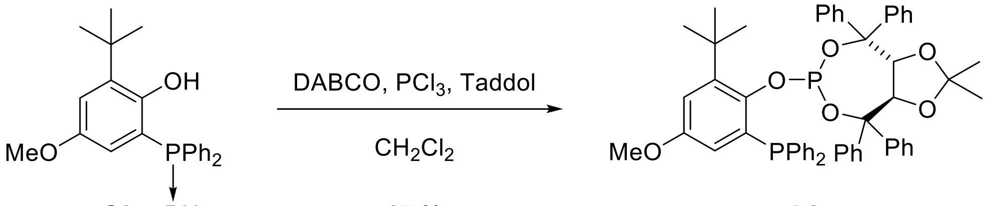


**S3** **67 %** **L3**

Under argon atmosphere 1.19 g (3.15 mmol, 1.0 eq) of borane-protected phosphine **S3** and 2.84 g (25.2 mmol, 8.0 eq) of DABCO were dissolved in 25 ml of abs. $CH_2Cl_2$. The resulting solution was stirred for 10 min at rt, then cooled to 0 °C before 1.88 ml (3.78 mmol, 1.2 eq) of a 2.0 M solution of $PCl_3$ in abs. $CH_2Cl_2$ were added dropwise by using a syringe pump over 30 min. The resulting slurry was stirred for 30 min at this temperature, then allowed to warm to rt and stirred for 3 h. The reaction mixture was cooled to 0 °C and a solution of (*S*,*S*)-TADDOL (2.21 g, 4.75 mmol, 1.5 eq) in 25 ml of abs.$CH_2Cl_2$ was added dropwise by using a syringe pump over 30 min. The resulting suspension was stirred for 30 min at 0 °C, then allowed to come to rt and stirred for 16 h at rt. The reaction mixture was filtered over silica gel and the filtrate was concentrated under reduced pressure. The resulting crude product was purified by column chromatography on ultra pure silica gel (CyHex/$CH_2Cl_2$ 4:1) to yield 1.90 g (2.10 mmol, 67 %) of chiral phosphine-phosphite ligand **L3** as colorless foam.

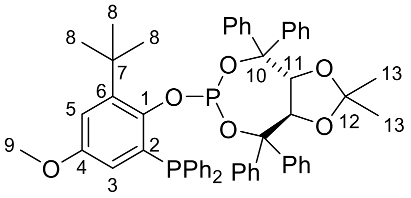


**M** ($C_{54}H_{52}O_6P_2$) 858.93 g/mol.

**$R_f$** 0.3 (CyHex/$CH_2Cl_2$ 4:1).

**mp.** 117-120°C (decopmposition).

**$^1$H NMR** (300 MHz, $CDCl_3$): δ [ppm] = 7.56-7.40 (m, 10 H, $H_{Ar}$), 7.23-7.05 (m, 20 H, $H_{Ar}$), 6.91 (d, *J* = 3.0 Hz, 1 H, H5), 6.40 (d, *J* = 3.0 Hz, 1 H, H3), 5.35 (d, *J* = 8.1 Hz, 1 H, H11), 5.17 (d, *J* = 7.2 Hz, 1 H, H11), 3.51 (s, 3 H, H9), 1.36 (s, 9 H, H8), 0.85 (s, 3 H, H13), 0.45 (s, 3 H, H13).

**$^{13}$C NMR** (75 MHz, $CDCl_3$): δ [ppm] = 154.2 (C4), 150.1 (C1) 145.3, 142.5 (C6), 141.7, 141.1, 138.3, 137.7 (C2), 133.3, 133.2, 133.1, 133.0, 129.1, 128.9, 128.2, 128.0, 127.9, 127.8, 127.6, 127.5, 127.4, 127.3, 127.1, 127.0, 126.9, 126.8,

| | |
|---|---|
| | 117.1 (C7), 116.4 (C5), 113.2 (C12), 86.5 (C10), 83.0 (C10), 81.7 (C11), 81.0 (C11), 55.2 (C9), 35.5 (C7), 30.5 (C8), 26.9 (C13), 26.1 (C13). |
| **$^{31}$P-NMR** {1H} | (121 MHz, $CDCl_3$): δ [ppm] = 139.8 (d, $J$ = 197.0 Hz, $P(OR)_3$), -18.5 (d, $J$ = 150.1 Hz, $PR_3$). |
| **FT-IR** (ATR) | ν [$cm^{-1}$] = 3057 (w), 2989 (s), 2957 (br), 2871 (s), 2832 (s), 2378 (w), 1586 (w), 1447 (m), 1434 (m), 1407 (s), 1202 (m), 1183 (m), 1050 (s), 1033 (s), 1012 (s), 884 (s), 824 (s), 738 (s), 696 (vs). |

### 3.1.4 In situ preparation of the $CoCl_2$-L3 catalyst

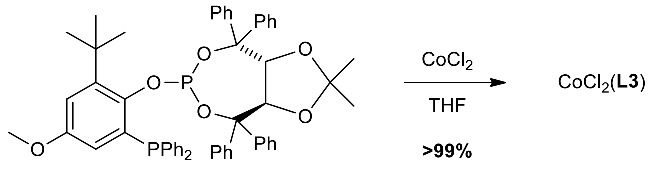


15.2 mg (0.117 mmol, 1.0 eq) of $CoCl_2$ were dissolved in 6 ml of THF under inert conditions. 100 mg (0.116 mmol, 1.0 eq) of ligand **L3**, dissolved in 6 ml of THF, were added to the $CoCl_2$ solution and the resulting mixture stirred for 18 h at ambient temperature. The solvent was removed under reduced pressure and the dark blue residue dried in vacuo to afford $CoCl_2$(**L3**) in a quantitative fashion. The catalyst was used without further purification.

## 3.2. Synthesis of the styrene derivative 9b

### 3.2.1. 1,2-Dimethoxy-3-methylbenzene (S4)[1]

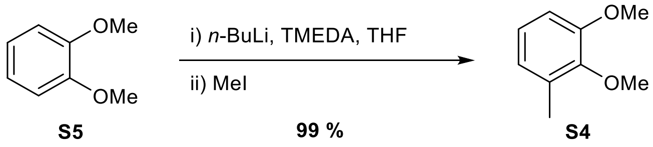


11.0 ml (86.3 mmol, 1.0 eq) of veratrole (**S5**) and 18.0 ml (121.0 mmol, 1.4 eq) of TMEDA were dissolved in 200 ml of anhydrous THF under inert atmosphere. The solution was cooled to 0 °C before 65.0 ml of *n*-BuLi (1.60 M in hexane, 104.0 mmol, 1.2 eq) were added dropwise over 2 h. Stirring was continued for 3 h at rt before the solution was cooled to -78 °C and 6.4 ml (104.0 mmol, 1.2 eq) of iodomethane were added dropwise over 1 h. The stirred solution was allowed to warm to rt over 15 h before a saturated solution of $NH_4Cl$ was added at 0 °C until the reaction mixture cleared off. After warming to rt additional 70 ml of $NH_4Cl$ solution were added and the layers were separated. The aqueous layer was extracted with 3 x 70 ml of MTBE and the combined organic layers were dried over $MgSO_4$. After filtration, the solvent was removed under reduced pressure and the crude product was purified by flash column chromatography (CyHex/EtOAc 20:1) to yield 13.0 g (85.4 mmol, 99%) of 1,2-dimethoxy-3-methylbenzene (**S4**) as a colorless oil.

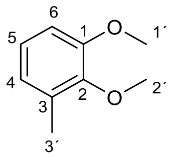


| | |
|---|---|
| **M** ($C_9H_{12}O_2$) | 152.19 g/mol. |
| **R$_f$** | 0.47 (CyHex/EtOAc 10:1). |
| **$^1$H NMR** | (300 MHz, $CDCl_3$): δ [ppm] = 6.94 (t, *J* = 7.9 Hz, 1 H, H5), 6.75 (d, *J* = 6.7 Hz, 2 H, H4/H6), 3.83 (s, 3 H, H1´), 3.79 (s, 3 H, H2´), 2.27 (s, 3 H, H3´). |
| **$^{13}$C NMR** | (75 MHz, $CDCl_3$): δ [ppm] = 152.8 (C1), 147.4 (C2), 132.1 (C3), 123.8 (C5), 122.9 (C4), 110.0 (C6), 60.1 (C2´), 55.7 (C1´), 15.8 (C3´). |
| **FT-IR** (ATR) | ν [cm$^{-1}$]: 2997 (w), 2937 (w), 2836 (w), 1587 (m), 1485 (s), 1474 (s), 1425 (m), 1304 (m), 1268 (s), 1223 (s), 1175 (m), 1094 (s), 1081 (s), 1009 (s), 910 (w), 806 (m), 772 (s), 745 (s), 686 (m). |
| **GC-MS** (70 eV) | m/z (%) = 152 (100, $[M]^+$), 137 (49), 109 (44), 94 (24), 77 (31), 51 (15). |

The analytical data are in agreement with the literature.[1]

### 3.2.2. 2,3-Dimethoxy-4-methylbenzaldehyde (S6)[1–3]

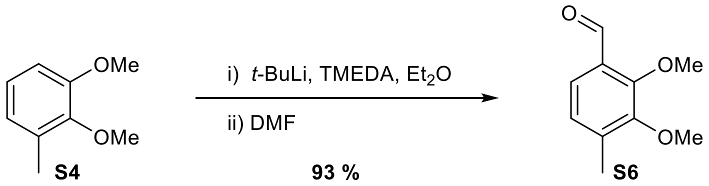


16.54 g (108.7 mmol, 1.0 eq) of 1,2-dimethoxy-3-methylbenzene (**S4**) and 30 ml (152.3 mmol, 1.4 eq) of TMEDA were dissolved in 600 ml of anhydrous diethyl ether under inert atmosphere. The colorless solution was cooled to 0 °C before 82 ml of *t*-BuLi (1.60 M in hexane, 130.8 mmol, 1.2 eq) were added dropwise over 1.5 h. Stirring was continued for 16 h at rt before the solution was cooled again to 0 °C and 17 ml (217.4 mmol, 2.0 eq) of DMF were added dropwise over 1 h. The stirred solution was allowed to warm to rt over 1 h before 600 ml of ice water and 20 ml of HCl (2 N) were added. The layers were separated, the aqueous layer was extracted with 3 x 200 ml of MTBE and the combined organic layers were dried over $MgSO_4$. After filtration, the solvent was removed under reduced pressure and the crude product was purified by flash column chromatography (CyHex/EtOAc 20:1) to yield 18.21 g (101.1 mmol, 93%) of 2,3-dimethoxy-4-methylbenzaldehyde (**S6**) as a colorless oil.

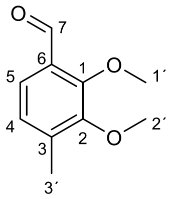


| | |
|---|---|
| **M** ($C_{10}H_{12}O_3$) | 180.20 g/mol. |
| **R$_f$** | 0.38 (CyHex/EtOAc 10:1). |

**¹H NMR** (300 MHz, $CDCl_3$): δ [ppm] = 10.34 (s, 1 H, H7), 7.49 (d, *J* = 8.0 Hz, 1 H, H5), 7.01 (d, *J* = 8.0 Hz, 1 H, H4), 4.00 (s, 3 H H1´), 3.86 (s, 3 H, H2´), 2.33 (s, 3 H, H3´).

**¹³C NMR** (75 MHz, $CDCl_3$): δ [ppm] = 189.4 (C7), 156.2 (C1), 151.5 (C2), 140.4 (C3), 128.5 (C6), 122.7 (C5), 126.1 (C4), 62.0 (C1´), 60.1 (C2´), 16.5 (C3´).

**FT-IR** (ATR) ν [$cm^{-1}$]: 2939 (w), 2856 (w), 2744 (w), 1686 (s), 1596 (m), 1574 (w), 1485 (w), 1463 (m), 1409 (m), 1383 (m), 1265 (s), 1251 (s), 1228 (m), 1181 (w), 1161 (w), 1069 (s), 1021 (s), 996 (m), 933 (w), 906 (m), 815 (m), 772 (s), 704 (w), 622 (m).

**GC-MS** (70 eV) m/z (%) = 180 (100, $[M]^+$), 165 (32), 150 (20), 134 (20), 119 (42), 91 (63), 65 (62), 50 (35).

The analytical data are in agreement with the literature.[1–3]

### 3.2.3. 2,3-Dimethoxy-1-methyl-4-vinylbenzene (9b)[3]

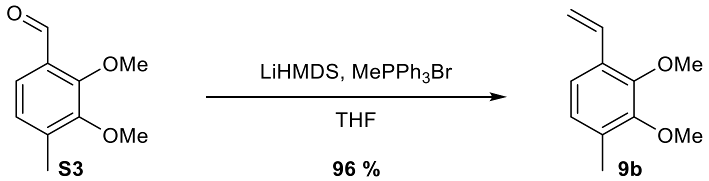


S3 96 % 9b

49.8 ml (1.3 M in THF, 64.7 mmol, 1.3 eq) of LiHMDS were diluted with 200 ml of anhydrous THF before 21.34 g (59.8 mmol, 1.2 eq) of $MePPh_3Br$ were added in portions and stirred for 1 h at rt. After this 8.97 g (49.8 mmol, 1.0 eq) of aldehyde **S3** in 150 ml of anhydrous THF were added dropwise over 1 h. The reaction mixture was refluxed for 24 h. After cooling to rt the mixture was diluted with 200 ml of pentane, filtered through a pad of celite and the solvent removed under reduced pressure. The crude product was purified by flash column chromatography (CyHex/EtOAc 20:1) to yield 8.52 g (47.8 mmol, 96%) of 2,3-dimethoxy-1-methyl-4-vinylbenzene (**9b**) as a colorless oil.

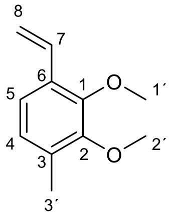


**M** ($C_{11}H_{14}O_2$) 178.23 g/mol.

**$R_f$** 0.58 (CyHex/EtOAc 20:1).

**¹H NMR** (300 MHz, $CDCl_3$): δ [ppm] = 7.14 (d, *J* = 8.0 Hz, 1 H, H5), 6.97 (dd, *J* = 17.8, *J* = 11.1 Hz, 1 H, H7), 6.85 (d, *J* = 8.0 Hz, 1 H, H4), 5.69 (dd, *J* = 17.8, 1.4 Hz, 1 H, $H8_{cis}$), 5.22 (dd, *J* = 11.1, 1.4 Hz, 1 H, $H8_{trans}$), 3.81 (s, 6 H, H1´, H2´), 2.24 (s, 3 H, H3´).

**¹³C NMR** (75 MHz, $CDCl_3$): δ [ppm] = 151.7 (C2), 150.8 (C1), 131.9 (C3), 131.3 (C7), 130.0 (C6), 125.8 (C4), 120.7 (C5), 114.1 (C8), 60.8 (C1´or C2´), 60.1 (C1´or C2´), 15.9 (C3´).

**FT-IR** (ATR) ν [$cm^{-1}$]: 3088 (w), 2989 (w), 2933 (w), 2862 (w), 2826 (w), 1626 (w), 1602 (w), 1568 (w), 1488 (m), 1459 (s), 1399 (s), 1271 (s), 1224 (s), 1177 (w), 1108 (w), 1071 (s), 1023 (s), 996 (s), 902 (s), 819 (s), 788 (m), 726 (w), 703 (w), 666 (w), 610 (w).

**GC-MS** (70 eV) m/z (%) = 178 (100, $[M]^+$), 163 (86), 149 (18), 135 (89), 120 (15), 105 (44), 91 (100), 77 (32), 63 (29).

**HRMS** (ESI) m/z: 201.0890 u (calc.: m/z = $[M+Na]^+$: 201.0886 u).

The analytical data are in agreement with the literature.[1,3]

## 3.3. Synthesis of (+)-erogorgiaene (1)

### 3.3.1 (*S*)-4-Methyl-1-(but-1-ene-3-yl)benzene (8a)

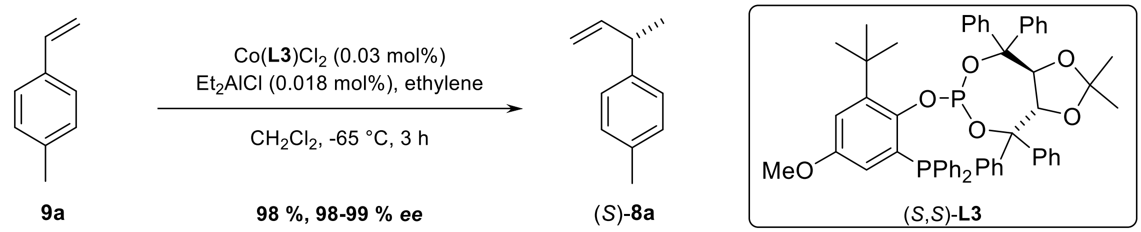


**9a** **98 %, 98-99 % *ee*** (*S*)-**8a** (*S*,*S*)-**L3**

A dried Schlenk flask was charged with the preformed Co((*S*,*S*)-**L3**)$Cl_2$ complex (11.9 mg, 0.012 mmol, 0.03 mol%) under inert atmosphere, then evacuated and flooded with ethylene (1.1 to 1.2 bar). After injecting 150 ml of anhydrous dichloromethane, the solution was cooled to -65 °C and 0.072 ml (0.072 mmol, 0.18 mol%) $Et_2AlCl$ (1.0 M in hexanes) followed by 4-methylstyrene (**9a**) (5.28 ml, 40.1 mmol, 1.0 eq) were added. After 3 h at -65 °C, the reaction mixture was exposed to air and subsequently filtered through a large column charged with a 10-15 cm layer of silica gel with *n*-pentane (ca. 1 l) to remove all catalyst components and some polyethylene (formed as a by-product). The solvent was removed under slightly reduced pressure (caution: the product is rather volatile) to yield 5.75 g (39.4 mmol, 98 %) of the desired hydrovinylation product (**8a**) as a colorless oil (98-99 % *ee* according to chiral GC analysis). Analytical enantiomeric separation: column MEGA-DEX B-SE, temperature program: 50 °C (10 min), then 150 °C (1.0 °C/min); retention time: enantiomer A: 32.2 min, enantiomer B: 33.2 min.

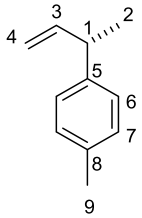


**M** ($C_{11}H_{14}$) 146.23 g/mol.

**$R_f$** 0.65 (*n*-pentane).

**$^1$H NMR** (300 MHz, $CDCl_3$): δ [ppm] = 7.12 (s, 4 H, H6, H7), 6.00 (d, *J* = 33.9 Hz, 1 H, H3), 5.04 (d, *J* = 22.1 Hz, 2 H, H4), 3.44 (p, *J* = 7.0 Hz, 1 H, H1), 2.33 (s, 3 H, H9), 1.35 (d, *J* = 7.0 Hz, 3 H, H2).

**$^{13}$C NMR** (75 MHz, $CDCl_3$): δ [ppm] = 143.6 (C3), 135.7 (C8), 129.2 (C7), 127.2 (C6), 113.0 (C4), 42.9 (C1), 21.1 (C9), 20.9 (C2).

**FT-IR** (ATR) ν [cm$^{-1}$]: 3080 (w), 3050 (w), 3022 (w), 2999 (w), 2966 (m), 2926 (w), 2871 (w), 1892 (w), 1831 (w), 1793 (w), 1636 (m), 1513 (s), 1454 (m), 1413 (m), 1369 (m), 1018 (m), 990 (m), 910 (vs), 815 (vs), 745 (m).

**GC-MS** (70 eV) m/z (%) = 146 (25, [M]$^+$), 131 (100, [M-CH$_3$]$^+$), 115 (25), 91 (35), 65 (10).

**[α]$_\lambda^{20}$** 49.5 ° (365 nm), 25.5 ° (436 nm), 12.7 ° (546 nm), 10.5 ° (579 nm), 10.1 ° (589 nm) (0.73 g/100 ml in CHCl$_3$).

The analytical data are in agreement with the literature.[4,5]

### 3.3.2. (*E*)-3-Iodo-2-methylprop-2-ene-1-ol (S7)[6–8]

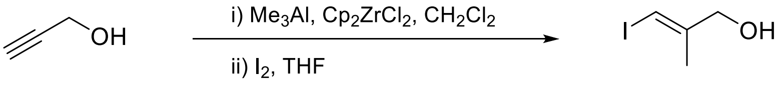


**11** **88 %** **S7**

Under inert gas atmosphere 1.96 g (6.7 mmol, 25 mol%) [Cp$_2$ZrCl$_2$] were dissolved in 50 ml of anhydrous dichloromethane and 40.0 ml (80.2 mmol, 3.0 eq) of a Me$_3$Al solution (2.0 M in hexanes) were added. Then the stirred solution was cooled to 0 °C and a solution of 1.55 ml (26.8 mol, 1.0 eq) of propargyl alcohol (**11**) in 22 ml of anhydrous dichloromethane were added dropwise over 1 h. Afterwards, the reaction mixture was allowed to warm to rt and stirred for further 17 h. Then, it was cooled to -30 °C and a solution of 8.15 g (32.1 mmol, 1.2 eq) of iodine in 26 ml of anhydrous THF was added over 1 h and the mixture was stirred for another 1 h until it was stopped by carefully adding a saturated aqueous NaHCO$_3$ solution. After removing the cooling bath and termination of gas evolution the mixture was diluted with further NaHCO$_3$ solution. The resulting suspension was filtrated, the layers were separated and the aqueous layer was extracted three times with MTBE. The combined organic layers were washed with saturated aqueous Na$_2$S$_2$O$_3$ solution, dried over MgSO$_4$ and the solvent was removed under reduced pressure. The residue was filtrated over silica gel (CyHex/EtOAc 2:1). After removal of the solvent 4.67 g (23.6 mmol, 88 %) of vinyliodide **S7** were obtained as a light yellow liquid.

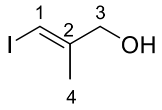


**M**(C$_4$H$_7$IO) 198.00 g/mol.

**R$_f$** 0.35 (CyHex/EtOAc 20:1).

**$^1$H NMR** (300 MHz, CDCl$_3$): δ [ppm] = 6.19 (s, 1 H, H1), 4.02 (s, 2 H, H3), 1.77 (s, 3 H, H4). O*H* could not be detected.

**$^{13}$C NMR** (75 MHz, CDCl$_3$): δ [ppm] = 147.1 (C2), 77.3 (C1), 66.8 (C3), 21.4 (C4).

**FT-IR** (ATR) ν [cm$^{-1}$] = 3304 (br, s), 2913 (m), 2852 (m), 1621 (w), 1433 (w), 1376 (m), 1275 (vs), 1252 (m), 1067 (s), 1010 (vs), 773 (vs).

**GC-MS** (70 eV) m/z (%) = 198 (30, [M]$^+$), 128 (20), 71 (55), 56 (30), 41 (100).

The analytical data are in agreement with the literature.[6,7]

### 3.3.3. (*E*)-3-Iodo-2-methylallyl acetate (12)[7,9]

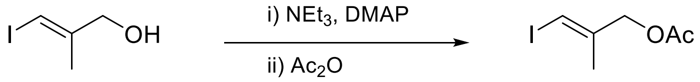


**S7** **96 %** **12**

To a solution of 3.37 g (17.1 mmol, 1.0 eq) of vinyliodide **S7** in 100 ml of anhydrous dichloromethane were added 7.1 ml (51.2 mol, 3.0 eq) of triethylamine followed by 0.42 g (3.4 mmol, 20 mol%) of DMAP under inert gas atmosphere. The reaction mixture was stirred for 30 min at rt and then cooled to 0 °C. At this temperature, 4.8 ml (51.2 mmol, 3.0 eq) of acetic anhydride were added dropwise. While stirring for 18 h, the reaction mixture was allowed to warm slowly to rt before saturated aqueous $NaHCO_3$ solution was added. After stirring for further 30 min at rt the layers were separated and the aqueous layer was extracted three times with dichloromethane. The combined organic layers were washed first with 1 M aqueous HCl and then with water. After drying over $MgSO_4$, the solvent was removed under reduced pressure and the residue was subjected to flash chromatography (silica gel, CyHex/EtOAc 10:1) to yield 3.94 g (16.4 mmol, 96 %) of acetate **12** as a light yellow oil.

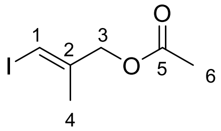

| | |
|---|---|
| **M**($C_6H_9IO_2$) | 240.04 g/mol. |
| **R$_f$** | 0.25 (CyHex/EtOAc 20:1). |
| **$^1$H NMR** | (300 MHz, $CDCl_3$): δ [ppm] = 6.35 (s, 1 H, H1), 4.56 (s, 2 H, H3), 2.09 (s, 3 H, H6), 1.86 (s, 3 H, H4). |
| **$^{13}$C NMR** | (75 MHz, $CDCl_3$): δ [ppm] = 170.5 (C4), 142.4 (C2), 80.3 (C1), 67.5 (C3), 21.8 (C2), 20.9 (C6). |
| **FT-IR** (ATR) | ν [cm$^{-1}$] = 3057 (w), 2921 (w), 2854 (w), 1738 (vs), 1376 (m), 1286 (m), 1220 (vs), 1054 (m), 1023 (s), 778 (m). |
| **GC-MS** (70 eV) | m/z (%) = 240 (5, [M]$^+$), 181 (10), 113 (45), 71 (10), 43 (100). |

The analytical data are in agreement with the literature.[7,9]

### 3.3.4. (2*S*,*E*)-2-Methyl-6-(*p*-tolyl)hept-2-ene-1-yl acetate (7a)

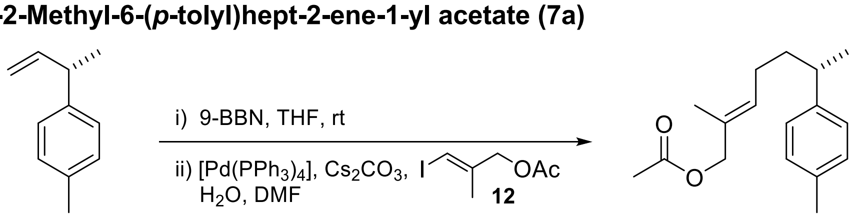


**8a** **91 %** **7a**

Under inert gas atmosphere 1.80 g (12.30 mmol, 1.0 eq) of olefin **8a** and 37.1 ml (18.49 mmol, 1.5 eq) of 9-BBN (0.5 M in THF) were dissolved in 100 ml of anhydrous THF and stirred at rt for 6 h. In a separate Schlenk flask, 9.17 g (24.60 mmol, 2.0 eq) $Cs_2CO_3$ were suspended in 50 ml of DMF under inert gas atmosphere and 3.38 g (14.17 mmol, 1.15 eq) of (*E*)-3-iodo-2-methylallyl acetate (**12**) were added. After stirring for 30 min at rt, 719 mg (0.62 mmol, 5 mol%) of $Pd(PPh_3)_4$ and 11.5 ml

(640 mmol, 52 eq) of $H_2O$ were added and the mixture was stirred for another 4 h at rt. Then the *in situ* generated borane from the first flask was added (by means of a syringe or transfer needle) and the resulting suspension was stirred for 18 h at 40 °C.

The mixture was then partitioned between saturated aqueous $NH_4Cl$ and MTBE. The layers were separated and the aqueous layer was extracted three times with MTBE. The combined organic layers were washed twice with water and dried over $MgSO_4$. After evaporation of the solvent and purification by column chromatography (CyHex/EtOAc 50:1) 2.92 g (11.2 mmol, 91 %) of nuciferyl acetate **7a** were obtained as a light yellow liquid.

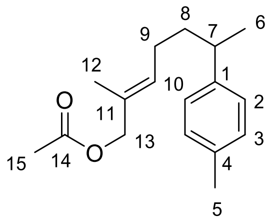


| | |
|---|---|
| **M**($C_{17}H_{24}O_2$) | 260.38 g/mol. |
| **R$_f$** | 0.11 (CyHex/EtOAc 50:1). |
| **$^1$H NMR** | (300 MHz, $CDCl_3$): δ [ppm] = 7.18-7.02 (m, 4 H, H2, H3), 5.44 (tq, *J* = 7.1, 1.3 Hz, 1 H, H10), 4.44 (s, 2 H, H13), 2.76-2.57 (m, 1 H, H7), 2.33 (s, 3 H, H5), 2.07 (s, 3 H, H15), 2.01-1.90 (m, 2 H, H9), 1.68 -1.59 (m, 2 H, H8), 1.57 (d, *J* = 1.4 Hz, 3 H, H12), 1.24 (d, *J* = 6.9 Hz, 3 H, H6). |
| **$^{13}$C NMR** | (75 MHz, $CDCl_3$): δ [ppm] = 171.1 (C14), 144.3 (C1), 135.4 (C4), 130.1 (C11), 129.9 (C10), 129.1 (C3), 127.0 (C2), 70.4 (C13), 39.2 (C7), 37.9 (C8), 26.0 (C9), 22.6 (C6), 21.1 (C5 und C15), 14.0 (C12). |
| **FT-IR** (ATR) | ν [cm$^{-1}$] = 2958 (m), 2922 (m), 2869 (m), 1737 (s), 1514 (m), 1453 (m), 1375 (m), 1223 (vs), 1020 (s), 815 (s). |
| **GC-MS** (70 eV) | m/z (%) = 260 (5, [M]$^+$), 200 (15), 185 (10), 157 (40), 143 (35), 132 (55), 119 (100). 105 (40), 91 (40), 41 (20). |
| **[α]$_λ^{20}$** | 74.3 ° (436 nm), 41.5 ° (546 nm), 35.9 ° (579 nm), 33.8 ° (589 nm) (0.88 g/100 ml in $CHCl_3$). |

### 3.3.5. (1*S*,4*R*)-1,6-Dimethyl-4-(prop-1-ene-2-yl)-1,2,3,4-tetrahydronaphthalene (*trans*-6a)

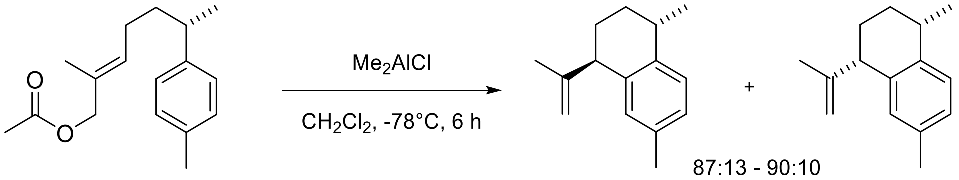


**7a** **97 %** *trans*-**6a** *cis*-**6a**

Under argon atmosphere 500 mg (1.90 mmol, 1.0 eq) of the nuciferyl acetate **7a** were dissolved in 50 ml of anhydrous dichloromethane. The solution was cooled to -78 °C before 4.75 ml (4.75 mmol, 2.5 eq) of a 1.0 M solution of $Me_2AlCl$ in hexanes were added dropwise over a period of 2 h and the

mixture was stirred for further 6 h at this temperature. Then the reaction was stopped by carefully adding some drops of MeOH and the cooling bath was removed. The product mixture was filtrated over silica and eluted with *n*-pentane. After removal of the solvent under reduced pressure, the residue was purified by column chromatography (*n*-pentane) to afford 370 mg (1.85 mmol, 97 %) of the substituted tetraline **6a** as a mixture of diastereomers (*trans*-**6a**/*cis*-**6a** = 9:1 according to chiral GC analysis) as a colorless liquid.

As the diastereomers were not separable by chromatography, the preparative separation was performed on a subsequent stage (see below). Analytical separation of *trans*-**6a** and *cis*-**6a**: column MEGA-DEX DET-Beta, temperature program: 50 °C (30 min), then heating to 120 °C (0.25 °C/min); retention time: *trans*-**6a**: 200.2 min, *cis*-**6a**: 215.3 min.

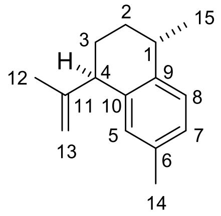


| | |
|---|---|
| **M**($C_{15}H_{20}$) | 200.32 g/mol. |
| **R$_f$** | 0.71 (*n*-pentane). |
| **$^1$H NMR** | (500 MHz, $CDCl_3$): δ [ppm] = 7.14 (dd, *J* = 19.0, 7.9 Hz, 1 H, H8), 7.03-6.97 (m, 1 H, H7), 6.95 (s, 1 H, H5), 4.96 (dd, *J* = 2.5, 1.4 Hz, H13a$_{cis}$), 4.93 (dd, *J* = 2.5, 1.4 Hz, H13a$_{trans}$), 4.72 (d, *J* = 2.3 Hz, H13b$_{cis}$), 4.69 (d, *J* = 2.3 Hz, H13b$_{trans}$), 3.52 (ddd, *J* = 14.1, 8.3, 5.8 Hz, 1 H, H4), 2.90 (p, *J* = 7.0 Hz, 1 H, H1), 2.31 (s, 3 H, H14), 1.97 (dddd, *J* = 22.9, 10.5, 5.1, 3.2 Hz, 2 H, H2a, H3a), 1.86-1.73 (m, 1 H, H3b), 1.69 (dd, *J* = 9.2, 1.2 Hz, 3 H, H12), 1.58-1.37 (m, 1 H, H2b), 1.31 (d, *J* = 7.0 Hz, 3 H, H15). |
| **$^{13}$C NMR** | (125 MHz, $CDCl_3$): δ [ppm] = 149.4 (C11), 139.5 (C9), 137.7 (C10), 134.2 (C6), 129.5 (C5), 127.4 (C8), 127.0 (C7), 113.5 (C13), 48.1 (C4), 32.7 (C1), 30.4 (C2), 26.7 (C3), 22.7 (C15), 21.1 (C14), 19.7 (C12). |
| **FT-IR** (ATR) | ν [$cm^{-1}$] = 3071 (w), 2957 (m), 2926 (s), 2862 (m), 1889 (w), 1785 (w), 1644 (m), 1498 (s), 1452 (s), 1373 (s), 890 (vs), 811 (vs). |
| **GC-MS** (70 eV) | m/z (%) = 200 (25, $[M]^+$), 185 (15), 157 (100), 128 (40), 91 (10). |
| **HRMS** (EI, 70 eV) | m/z: 200.157 u (calc.: m/z = 200.1565 u). |

The NMR data listed above are taken from the NMR spectra of the mixture of *trans*-**6a** and *cis*-**6a**.

### 3.3.6. 3-((1*R*,4*S*)-4,7-Dimethyl-1,2,3,4-tetrahydronaphthalene-1-yl)but-3-ene-1-ol (*trans*-13a)

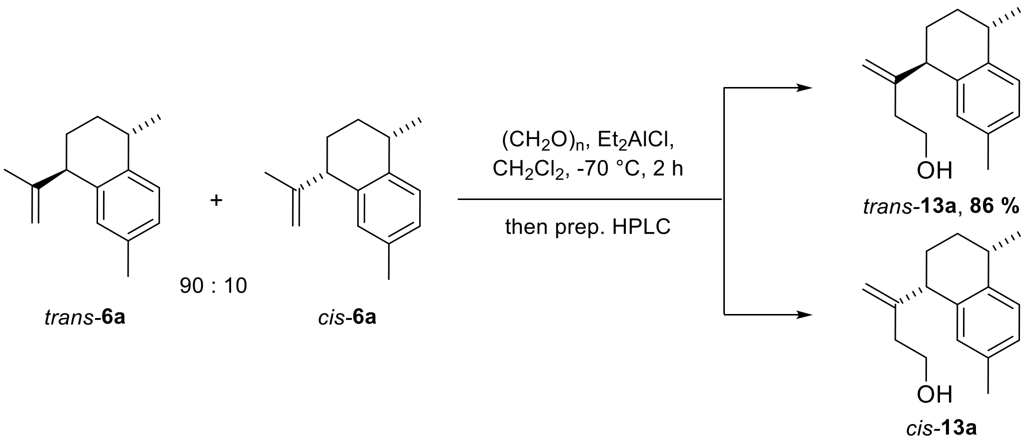


Under inert gas atmosphere 650 mg (3.25 mmol, 1.0 eq) of tetraline **6a** (as a mixture of *trans*-**6a**/*cis*-**6a** = 9:1) were dissolved in 65 ml of anhydrous dichloromethane and 146 mg (6.50 mmol, 1.5 eq) of *para*-formaldehyde were added. The mixture was stirred for 20 min at rt and then cooled to -70 °C. At this temperature, 6.50 ml (6.50 mmol, 2.0 eq) of a 1.0 M solution of $Et_2AlCl$ in hexanes was added dropwise. After stirring for 2 h at -70 °C the reaction was stopped by carefully adding a few drops of MeOH. After a quick filtration of the quenched reaction mixture over a small pad of silica (CyHex/EtOAc 1:1) and removal of the solvent under reduced pressure, the residue was purified by column chromatography (CyHex/EtOAc 5:1).

This pre-purified diastereomeric mixture (*trans*-**13a**/*cis*-**13a** = 9:1) could now be separated by preparative HPLC (Kromasil-5-CelluCoat, *n*-heptane/MTBE 3:1). To avoid losses due to the volatility of the product, only the MTBE part of the eluent was at first evaporated under reduced pressure. Then, a controlled amount of MeOH was added to generate a solvent ratio of *n*-heptane/MeOH = 60:40. This then allowed removal of the higher boiling solvent *n*-heptane as an azeotrope under comparably mild conditions (ca 350 mbar, 40 °C water bath). This way, 642 mg (2.79 mmol, 86 %) of the pure stereoisomer *trans*-**13a** were obtained as a light yellow liquid. In addition, a small analytical sample of the pure minor diastereomer *cis*-**13a** was isolated.

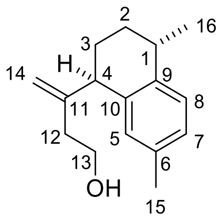


| | |
|---|---|
| **M**($C_{16}H_{22}O$) | 230.35 g/mol. |
| **R$_f$** | 0.22 (CyHex/EtOAc 5:1). |
| **$^1$H NMR** | (300 MHz, $CDCl_3$): δ [ppm] = 7.11 (t, *J* = 7.7 Hz, 1 H, H8), 6.97 (dd, *J* = 7.8, 1.9 Hz, 1 H, H7), 6.84 (s, 1 H, H5), 5.09-4.92 (m, 1 H, H14a), 4.79-4.62 (m, 1 H, H14b), 3.78 (d, *J* = 29.5 Hz, 2 H, H13), 3.51 (d, *J* = 30.5 Hz, 1 H, H4), 2.87 (h, *J* = 6.7 Hz, 1 H, H1), 2.35 (m, 2 H, H12), 2.27 (s, 3 H, H6), 2.04-1.89 (m, 2 H, H2a, H3a), 1.78-1.67 (m, 1 H, H3b), 1.46-1.39 (m, 1 H, H2b), 1.27 (d, *J* = 7.0 Hz, 3 H, H16). |

**$^{13}$C NMR** (75 MHz, $CDCl_3$): δ [ppm] = 150.1 (C11), 139.8 (C10), 137.4 (C9), 135.0 (C6), 129.9 (C5), 127.9 (C8), 127.2 (C7), 114.5 (C14), 61.2 (C13), 46.8 (C4), 36.8 (C12), 32.4 (C1), 29.1 (C2), 25.8 (C3), 23.0 (C16), 21.1 (C15).

**FT-IR** (ATR) ν [$cm^{-1}$] = 3354 (br, s), 2956 (s), 2927 (vs), 2865 (s), 1681 (m), 1641 (m), 1498 (m), 1452 (s), 1400 (m), 1370 (m), 1154 (m), 1044 (s), 894 (s), 813 (s).

**GC-MS** (70 eV) m/z (%) = 230 (15, $[M]^+$), 199 (30), 157 (100), 128 (5), 91 (15).

**HRMS** (EI, 70 eV) m/z: 230.167 u (calc.: m/z = 230.1671 u).

**$[\alpha]_\lambda^{20}$** -104.4 ° (365 nm), -48.2 ° (436 nm), -20.8 ° (546 nm), -17.1 ° (579 nm), -16.5 ° (589 nm) (0.49 g/100 ml in $CHCl_3$).

**Analytical data of *cis*-13a (minor diastereomer)**

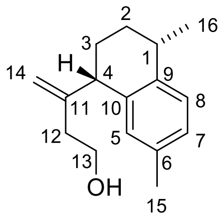


**M**($C_{16}H_{22}O$) 230.35 g/mol.

**$R_f$** 0.22 (CyHex/EtOAc 5:1).

**$^1$H NMR** (300 MHz, $CDCl_3$): δ [ppm] = 7.10 (d, *J* = 7.7 Hz, 1 H, H8), 6.97 (dd, *J* = 7.8, 1.9 Hz, 1 H, H7), 6.84 (s, 1 H, H5), 5.03 (t, *J* = 1.4 Hz, 1 H, H14a), 4.71 (s, 1 H, H14b), 3.79 (t, *J* = 6.6, 2 H, H13), 3.49 (t, *J* = 6.2 Hz, 1 H, H4), 2.87 (q, *J* = 6.5 Hz, 1 H, H1), 2.31 (dt, *J* = 6.6, 2.8 Hz, 2 H, H12), 2.26 (s, 3 H, H6), 1.92-1.73 (m, 2 H. H2a, H3a), 1.68-1.53 (m, 2 H, H3b), 1.45 (s, 1 H, H2b), 1.28 (d, *J* = 7.0 Hz, 3 H, H16).

**$^{13}$C NMR** (75 MHz, $CDCl_3$): δ [ppm] = 150.1 (C11), 139.8 (C10), 137.4 (C9), 135.0 (C6), 129.8 (C5), 128.3 (C8), 127.2 (C7), 114.8 (C14), 61.2 (C13), 46.9 (C4), 36.8 (C12), 32.4 (C1), 28.5 (C2), 25.2 (C3), 23.3 (C16), 21.1 (C15).

**FT-IR** (ATR) ν [$cm^{-1}$] = 3354 (br, s), 3072 (w), 2956 (s), 2927 (vs), 2865 (s), 1681 (m), 1641 (m), 1498 (m), 1456 (s), 1400 (m), 1369 (m), 1152 (m), 1043 (s), 894 (s), 813 (s).

**GC-MS** (70 eV) m/z (%) = 230 (15, $[M]^+$), 199 (30), 157 (100), 128 (5), 91 (15).

**$[\alpha]_\lambda^{20}$** 64.5 ° (436 nm), 35.2 ° (546 nm), 30.5 ° (579 nm), 29.4 ° (589 nm) (0.96 g/100 ml in $CHCl_3$).

### 3.3.7. (*S*)-3-((1*R*,4*S*)-4,7-Dimethyl-1,2,3,4-tetrahydronaphthalene-1-yl)-butane-1-ol (15a)

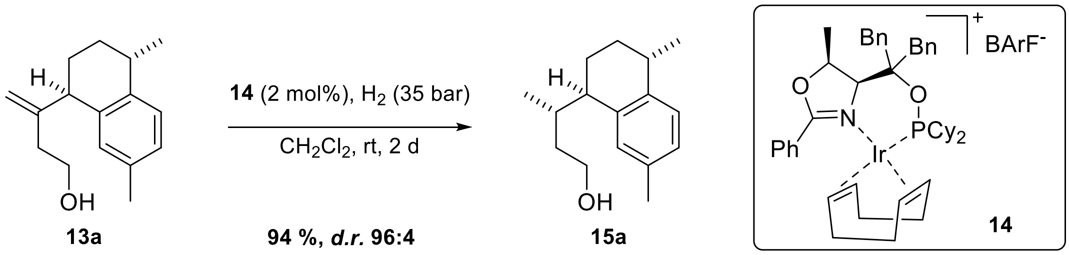


An oven-dried vial was charged under argon with a stirring bar and a solution of 90 mg of *trans*-**13a** (0.39 mmol, 1.0 eq) in 4 ml of anhydrous dichloromethane and 14 mg (0.078 mmol, 2 mol%) of the *Pfaltz* catalyst **14** ([((4*S*,5*S*)-$Cy_2$-Ubaphox)Ir(COD)]BArF). Then the vial was positioned in a pressure reactor, which was subsequently flushed with 35 bar of $H_2$. Afterwards the magnetically stirred reaction mixture was hydrogenated at rt (at 35 bar $H_2$) for 48 h. After careful depressurizing, GC-MS analysis of a sample indicated full conversion of the starting material and a diastereoselectivity of 96:4. The solvent was then removed under reduced pressure and the residue purified by column chromatography (CyHex/EtOAc 5:1) to yield 85 mg of the pure main isomer (*S*,*R*,*S*)-**15a** (0.37 mmol, 94 %) as a colorless oil.

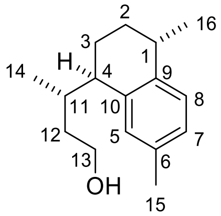

| | |
|---|---|
| **M**($C_{16}H_{24}O$) | 232.37 g/mol. |
| **$R_f$** | 0.22 (CyHex/EtOAc 5:1). |
| **$^1$H NMR** | (600 MHz, $CDCl_3$): δ [ppm] = 7.14 (dd, *J* = 8.0, 1.0 Hz, 1 H, H8), 7.06 (s, 1 H, H5), 6.98-6.93 (m, 1 H, H7), 3.84-3.67 (m, 2 H, H13), 2.90-2.79 (m, 1 H, H4), 2.74 (dq, *J* = 11.4, 6.4 Hz, 1 H, H1), 2.35-2.31 (m, 1 H, H11), 2.30 (s, 3 H, H15), 1.94 (dddd, *J* = 12.6, 5.9, 4.9, 3.3 Hz, 1 H, H2a), 1.83 (dtd, *J* = 13.0, 6.4, 3.3 Hz, 1 H, H3a), 1.72 (dtd, *J* = 13.0, 7.3, 5.6 Hz, 1 H, H12a), 1.62-1.54 (m, 2 H, H3b, H12b), 1.33 (dddd, *J* = 13.0, 11.4, 9.7, 3.4 Hz, 1 H, H2b), 1.27 (d, *J* = 6.9 Hz, 3 H, H16), 0.68 (d, *J* = 6.8 Hz, 3 H, H14). |
| **$^{13}$C NMR** | (150 MHz, $CDCl_3$): δ [ppm] = 140.5 (C9), 139.5 (C10), 134.9 (C6), 128.3 (C5), 126.6 (C8), 126.3 (C7), 61.6 (C13), 42.2 (C4), 38.2 (C12), 33.7 (C11), 32.9 (C1), 31.8 (C2), 22.0 (C16), 21.9 (C3), 21.3 (C15), 14.5 (C14). |
| **FT-IR** (ATR) | ν [$cm^{-1}$] = 3361 (s, br), 2955 (s), 2926 (vs), 2871 (m), 2855 (m), 1734 (w), 1718 (m), 1498 (m), 1453 (m), 1375 (m), 1165 (m), 1059 (m), 813 (m). |
| **GC-MS** (70 eV) | m/z (%) = 232 (15, $[M]^+$), 214 (5), 199 (10), 159 (100),143 (15), 128 (15). |
| **HRMS** (EI, 70 eV) | m/z: 232.181 u (calc.: m/z = 232.1827 u). |
| **$[\alpha]_\lambda^{20}$** | 56.4 ° (436 nm), 37.0 ° (546 nm), 33.6 ° (579 nm), 32.0 ° (589 nm) (0.55 g/100 ml in $CHCl_3$). |

The analytical data are in agreement with the literature.[11]

### 3.3.8. (1*S*,4*R*)-4-((*S*)-4-Iodobutane-2-yl)-1,6-dimethyl-1,2,3,4-tetrahydronaphthalene (16)[4]

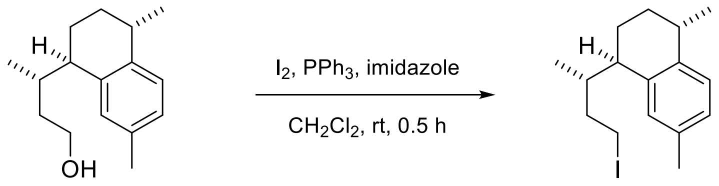


**15a** **91 %** **16**

To a solution of 40 mg (0.17 mmol, 1.0 eq) of alcohol **15a** in 2.5 ml of anhydrous dichloromethane were added 57.6 mg (0.22 mmol, 1.3 eq) of $PPh_3$, followed by 21.2 mg (0.31 mmol, 1.2 eq) of imidazole and 53.3 mg (0.21 mmol, 1.2 eq) of iodine. A color change of the reaction suspension from yellow to dark red was observed. After stirring for 30 min at rt the solvent was removed under reduced pressure and the residue was subjected to column chromatography (CyHex/EtOAc 50:1) to yield 53 mg (0.15 mmol, 91 %) of the iodide **16** as a light-sensitive colorless solid.

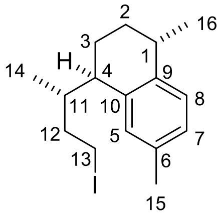


| | |
|---|---|
| **M**($C_{16}H_{23}I$) | 342.26 g/mol. |
| **R$_f$** | 0.77 (CyHex/EtOAc 50:1). |
| **$^1$H NMR** | (600 MHz, $CDCl_3$) δ [ppm] = 7.15 (dd, *J* = 7.9, 1.0 Hz, 1 H, H8), 7.06 (d, *J* = 1.8 Hz, 1 H, H5), 6.98 (dd, *J* = 7.9, 1.8 Hz, 1 H, H7), 3.33 (ddd, *J* = 9.6, 8.1, 6.5 Hz, 1 H, H13a), 3.26 (ddd, *J* = 9.6, 8.1, 6.7 Hz, 1 H, H13b), 2.89 (td, *J* = 7.9, 4.3 Hz, 1 H, H4), 2.75 (dq, *J* = 11.1, 6.5 Hz, 1 H, H1), 2.33 (s, 3 H, H15), 2.31-1.27 (m, 1 H, H11), 1.96 (d, *J* = 47.9 Hz, 2 H, H12), 1.90-1.83 (m, 1 H, H2a), 1.80 (ddt, *J* = 13.1, 6.4, 3.2 Hz, 1 H, H3a), 1.57 (dddd, *J* = 12.8, 11.3, 9.0, 3.5 Hz, 1 H, H3b), 1.34 (dddd, *J* = 13.0, 11.3, 9.7, 3.4 Hz, 1 H, H2b), 1.28 (d, *J* = 6.9 Hz, 3 H, H16), 0.69 (d, *J* = 6.8 Hz, 3 H, H14). |
| **$^{13}$C NMR** | (150 MHz, $CDCl_3$): δ [ppm] = 140.5 (C9), 139.0 (C10), 135.0 (C6), 128.2 (C5), 126.7 (C8), 126.5 (C7), 41.0 (C4), 39.0 (C12), 38.5 (C11), 32.9 (C1), 31.7 (C2), 22.0 (C16), 21.9 (C3), 21.3 (C15), 14.0 (C14), 5.9 (C13). |
| **FT-IR** (ATR) | ν [cm$^{-1}$] = 3004 (w), 2957 (s), 2924 (vs), 2869 (m), 2855 (m), 1893 (w), 1732 (w), 1612 (w), 1497 (m), 1453 (m), 1376 (m), 1244 (m), 1180 (m), 812 (s). |
| **GC-MS** (70 eV) | m/z (%) = 342 (10, [M]$^+$), 214 (10), 199 (10), 159 (100), 143 (15), 128 (29), 105 (10), 77 (5). |
| **HRMS** (EI, 70 eV) | m/z: 342.084 u (calc.: m/z = 342.0844 u). |
| **[α]$_λ^{20}$** | 12.1 ° (436 nm), 15.1 ° (546 nm), 14.9 ° (579 nm), 13.1 ° (589 nm) (0.30 g/100 ml in $CHCl_3$). |

The analytical data are in agreement with the literature.[11]

### 3.3.9. (1*S*,4*R*)-1,6-Dimethyl-4-((*S*)-6-methylhept-5-ene-2-yl)-1,2,3,4-tetrahydronaphthalene (1)

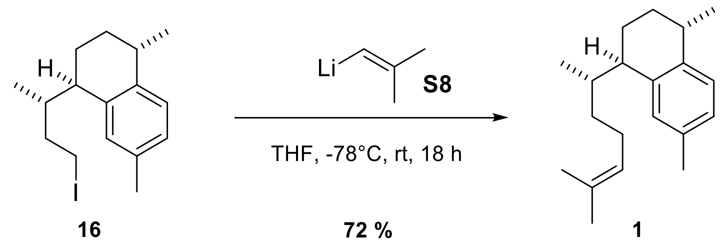


Preparation of a stock solution of isocrotyl-lithium (**S8**):[12] To an excess of elementary lithium (ca. 20 eq) in 3 ml of anhydrous $Et_2O$ was carefully added under argon a solution of 0.5 ml (4.9 mmol) of 1-bromo-2-methylpropene in 1 ml of anhydrous $Et_2O$. During the whole period of addition (5-10 min) a slight reflux was observed. After complete addition the mixture was stirred for 1 h at rt before it was cooled to -40 °C and then diluted with 15 ml of anhydrous THF.

Under an atmosphere of argon, 60 mg (0.18 mmol, 1.0 eq) of the iodide **16** were dissolved in 1.5 ml of anhydrous THF and cooled to -78 °C. Then, 1.52 ml (0.36 mmol, 2.0 eq) of the stock solution of **S8** (about 0.23 M in THF)[12] were added slowly by means of a syringe containing glass wool.[12] The reaction mixture was stirred for 18 h while it was allowed to slowly warm to rt. Excess reagent was quenched by careful addition of water and the aqueous layer was extracted three times with MTBE. The combined organic layers were dried over $MgSO_4$ and the solvent was evaporated under reduced pressure. The residue was purified by column chromatography (*n*-pentane) to yield 35 mg (0.13 mmol, 72 %) of (+)-erogorgiaene (**1**) as a colorless oil.

The expected absolute and relative configuration of the product was confirmed by comparing the NMR and optical rotation data with literature values.[13,14]

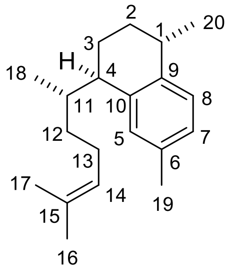


| | |
|---|---|
| **M**($C_{20}H_{30}$) | 270.46 g/mol. |
| **R$_f$** | 0.66 (CyHex). |
| **$^1$H NMR** | (600 MHz, $CDCl_3$): δ [ppm] = 7.14 (dd, *J* = 7.9, 1.0 Hz, 1 H, H8), 7.03 (s, 1 H, H5), 6.95 (dd, *J* = 7.9, 1.9 Hz, 1 H, H7), 5.18 (ddq, *J* = 8.5, 5.7, 1.4 Hz, 1 H, H14), 2.93-2.84 (m, 1 H, H4), 2.73 (dp, *J* = 11.6, 6.0 Hz, 1 H, H1), 2.31 (s, 3 H, H19), 2.18-2.01 (m, 3 H), 1.97-1.89 (m, 1 H, H2a), 1.82 (dddd, *J*= 3.0, 6.8, 6.0, 3.3 Hz, 1 H; H3a), 1.73 (d, *J* = 1.2 Hz, 3 H, H16), 1.64 (d, *J* = 1.3 Hz, 3 H, H17), 1.59-1.52 (m, 1 H, H3b), 1.54-1.40 (m, 1 H, H12a), 1.40-1.30 (m, 2 H, H2b, H12b),1.27 (d, *J* = 6.9 Hz, 3 H, H20), 0.65 (d, *J* = 6.8 Hz, 3 H, H18). |

| | |
|---|---|
| **$^{13}$C NMR** | (150 MHz, $CDCl_3$): δ [ppm] = 140.4 (C9), 139.9 (C10), 134.7 (C6), 131.3 (C15), 128.1 (C5), 126.4 (C8), 126.0 (C7), 124.9 (C14), 41.4 (C4), 37.0 (C11), 35.2 (C12), 32.9 (C1), 31.8 (C2), 26.3 (C13), 25.8 (C16), 21.9 (C20), 21.6 (C3), 21.2 (C19), 17.7 (C17), 14.6 (C18). |
| **FT-IR** (ATR) | ν [$cm^{-1}$] = 2954 (s), 2922 (vs), 2853 (s), 1741 (m), 1713 (m), 1669 (m), 1497 (m), 1455 (s), 1376 (s), 1260 (m), 1164 (m), 1088 (w), 1021 (w), 810 (s). |
| **GC-MS** (70 eV) | m/z (%) = 270 (20, $[M]^+$), 186 (50), 159 (100), 143 (20), 128 (20), 41 (20). |
| **HRMS** (EI, 70 eV) | m/z: 270.232 u (calc.: m/z = 270.2347 u). |
| **$[\alpha]_\lambda^{20}$** | 26.1 ° (436 nm), 24.6 ° (546 nm), 22.2 ° (579 nm), 22.0 ° (589 nm) (0.18 g/100 ml in $CHCl_3$). |

The analytical data are in agreement with the literature.[11,13,15]

## 3.4. Synthesis of the pseudopterosin A-F aglycone (20)

### 3.4.1. (*S*)-1-(But-3-en-2-yl)-2,3-dimethoxy-4-methylbenzene (8b)

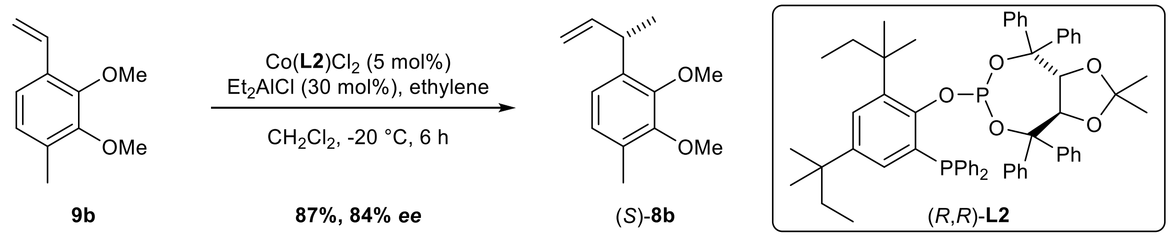


**9b** **87%, 84% *ee*** (*S*)-**8b** (*R*,*R*)-**L2**

A dried 100 ml Schlenk flask was charged with 97.4 mg (0.75 mmol, 5 mol%) of water free $CoCl_2$ and dissolved in 25 ml of anhydrous THF under inert atmosphere. Meanwhile, a second flask was charged with 684.8 mg (0.75 mmol, 1.0 eq) of Ligand **L2** and dissolved in 10 ml of anhydrous THF. After stirring for 0.5 h the solution containing the ligand was added to the flask containing $CoCl_2$ by means of a transfer needle. The resulting solution was stirred for 14 h before the solvent was removed under reduced pressure (vacuum line). The evacuated flask was flooded with ethylene (1.2 bar) and the complex was dissolved in 37.5 ml of anhydrous $CH_2Cl_2$ before cooling to -20 °C. The solution was stirred for 5 min before 4.5 ml (1.0 M in hexane, 4.50 mmol, 30 mol%) of $Et_2AlCl$ were added dropwise over 10 min. After this 2.674 g (15.00 mmol, 1.0 eq) of styrene derivative **9b** were added in one portion and stirring was continued for further 6 h at -20 °C. The reaction was quenched with 1 ml of MeOH and allowed to warm to rt. After addition of 30 ml of 1 M HCl the layers were separated, the aqueous layer was extracted with 3 x 30 ml of $CH_2Cl_2$ and the combined organic layers were dried over $MgSO_4$. After filtration, the solvent was removed under reduced pressure and the crude product was purified by flash column chromatography (CyHex/EtOAc 25:1) and distillation (0.08 mbar, 70 °C) to yield 2.692 g (13.05 mmol, 87%, 84% *ee*) of **8b** as a colorless oil.

Besides the desired branched hydrovinylation product **8b** also minor amounts of the linear regioisomer were detected (97:3, determined by GC-MS FID of crude product). Furthermore, during the reaction higher amounts of polyethylene were produced, which could be removed by means of column chromatography.

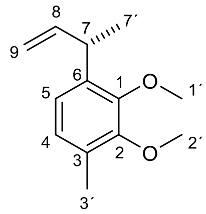

| | |
|---|---|
| **M** ($C_{13}H_{18}O_2$) | 206.29 g/mol. |
| **R$_f$** | 0.45 (CyHex/EtOAc 20:1). |
| **$^1$H NMR** | (500 MHz, $CDCl_3$): δ [ppm] = 6.95 (d, $J$ = 7.9 Hz, 1 H, H4 or H5), 6.90 (d, $J$ = 7.9 Hz, 1 H, H4 or H5), 6.10 (ddd, $J$ = 16.6, 10.3, 6.1 Hz, 1 H, H8), 5.14-5.07 (m, 2 H, H9), 4.00-3.95 (m, 1 H, H7), 3.93 (s, 3 H, H2´), 3.91 (s, 3 H, H1´), 2.32 (s, 3 H, H3´), 1.40 (d, $J$ = 7.1 Hz, 3 H, H7´). |
| **$^{13}$C NMR** | (125 MHz, $CDCl_3$): δ [ppm] = 151.4 (C1), 150.6 (C2), 143.4 (C8), 137.3 (C6), 130.0 (C3), 125.7 (C4), 122.3 (C5), 112.9 (C9), 60.7 (C1´or C2´), 59.9 (C1´or C2´), 35.9 (C7), 20.5 (C7´), 15.7 (C3´). |
| **FT-IR** (ATR) | ν [cm$^{-1}$]: 3080 (w), 2965 (w), 2931 (w), 2868 (w), 2828 (w), 1636 (w), 1603 (w), 1575 (w), 1490 (w), 1461 (m), 1407 (s), 1376 (w), 1276 (s), 1220 (m), 1176 (w), 1060 (s), 1022 (s), 998 (m), 908 (s), 837 (w), 814 (m), 789 (m), 748 (w), 695 (w), 657 (w), 630 (w). |
| **GC-MS** (70 eV) | m/z (%) = 206 (92, $[M]^+$), 191 (100), 177 (42), 160 (52), 145 (20), 131 (24), 105 (28), 91 (39), 77 (28), 51 (24). |
| **HRMS** (ESI) | m/z: 229.1203 u (calc.: m/z = $[M+Na]^+$: 229.1199 u). |
| **$[\alpha]_\lambda^{20}$** | -120.4 ° (334 nm), -89.6 ° (365 nm), -65.8 ° (405 nm), -30.3 ° (546 nm), -25.4 ° (589 nm) (1.66 g/100 ml in $CHCl_3$). |

The analytical data are in agreement with the literature.[3]

### 3.4.2. (*S,E*)-6-(2,3-Dimethoxy-4-methylphenyl)-2-methylhept-2-en-1-yl acetate (7b)

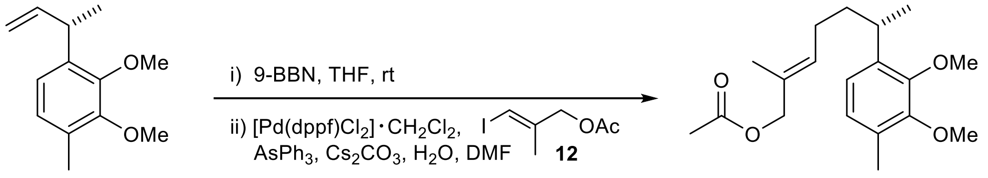


**8b** **89 %** **7b**

200 mg (0.97 mmol, 1.0 eq) of olefin **8b** were dissolved in 7.6 ml of anhydrous THF before 2.9 ml (0.5 M in THF, 1.45 mmol, 1.5 eq) of 9-BBN were added. The solution was stirred for 24 h. Meanwhile, a microwave vial was charged with a solution of 279 mg (1.16 mmol, 1.2 eq) of the vinyl iodide **11** in 9 ml of anhydrous DMF and 632 mg (1.94 mmol, 2.0 eq) of $Cs_2CO_3$ were added. After 30 min stirring at rt, 15 mg (0.05 mmol, 5 mol%) of $AsPh_3$, 40 mg (0.05 mmol, 5 mol%) of $[Pd(dppf)Cl_2]$ • $CH_2Cl_2$ and 0.7 mL (38.78 mmol, 40.0 eq) of $H_2O$ were added subsequently. The mixture was stirred for 4 h at rt before the solution of the *in situ* prepared borane was added by means of a transfer needle. The resulting reaction mixture was stirred for 2 h at 40 °C and 50 W under microwave irradiation before it was cooled to rt and diluted with 10 ml of MTBE and 15 ml of a saturated $NH_4Cl$ solution. The layers

were separated, the aqueous layer was extracted with 3 x 15 ml of MTBE and the combined organic layers were washed with 2 x 10 ml of $H_2O$ and dried over $MgSO_4$. After filtration, the solvent was removed under reduced pressure and the crude product was purified by flash column chromatography (CyHex/EtOAc 20:1) to yield 277 mg (0.86 mmol, 89%) of **7b** as a yellow oil.

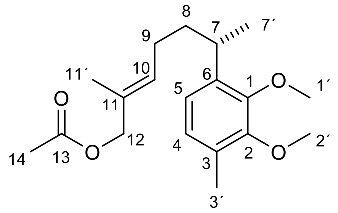

| | |
|---|---|
| **M**($C_{19}H_{28}O_4$) | 320.43 g/mol. |
| **R$_f$** | 0.34 (CyHex/EtOAc 10:1). |
| **$^1$H NMR** | (300 MHz, $CDCl_3$): δ [ppm] = 6.87 (d, *J* = 7.9 Hz, 1 H, H4 or H5), 6.82 (d, *J* = 7.9 Hz, 1 H, H4 or H5), 5.46 (t, *J* = 7.9 Hz, 1 H, H10), 4.43 (s, 2 H, H12), 3.83 (s, 3 H, H1´or H2´), 3.82 (s, 3 H, H1´or H2´), 3.13 (tq, *J* = 7.0 Hz, 1 H, H7), 2.24 (s, 3 H, H3´), 2.06 (s, 3 H, H14), 2.04-1.89 (m, 2 H, H9), 1.66-1.59 (m, 2 H, H8), 1.58 (s, 3 H, C11´), 1.20 (d, *J* = 7.0 Hz, 3 H, H7´). |
| **$^{13}$C NMR** | (75 MHz, $CDCl_3$): δ [ppm] = 171.0 (C13), 151.3 (C2), 150.8 (C1), 138.8 (C6), 129.9 (C10), 129.9 (C11), 129.5 (C3), 125.6 (C4), 121.4 (C5), 70.3 (C12), 60.6 (C1´or C2´), 59.9 (C1´ or C2´), 37.2 (C8), 31.7 (C7), 26.1 (C9), 22.0 (C7´), 21.0 (C14), 15.7 (C3´), 13.9 (C11´). |
| **FT-IR** (ATR) | ν [cm$^{-1}$] = 2959 (w), 2931 (w), 2863 (w), 2827 (w), 1738 (s), 1491 (w), 1459 (m), 1408 (m), 1376 (w), 1276 (m), 1223 (s), 1066 (m), 1049 (m), 1023 (s), 915 (w), 876 (w), 813 (m), 789 (w), 652 (w), 607 (w). |
| **GC-MS** (70 eV) | m/z (%) = 320 (6, [M]$^+$), 260 (35), 245 (20), 229 (14), 217 (30), 203 (17), 179 (100), 164 (27), 149 (20), 128 (11), 115 (14), 91 (18), 77 (11). |
| **HRMS** (ESI) | m/z: 343.1883 u (calc.: m/z = [M+Na]$^+$: 343.1880 u). |
| **[α]$_λ^{20}$** | -0.4 ° (589 nm) (0.98 g/100 ml in $CHCl_3$). |

The analytical data are in agreement with the literature.[1,10]

### 3.4.3. (1*R*,4*S*)-5,6-Dimethoxy-4,7-dimethyl-1-(prop-1-en-2-yl)-1,2,3,4-tetrahydronaphthalene (*trans*-6b)

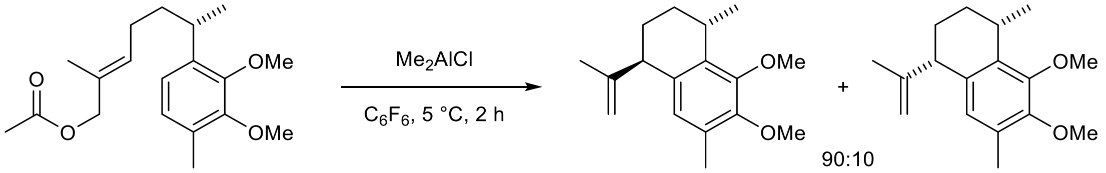


**7b** **94 %** *trans*-**6b** *cis*-**6b**

800 mg (2.50 mmol, 1.0 eq) of allyl acetate **7b** were dissolved in 76.2 ml of anhydrous hexafluorobenzene and cooled to 5 °C before 6.9 ml (0.9 M in heptane, 6.25 mmol, 2.5 eq) of $Me_2AlCl$ were added dropwise over 30 min. Stirring was continued for 1.5 h at 5 °C before the reaction mixture was quenched by adding 0.4 ml of methanol and the cooling bath was removed. The crude product

was purified by filtration over silica gel (CyHex/EtOAc 10:1) to yield 612 mg (2.35 mmol, 94%) of **6b** as a diastereomeric mixture (*d.r.* = 90:10, according to $^{1}$H NMR) in favor of the desired diastereomer *trans*-**6b**.

As the diastereomers were not separable by chromatography, the separation was performed on a subsequent stage (see below).

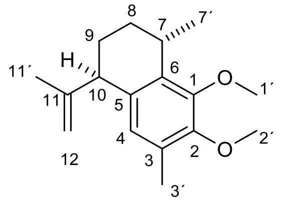


| | |
|---|---|
| **M**($C_{17}H_{24}O_2$) | 260.38 g/mol. |
| **R$_f$** | 0.40 (CyHex/EtOAc 25:1). |
| **$^{1}$H NMR** | (500 MHz, $CDCl_3$): δ [ppm] = 6.59 (s, 1 H, H4), 4.83 (s, 1 H, H12$_{trans}$), 4.21 (s, 1 H, H12$_{cis}$), 3.88 (s, 3 H, H1´), 3.80 (s, 3 H, H2´), 3.37-3.32 (m, 1 H, H10), 3.14-3.05 (m, 1 H, H7), 2.19 (s, 3 H, H3´), 2.03-1.83 (m, 2 H, H8b, H9b), 1.76 (s, 3 H, C11´), 1.69-1.61 (m, 1 H, H9a), 1.47-1.42 (m, 1 H, H8a), 1.21 (d, *J* = 6.9 Hz, 3 H, H7´). |
| **$^{13}$C NMR** | (125 MHz, $CDCl_3$): δ [ppm] = 150.6 (C1), 150.4 (C11), 149.4 (C2), 134.6 (C6), 133.9 (C5), 129.1 (C3), 127.2 (C4), 113.5 (C12), 60.5 (C1´), 59.9 (C2´), 45.4 (C10), 27.5 (C7), 26.2 (C8), 22.2 (C9), 22.1 (C7´), 21.9 (C11´), 15.8 (C3´). |
| **FT-IR** (ATR) | ν [cm$^{-1}$] = 3075 (w), 2956 (w), 2933 (m), 2864 (w), 2827 (w), 1644 (w), 1608 (w), 1573 (w), 1479 (m), 1449 (m), 1406 (m), 1373 (w), 1343 (w), 1320 (m), 1276 (m), 1233 (m), 1180 (w), 1115 (w), 1072 (s), 1017 (m), 957 (w), 918 (m), 895 (m), 814 (w), 790 (w)m 739 (w), 684 (w), 642 (w), 607 (w). |
| **GC-MS** (70 eV) | m/z (%) = 260 (63, [M]$^{+}$), 245 (27), 231 (28), 217 (100), 203 (34), 187 (35), 173 (18), 157 (14), 143 (14), 128 (24), 105 (7), 91 (15), 77 (8), 53 (4). |
| **HRMS** (ESI) | m/z: 283.1674 u (calc.: m/z = [M+Na]$^{+}$: 283.1668 u). |
| **$[\alpha]_{\lambda}^{20}$** | -16.8 ° (589 nm) (0.69 g/100 ml in $CHCl_3$). |

The NMR data listed above are taken from the NMR spectra of the mixture of *trans*-**6b** and *cis*-**6b**. The analytical data are in agreement with those of a sample of known absolute configuration prepared earlier *via* a different route.[1]

### 3.4.4. 3-((1*R*,4*S*)-5,6-Dimethoxy-4,7-dimethyl-1,2,3,4-tetrahydronaphthalen-1-yl)but-3-en-1-ol (13b)

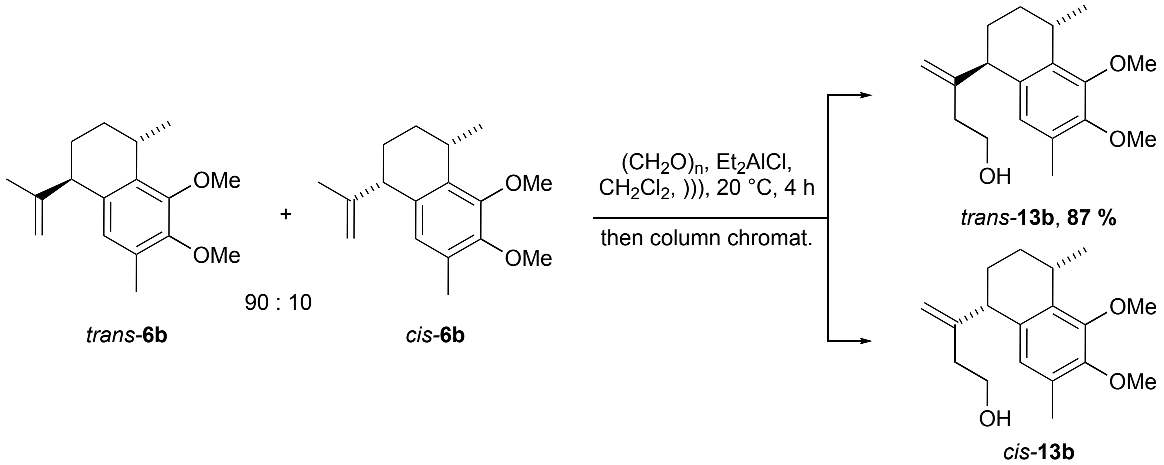


705 mg (2.71 mmol, 1.0 eq) of olefin **6b** (as a diastereomeric mixture of *trans*-**6b**/*cis*-**6b** 90:10) were dissolved in 7.1 ml of anhydrous $CH_2Cl_2$ before 122 mg (4.06 mmol, 1.5 eq) of finely ground paraformaldehyde were added and put in an ultrasonic bath for 20 min at rt. Following this, 5.4 ml (1.0 M in hexane, 5.42 mmol, 2.0 eq) $Et_2AlCl$ were added dropwise and the resulting yellow solution was left in the ultrasonic bath for 4 h at 20 °C. The reaction mixture was quenched by adding 0.2 ml methanol carefully. After filtration over silica gel (CyHex/EtOAc 1:1), the solvent was removed under reduced pressure and the crude product was purified by flash column chromatography (CyHex/MTBE 2:1) to yield 685 mg (2.36 mmol, 87%) of *trans*-**13b** as a diastereomerically pure compound.

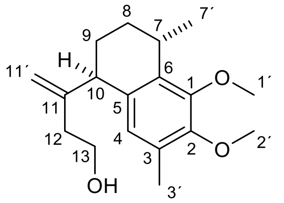


| | |
|---|---|
| **M**($C_{18}H_{26}O_3$) | 290.40 g/mol. |
| **R**$_f$ | 0.28 (CyHex/MTBE 2:1). |
| **$^1$H NMR** | (500 MHz, $CDCl_3$): δ [ppm] = 6.50 (s, 1 H, H4), 4.94 (s, 1 H, H11´$_{trans}$), 4.28 (s, 1 H, H11´$_{cis}$), 3.88 (s, 3 H, H1´), 3.84-3.80 (m, 2 H, H13), 3.80 (s, 3 H, H2), 3.40 (Ψd, *J* = 5.2 Hz, 1 H, H10), 3.16-3.06 (m, 1 H, H7), 2.43-2.31 (m, 2 H, H12), 2.18 (s, 3 H, H3´), 1.99 (dt, *J* = 4.5, 2.3 Hz, 1 H, H8b), 1.89 (tdd, *J* = 13.4, 5.5, 2.5 Hz, 1 H, H9b), 1.63 (dt, *J* = 4.5, 2.3 Hz, 1 H, H8a), 1.44 (ddt, *J* = 13.4, 5.5, 2.5 Hz, 1 H, H9a), 1.20 (d, *J* = 6.9 Hz, 3 H, H7´). The hydroxyl group could not be detected. |
| **$^{13}$C NMR** | (125 MHz, $CDCl_3$): δ [ppm] = 150.9 (C11), 150.6 (C1), 149.4 (C2), 134.8 (C6), 133.4 (C5), 129.4 (C3), 127.3 (C4), 115.0 (C11´), 60.9 (C13), 60.5 (C1´), 59.9 (C2´), 43.9 (C10), 38.3 (C12), 27.3 (C7), 25.3 (C8), 22.1 (C7´), 21.7 (C9), 15.8 (C3´). |

| | |
|---|---|
| **FT-IR** (ATR) | ν [cm$^{-1}$] = 2933 (m), 2864 (w), 1643 (w), 1481 (m), 1449 (m), 1402 (m), 1373 (w), 1323 (m), 1273 (w), 1234 (m), 1179 (w), 1074 (s), 1040 (s), 1019 (s), 904 (m), 802 (m), 733 (s), 644 (w), 531 (w). |
| **GC-MS** (70 eV) | m/z (%) = 290 (100, [M]$^+$), 275 (31), 259 (87), 241 (30), 217 (77), 202 (36), 187 (43), 165 (49), 143 (38), 128 (81), 107 (10), 91 (58), 67 (20), 41 (64). |
| **HRMS** (ESI) | m/z: 313.1778 u (calc.: m/z = [M+Na]$^+$: 313.1774 u). |
| **$[\alpha]_\lambda^{20}$** | -6.6 ° (589 nm) (0.62 g/100 ml in $CHCl_3$). |

**Analytical data of *cis*-13b (minor diastereomer)**

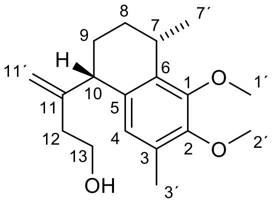


| | |
|---|---|
| **M**($C_{18}H_{26}O_3$) | 290.40 g/mol. |
| **$R_f$** | 0.32 (CyHex/MTBE 2:1). |
| **$^1$H NMR** | (500 MHz, $CDCl_3$): δ [ppm] = 6.67 (s, 1 H, H4), 5.03-4.99 (m, 2 H, H11´), 3.89 (s, 3 H, H1´), 3.80 (s, 3 H, H2´), 3.79-3.72 (m, 2 H, H13), 3.46 (dd, *J* = 10.7, 5.7 Hz, 1 H, H10), 3.20-3.11 (m, 1 H, H7), 2.27-2.20 (m, 2 H, H12), 2.18 (s, 3 H, H3´), 1.94-1.67 (m, 4 H, H8 and H9), 1.24 (d, *J* = 7.0 Hz, 3 H, H7´). The hydroxyl group could not be detected. |
| **$^{13}$C NMR** | (125 MHz, $CDCl_3$): δ [ppm] = 150.7 (C11), 149.6 (C1), 149.4 (C2), 134.9 (C6), 132.9 (C5), 129.4 (C3), 125.4 (C4), 113.6 (C11´), 61.5 (C13), 60.4 (C1´), 59.9 (C2´), 48.4 (C10), 35.2 (C12), 29.5 (C8), 27.5 (C7), 24.3 (C9), 22.2 (C7´), 15.9 (C3´). |
| **FT-IR** (ATR) | ν [cm$^{-1}$] = 2933 (m), 2864 (w), 1643 (w), 1481 (m), 1449 (m), 1402 (m), 1373 (w), 1323 (m), 1273 (w), 1234 (m), 1179 (w), 1074 (s), 1040 (s), 1019 (s), 904 (m), 802 (m), 733 (s), 644 (w), 531 (w). |
| **GC-MS** (70 eV) | m/z (%) = 290 (100, [M]$^+$), 275 (33), 259 (79), 241 (12), 217 (45), 188 (28), 173 (29), 158 (11), 143 (19), 128 (42), 91 (25), 65 (6), 41 (20). |
| **HRMS** (ESI) | m/z: 313.1778 u (calc.: m/z = [M+Na]$^+$: 313.1774 u). |

### 3.4.5. (*S*)-3-((1*R*,4*S*)-5,6-Dimethoxy-4,7-dimethyl-1,2,3,4-tetrahydronaphthalen-1-yl)butan-1-ol (15b)

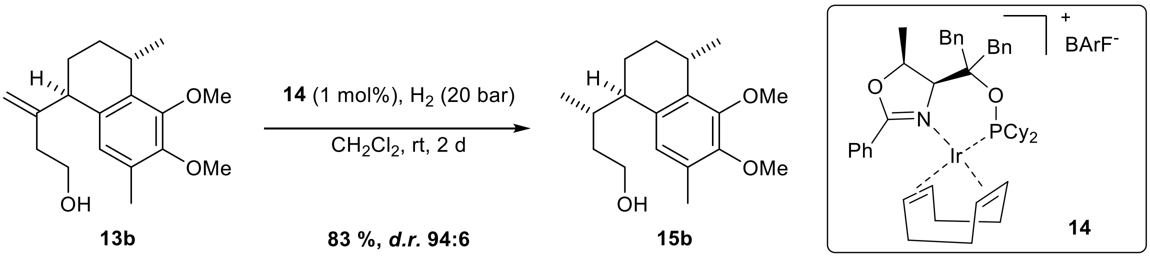


A small glass vessel was charged with 18 mg (0.010 mmol, 1 mol%) of the *Pfaltz* catalyst **14** ([((4*S*,5*S*)-Cy2-Ubaphox)Ir(COD)]BArF), 300 mg (1.033 mmol, 1.0 eq) of olefin **13b** and 11.4 ml of anhydrous and degassed $CH_2Cl_2$. Afterwards, hydrogen gas was bubbled through the solution for 5 min, which resulted in a change of color from orange to yellow. The vessel was transferred into a pressure reactor, which was then flushed with 3 x 20 bar $H_2$ before a pressure of 20 bar was adjusted. The reaction mixture was stirred for 48 h at 20 bar $H_2$ pressure and rt. Afterwards the solvent was removed under reduced pressure. The crude product was purified by filtration over silica gel (CyHex/EtOAc 2:1) to yield 251 mg (0.857 mmol, 83%) of (*S*)-3-((1*R*,4*S*)-configurated alcohol **15b** as a mixture of diastereomers (*d.r.* = 94:6, according to $^1$H NMR) in favor of the desired diastereomer (*S*,*R*,*S*)-**15b**. A chromatographic separation of the diastereomers was not possible at this stage.

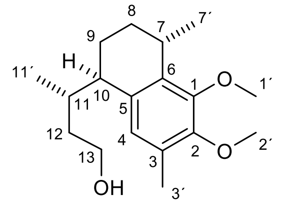


| | |
|---|---|
| **M**($C_{18}H_{28}O$) | 292.42 g/mol. |
| **R$_f$** | 0.30 (CyHex/EtOAc 3:1). |
| **$^1$H NMR** | (500 MHz, $CDCl_3$) δ [ppm] = 6.75 (s, 1 H, H4), 3.88-3.66 (s, 3 H, H1´), 3.18-3.12 (m, 1 H, H7), 3.80 (s, 3 H, H2´), 3.78 (m, 2 H, H13), 2.65-2.60 (m, 1 H, H10), 2.22 (s, 3 H, H3´), 2.16-2.06 (m, 1 H, H11), 1.84-1.47 (m, 6 H, H8, H9, H12), 1.17 (d, *J* = 6.9 Hz, 3 H, H7´), 0.76 (d, *J* = 6.9 Hz, 3 H, H11´). The hydroxyl group could not be detected. |
| **$^{13}$C NMR** | (75 MHz, $CDCl_3$): δ [ppm] = 150.6 (C1), 149.0 (C2), 135.1 (C6), 135.0 (C5), 128.8 (C3), 125.7 (C4), 61.6 (C13), 60.5 (C1´), 59.9 (C2´), 40.2 (C10), 38.7 (C12), 35.5 (C11), 28.0 (C8), 27.3 (C7), 22.4 (C7´), 18.8 (C11), 16.4 (C11´), 15.9 (C3´). |
| **FT-IR** (ATR) | ν [cm$^{-1}$] = 2927 (br, m), 2867 (m), 1480 (m), 1452 (m), 1402 (s), 1377 (w), 1318 (m), 1275 (w), 1232 (m), 1073 (s), 1035 (s), 1010 (m), 913 (m), 870 (w). |
| **GC-MS** (70 eV) | m/z (%) = 292 (10, [M]$^+$), 274 (5), 259 (4), 219 (100), 204 (10), 188 (14), 173 (10), 159 (7), 145 (5), 128 (13), 91 (7), 55 (8). |
| **HRMS** (ESI) | m/z: 315.1934 u (calc.: m/z = [M+Na]$^+$: 315.1931 u). |
| **[α]$_λ^{20}$** | 12.1 ° (436 nm), 15.1 ° (546 nm), 14.9 ° (579 nm), -7.1 ° (589 nm) (0.75 g/100 ml in $CHCl_3$). |

### 3.4.6. (*S*)-3-((1*R*,4*S*)-5,6-Dimethoxy-4,7-dimethyl-1,2,3,4-tetrahydronaphthalen-1-yl)butanal (17)

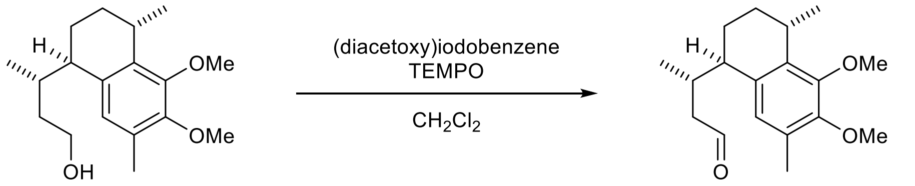


**15b** **91 %** **17**

23 mg (0.145 mmol, 20 mol%) of (2,2,6,6-tetramethylpiperidin-1-yl)oxyl (TEMPO) were dissolved in 5.0 ml of anhydrous $CH_2Cl_2$ before 210 mg (0.723 mmol, 1.0 eq) of alcohol **15b** and 256 mg (0.795 mmol, 1.1 eq) of (diacetoxy)iodobenzene were added consecutively. The solution was stirred for 1 h at rt. The solvent was removed under reduced pressure and the resulting slightly brown residue was dissolved in 15 ml of MTBE before a 1/1-mixture of a saturated $NaHCO_3$ solution and a $Na_2S_2O_3$ solution (10%) were added. The layers were separated, the aqueous layer was extracted with 3 x 15 ml of MTBE and the combined organic layers were dried over $MgSO_4$. After filtration, the solvent was removed under reduced pressure and the crude product was purified by flash column chromatography (CyHex/EtOAc 20:1) to yield 191 mg (0.658 mmol, 91%) of **17** (≥ 95:5 *d.r.* according to NMR and GC) as a viscous oil.

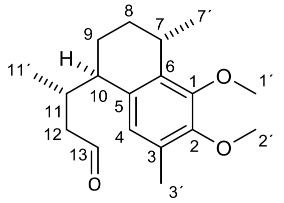

| | |
|---|---|
| **M**($C_{18}H_{26}O_3$) | 290.40 g/mol. |
| **R**$_f$ | 0.29 (CyHex/EtOAc 10:1). |
| $^1$**H NMR** | (600 MHz, $CDCl_3$): δ [ppm] = 9.70 (s, 1 H, H13), 6.72 (s, 1 H, H4), 3.89 (s, 3 H, H1´), 3.81 (s, 3 H, H2´), 3.21-3.10 (m, 1 H, H7), 2.61-2.46 (m, 3 H, H10, H11, H12b), 2.41-2.29 (m, 1 H, H12a), 2.24 (s, 3 H, H3´), 1.95-1.84 (m, 2 H, H8b, H9b), 1.72-1.70 (m, 1 H, H9a), 1.54-1.51 (m, 1 H, H8a), 1.18 (d, *J* = 6.9 Hz, 3 H, C7´), 0.89 (d, *J* = 6.8 Hz, 3 H, H11´). |
| $^{13}$**C NMR** | (150 MHz, $CDCl_3$): δ [ppm] = 202.8 (C13), 150.6 (C1), 149.2 (C2), 134.6 (C6), 133.8 (C5), 128.7 (C3), 125.9 (C4), 60.3 (C1´), 59.7 (C2´), 49.8 (C12), 40.7 (C10), 33.4 (C11), 27.1 (C7), 26.9 (C8), 22.3 (C7´), 19.1 (C9), 17.2 (C11´), 15.7 (C3´). |
| **FT-IR** (ATR) | ν [cm$^{-1}$] = 2932 (s), 2871 (m), 1724 (s), 1478 (s), 1453 (m), 1401 (s), 1371 (w), 1316 (m), 1275 (w), 1234 (m), 1075 (s), 1034 (m), 1011 (m), 955 (w), 914 (w). |
| **GC-MS** (70 eV) | m/z (%) = 290 (6, [M]$^+$), 272 (3), 246 (36), 219 (100), 204 (12), 188 (20), 173 (10), 158 (5), 143 (6), 128 (10), 91 (7), 41 (7). |
| **HRMS** (EI, 70 eV) | m/z: 290.21 u (calc.: m/z = 290.19 u). |
| **[α]**$_λ^{20}$ | -0.7 ° (589 nm) (0.66 g/100 ml in $CHCl_3$). |

### 3.4.7. Diethyl (2-methylallyl)phosphonate (S9)[16]

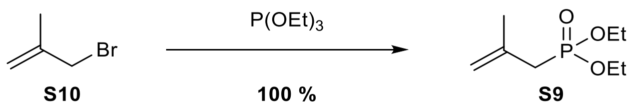

1.0 ml (10 mmol, 1.0 eq) of 3-bromo-2-methylpropene (**S10**) and 1.7 ml (10 mmol, 1.0 eq) of triethyl phosphite were refluxed at 140 °C for 4 h. After cooling to rt, high vacuum was applied to yield 1.92 g (10 mmol, 100%) of **S9**.

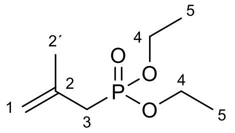

| | |
|---|---|
| **M**($C_8H_{17}O_3P$) | 192.19 g/mol. |
| **R$_f$** | 0.29 (EtOAc). |
| **$^1$H NMR** | (300 MHz, $CDCl_3$): δ [ppm] = 4.92 (dd, *J* = 16.4 Hz, *J* = 5.8 Hz, 2 H, H1), 4.11 (m, 4 H, H4), 2.59 (d, *J* = 22.3 Hz, 2 H, H3), 1.89 (d, *J* = 2.9 Hz, 3 H, H2´), 1.33 (t, *J* = 7.1 Hz, 6 H, H5). |
| **$^{13}$C NMR** | (75 MHz, $CDCl_3$): δ [ppm] = 136.2 (d, *J* = 11.0 Hz, C2), 115.3 (d, *J* = 12.2 Hz, C1), 61.8 (d, *J* = 6.6 Hz, C4), 35.4 (d, *J* = 137.3 Hz, C3), 23.6 (d, *J* = 2.3 Hz, C2´), 16.4 (d, *J* = 6.0 Hz, C5). |
| **$^{31}$P NMR** | (121 MHz, $CDCl_3$): δ [ppm] = 26.99. |
| **FT-IR** (ATR) | ν [cm$^{-1}$] = 3476 (br, m), 3079 (w), 2978 (s), 2931 (m), 2905 (m), 2457 (w), 1790 (w), 1649 (m), 1478 (m), 1443 (m), 1391 (s), 1367 (m), 1284 (s), 1246 (s), 1162 (s), 1096 (s), 1021 (s), 952 (s), 890 (s), 859 (s), 836 (s), 770 (s), 735 (s), 680 (s). |
| **GC-MS** (70 eV) | m/z (%) = 192 (47, [M$^+$]), 177 (9), 163 (17), 152 (22), 136 (100), 125 (41), 109 (28), 97 (95), 82 (73), 67 (10), 55 (81). |
| **HRMS** (EI, 70 eV) | m/z: 192.090 u (calc.: m/z = 192.0915 u). |

The analytical data are in agreement with the literature.[16,17]

### 3.4.8. (1*R*,4*S*)-5,6-Dimethoxy-4,7-dimethyl-1-((*S*,*E*)-6-methylhepta-4,6-dien-2-yl)-1,2,3,4-tetrahydronaphthalene (18)

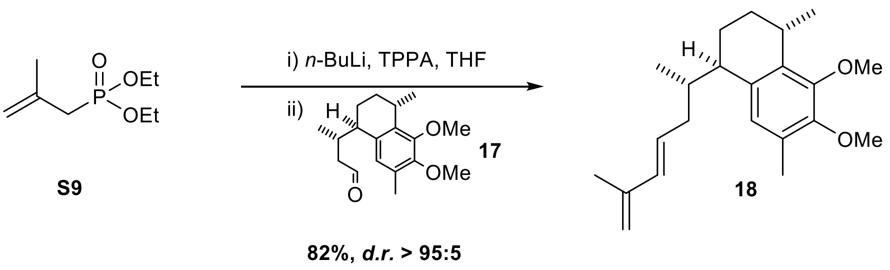

102 mg (0.531 mmol, 1.4 eq) of diethyl (2-methylallyl)phosphonate (**S9**) and 0.17 ml (0.758 mmol, 2.0 eq) of tris(*N*,*N*-tetramethylene)phosphoric acid triamide (TPPA) were dissolved in 1.2 ml of anhydrous THF. The solution was cooled to -78 °C and 0.40 ml (1.6 M in hexane, 0.644 mmol, 1.4 eq) of *n*-BuLi were added over 15 min before a solution of 110 mg (0.379 mmol, 1.0 eq) of aldehyde **17** in

0.8 ml of anhydrous THF were added dropwise. The reaction mixture was stirred for 2 h at -78 °C and another 4 h at rt. Then 3 ml of a saturated $NaHCO_3$ solution and 5 ml of MTBE were added. The layers were separated, the aqueous layer was extracted with 3 x 5 ml of MTBE and the combined organic layers were washed with 3 ml of saturated NaCl solution and dried over $MgSO_4$. After filtration, the solvent was removed under reduced pressure and the crude product was purified by flash column chromatography (CyHex/EtOAc 25:1) to yield 102 mg (0.311 mmol, 82%) of **18** (*d.r.* > 95:5, according to NMR analysis) as a colorless oil.

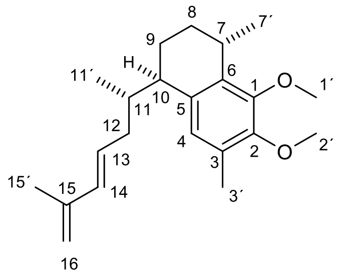


| | |
|---|---|
| **M**($C_{22}H_{32}O_2$) | 328.50 g/mol. |
| **R**$_f$ | 0.44 (CyHex/EtOAc 20:1). |
| **$^1$H NMR** | (500 MHz, $CDCl_3$): δ [ppm] = 6.72 (s, 1 H, H4), 6.17 (d, *J* = 15.2 Hz, 1 H, H14), 5.67 (dt, *J* = 15.2, 7.2 Hz, 1 H, H13), 4.87 (s, 2 H, H16), 3.87 (s, 3 H, H1´), 3.80 (s, 3 H, H2´), 3.14 (Ψdd, *J* = 8.4, 4.5 Hz, 1 H, H7), 2.64 (Ψt, *J* = 6.8 Hz, 1 H, H10), 2.27-2.23 (m, 1 H, H12b), 2.22 (s, 3 H, H3´), 2.13-2.06 (m, 1 H, H12a), 2.04-1.96 (m, 1 H, H11), 1.90-1.77 (m, 2 H, H8b, H9b), 1.84 (s, 3 H, H15´), 1.70-1.64 (m, 1 H, H9a), 1.53-1.46 (m, 1 H, H8a), 1.17 (d, *J* = 6.9 Hz, 3 H, H7´), 0.76 (d, *J* = 6.9 Hz, 3 H, H11´). |
| **$^{13}$C NMR** | (125 MHz, $CDCl_3$): δ [ppm] = 150.6 (C1), 149.0 (C2), 142.3 (C15), 135.4 (C6), 135.0 (C5), 134.1 (C14), 130.2 (C13), 128.7 (C3), 125.8 (C4), 114.4 (C16), 60.5 (C1´), 60.0 (C2´), 39.8 (C10), 39.6 (C11), 39.2 (C12), 27.9 (C8), 27.4 (C7), 22.4 (C7´), 18.9 (C15´), 18.7 (C9), 16.5 (C11´),16.0 (C3´). |
| **FT-IR** (ATR) | ν [$cm^{-1}$] = 2925 (s), 2870 (m), 1603 (w), 1481 (s), 1453 (m), 1402 (s), 1318 (m), 1272 (w), 1237 (m), 1076 (s), 964 (m), 916 (w), 878 (m). |
| **GC-MS** (70 eV) | m/z (%) = 328 (21, $[M]^+$), 313 (10), 297 (2), 272 (10), 246 (31), 219 (100), 204 (10), 189 (21), 173 (13), 158 (7), 143 (7), 128 (11), 105 (5), 79 (10), 41 (12). |
| **HRMS** (EI, 70 eV) | m/z: 328.23 u (calc.: m/z = 328.24 u). |
| **$[\alpha]_\lambda^{20}$** | -13.6 ° (589 nm) (1.35 g/100 ml in $CHCl_3$). |

### 3.4.9. (1*R*,3*S*,3a*R*,6*S*)-7,8-Dimethoxy-3,6,9-trimethyl-1-(2-methylprop-1-en-1-yl)-2,3,3a,4,5,6-hexahydro-1H-phenalene (19)

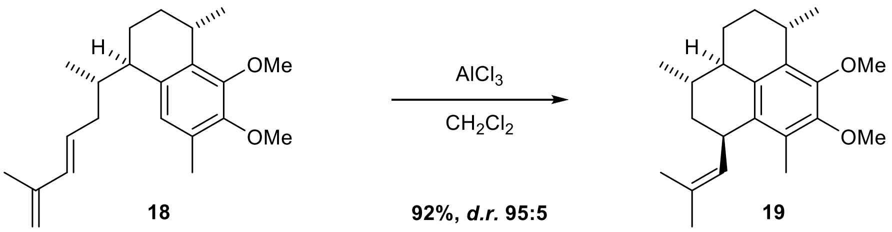


**18** **92%, *d.r.* 95:5** **19**

To 5.8 mg (0.043 mmol, 20 mol%) of $AlCl_3$ were added 20 ml of anhydrous $CH_2Cl_2$. The stirred reaction mixture was cooled to -5 °C before a solution of 71.0 mg (0.216 mmol, 1.0 eq) of diene **18** in 2 ml of anhydrous $CH_2Cl_2$ were added dropwise over 30 min. Stirring was continued for 4 h before 10 ml of a saturated $NaHCO_3$ solution were added. The layers were separated, the aqueous layer was extracted with 3 x 15 ml of $CH_2Cl_2$ and the combined organic layers were dried over $MgSO_4$. After filtration, the solvent was removed under reduced pressure and the crude product was purified by flash column chromatography (CyHex/EtOAc 40:1) to yield 65.5 mg (0.199 mmol, 92%) as a diastereomeric mixture (*d.r.* = 95:5 at C13, according to characteristic signals in the $^1$H NMR) in favor of the desired diastereomer **19**. A chromatographic separation of the diastereomers was not possible at this stage.

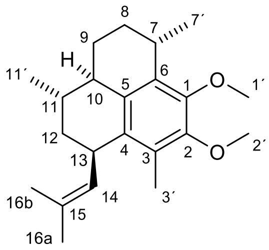


| | |
|---|---|
| **M**($C_{22}H_{32}O_2$) | 328.50 g/mol. |
| **R$_f$** | 0.45 (CyHex/EtOAc 20:1). |
| **$^1$H NMR** | (500 MHz, $CDCl_3$): δ [ppm] = 1.05 (d, *J* = 6.1 Hz, 3 H, H11´), 1,17-1.08 (m, 1 H, H9a), 1.22 (d, *J* = 7.1 Hz, 3 H, H7´), 1.66-1.50 (m, 4 H, H8a/H11/H12), 1.69 (s, 3 H, H16b), 1.76 (s, 3 H, H16a), 2.06-1.99 (m, 1 H, H8b), 2.07 (s, 3 H, H3´), 2.14-2.09 (m, 1 H, H10), 2.23-2.17 (m, 1 H, H9b), 3.44-3.35 (m, 1 H, H7), 3.63-3.57 (m, 1 H, H13), 3.79 (s, 3 H, H2´), 3.86 (s, 3 H, H1´), 5.14 (d, *J* = 9.2 Hz, 1 H, H14). |
| **$^{13}$C NMR** | (125 MHz, $CDCl_3$): δ [ppm] = 11.0 (C3´), 17.8 (C16a), 21.2 (C11´), 23.6 (C7´), 25.8 (C16b), 27.2 (C7), 27.8 (C9), 29.8 (C8), 30.5 (C11), 35.8 (C13), 39.5 (C12), 42.3 (C10), 60.2 (C1´), 60.7 (C2´), 128.0 (C4), 129.8 (C14), 130.1 (C15), 133.5 (C5), 133.8 (C3), 134.1 (C6), 148.7 (C1), 149.2 (C2). |
| **FT-IR** (ATR) | ν [cm$^{-1}$] = 2921 (m), 2866 (w), 1458 (m), 1412 (m), 1374 (w), 1313 (s), 1250 (w), 1133 (w), 1067 (s), 1044 (m), 1011 (w), 940 (w), 844 (w), 798 (w). |
| **GC-MS** (70 eV) | m/z (%) = 328 (99, [M]$^+$), 313 (100), 297 (10), 272 (20), 257 (31), 229 (17), 214 (5), 199 (11), 183 (7), 165 (9), 143 (56), 128 (9), 56 (5), 41 (8). |
| **HRMS** (ESI) | m/z: 351.2299 u (calc.: m/z = 351.2295 u). |
| **[α]$_λ^{20}$** | -111.5 ° (589 nm) (2.42 g/100 ml in $CHCl_3$). |

The analytical data are in agreement with the literature.[3,18–20]

### 3.4.10. Pseudopterosin A-F aglycone (20)[18,19]

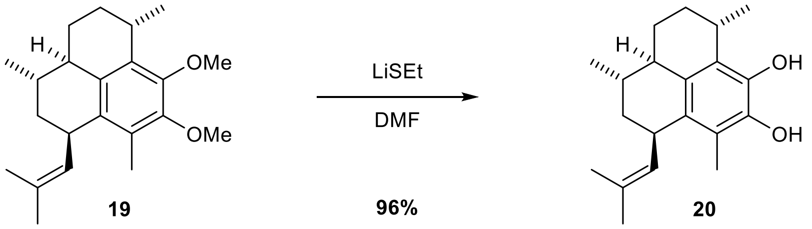


0.581 ml (1.31 M in hexane, 0.761 mmol, 10.0 eq) of *n*-BuLi were diluted with 1.0 ml of anhydrous hexane. The solution was cooled to 0 °C and 0.068 ml (0.913 mmol, 12.0 eq) ethanethiol were added dropwise. The slurry was stirred for 10 min at 0 °C and afterwards for 1 h at rt. The solvent was removed under reduced pressure and the resulting white residue was dried for further 30 min (line vacuum). Then a solution of 25.0 mg (0.076 mmol, 1.0 eq) of compound **19** in 1.25 ml of anhydrous DMF were added dropwise before refluxing the reaction mixture for 3 h. The solution was allowed to cool to rt and after addition of 10 ml of MTBE and 5 ml of a 2 M HCl solution the layers were separated. The aqueous layer was extracted with 3 x 10 ml of MTBE and the combined organic layers were dried over $Na_2SO_4$. After filtration, the solvent was removed under reduced pressure to yield 21.9 mg (0.073 mmol, 96%) of pseudopterosin A-F aglycone (**20**) as colorless oil.

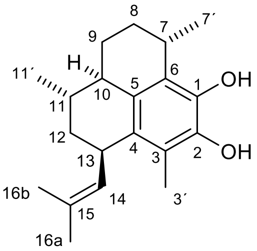


| | |
|---|---|
| **M**($C_{20}H_{28}O_2$) | 300.44 g/mol. |
| **R$_f$** | 0.43 (CyHex/EtOAc 5:1). |
| **$^1$H NMR** | (600 MHz, $CDCl_3$): δ [ppm] = 5.11 (d, *J* = 9.2 Hz, 1 H, H14), 5.04 (s, br, 1 H, O*H*), 4.84 (s, br, 1 H, O*H*), 3.61-3.56 (m, 1 H, H13), 3.22 (h, *J* = 6.9 Hz, 1 H, H7), 2.21-2.12 (m, 2 H, H8b/H9b), 2.06-1.99 (m, 1 H, H10), 2.03 (s, 3 H, H3´),1.75 (s, 3 H, H16a), 1.70-1.63 (m, 2 H, H12), 1.67 (s, 3 H, H16b), 1.57-1.55 (m, 1 H, H11), 1.50-1.44 (m, 1 H, H8a), 1.25 (d, *J* = 7.0 Hz, 3 H, H7´), 1.12-1.06 (m, 1 H, H9a), 1.04 (d, *J* = 6.3 Hz, 3 H, H11´). |
| **$^{13}$C NMR** | (150 MHz, $CDCl_3$): δ [ppm] = 10.8 (C3´), 17.7 (C16a), 21.0 (C11´), 23.1 (C7´), 25.7 (C16b), 27.3 (C7), 28.2 (C9), 29.9 (C11), 31.0 (C8), 35.3 (C13), 39.5 (C12), 43.2 (C10), 119.7 (C4), 125.8 (C6), 129.6 (C15), 129.8 (C5), 130.1 (C3), 130.2 (C14), 139.6 (C1), 139.8 (C2). |
| **FT-IR** (ATR) | ν [cm$^{-1}$] = 2924 (s), 2861 (m), 1460 (m), 1447 (m), 1376 (w), 1308 (m), 1290 (m), 1275 (m), 1189 (w), 1110 (w), 1047 (w), 1034 (m), 841 (w), 818 (w). |
| **GC-MS** (70 eV) | m/z (%) = 300 (73, [M]$^+$), 285 (100), 267 (10), 244 (23), 229 (33), 201 (33), 185 (9), 165 (12), 143 (6), 128 (13), 91 (8), 56 (9), 41 (16). |

| | |
|---|---|
| **HRMS** (ESI) | The values obtained correspond to the oxidized form of **3** (*ortho*-quinone). m/z: 321.1819 u (calc.: m/z = $[M+Na]^+$: 321.1825 u). |

The analytical data are in agreement with the literature.[18,21,22]

## 3.5. Synthesis of *iso*-pseudopterosin A (*iso*-3)

### 3.5.1. (3*R*,4*S*,5*R*)-Tetrahydro-2*H*-pyran-2,3,4,5-tetrayl tetraacetate (S11)[23]

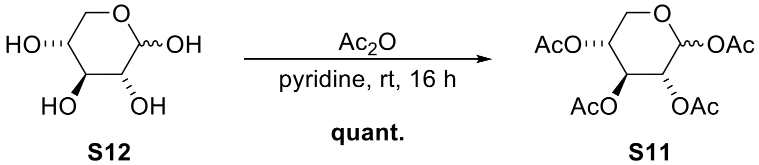


Under argon atmosphere, 500 mg (3.33 mmol, 1.0 eq) of D-xylose (**S12**) were dissolved in 3 ml of abs. pyridine. Then 5 ml (53 mmol, 16 equiv) of acetic anhydride were added dropwise. After complete conversion (TLC), the reaction was quenched by adding 10 ml of ethanol and the solvent was evaporated under reduced pressure. Remaining residues of pyridine were removed by azeotropic distillation with 20 ml of toluene (4 times) to yield 1.057 g (3.32 mmol, 100 %) of the desired tetraacetate **S11** as a colourless syrup in an anomeric mixture of *α*:*β* = 2:1.

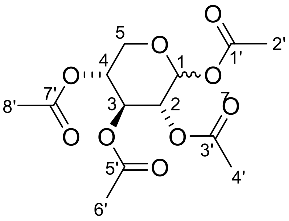

| | |
|---|---|
| **M**($C_{13}H_{18}O_9$) | 318.28 g/mol. |
| **R**f | 0.30 (CyHex/EtOAc 2:1). |
| **$^1$H NMR** | (400 MHz, $CDCl_3$): δ [ppm] = (***α***) = 6.26 (d, *J* = 3.6 Hz, 1 H, H1); 5.47 (t, *J* = 9.9 Hz, 1 H, H4); 5.07-4.95 (m, 2 H, H2/H3); 3.94 (dd, *J* = 5.9 Hz, *J* = 11.2 Hz, 1 H, H5a); 3.72 (t, *J* = 11.2 Hz, 1 H, H5b); 2.18 (s, 3 H, H2‘); 2.07-2.05 (m, 6 H, H4‘/H6‘/H7‘); 2.03 (s, 3 H, H4‘/H6‘/H7‘). |
| | (***β***) = 5.73 (d, $^3J$ = 6.9 Hz, 1 H, H1); 5.21 (t, *J* = 8.3 Hz, 1 H, H4); 5.07-4.95 (m, 2 H, H2/H3); 4.15 (dd, *J* = 12.0, 5.0 Hz, 1 H, H5a); 3.54 (dd, *J* = 12.0 Hz, 8.4 Hz, 1 H, H5b); 2.11 (s, 3 H, H2‘); 2.07-2.05 (m, 9 H, H4‘/H6‘/H7‘). |
| **$^{13}$C NMR** | (125 MHz, $CDCl_3$): δ [ppm] = (***α***) = 170.1 (C1’ or C3’ or C5’ or C7’); 169.7 (C1’ or C3’ or C5’ or C7’); 169.7 (C1’ or C3’ or C5’ or C7’); 169.0 (C1’ or C3’ or C5’ or C7’); 89.2 (d, C1); 69.3 (d, C2 or C3 or C4); 69.3 (d, C2 or C3 or C4); 68.6 (d, C2 or C3 or C4); 60.6 (t, C5); 20.8 (q, C2’ or C4’ or C6’ or C8’); 20.7 (q, C2’ or C4’ or C6’ or C8’); 20.6 (q, C2’ or C4’ or C6’ or C8’); 20.5 (q, C2’ or C4’ or C6’ or C8’). |
| | (***β***) = 169.8 (s, C1’ or C3’ or C5’ or C7’); 169.8 (s, C1’ or C3’ or C5’ or C7’); 169.3 (s, C1’ or C3’ or C5’ or C7’); 169.0 (s, C1’ or C3’ or C5’ or C7’); 92.0 (d, |

C1); 70.9 (d, C4); 69.4 (d, C2 or C3); 68.3 (d, C2 or C3); 62.7 (t, C5); 20.8 (q, 1C, C2' or C4' or C6' or C8'), 20.6 (q, 1C, C2' or C4' or C6' or C8').

**FT-IR** (ATR) ν [cm$^{-1}$] = 1744 (s); 1433 (w); 1368 (m); 1207 (s); 1127 (w); 1078 (m); 1039 (s); 1013 (s); 935 (m); 877 (w); 732 (w); 647 (w); 601 (w); 486 (w); 471 (w).

**HRMS** (ESI) m/z: 341.0846 u (calc.: m/z = $[M+Na]^+$: 341.0843 u).

The analytical data are in agreement with the literature.[24]

### 3.5.2. (3*R*,4*S*,5*R*)-2-Hydroxytetrahydro-2*H*-pyran-3,4,5-triyl triacetate (S13)[25,26]

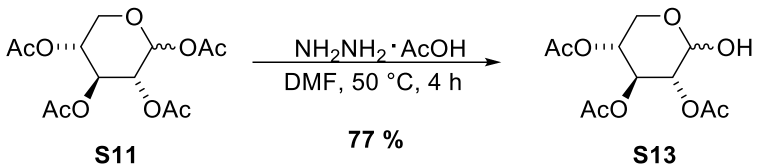


**S11** **S13**

Under argon atmosphere, 1.00 g (3.14 mmol, 1.0 eq) of tetraacetyl-D-xylose (**S11**) and 376 mg (4.09 mmol, 1.3 eq) hydrazine acetate were dissolved in 4 ml of dry DMF and stirred at 50 °C for 4 h. After complete conversion (TLC), 10 ml of water were added and the aqueous layer was extracted three times with ethyl acetate. The combined organic layers were dried over $Na_2SO_4$ and the solvent was evaporated under reduced pressure. The residue was subjected to column chromatography (CyHex/EtOAc 4:1) to obtain 668 mg (2.42 mmol, 77 %) of the triacetate **S13** as a colourless solid.

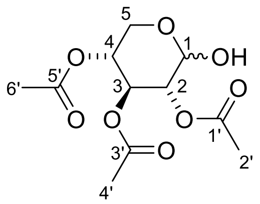


**M**($C_{11}H_{16}O_8$) 276.24 g/mol.

**R$_f$** 0.13 (CyHex/EtOAc 2:1).

**$^1$H NMR** (400 MHz, $CDCl_3$): δ [ppm] = 5.51 (t, *J* = 9.6 Hz, 1 H, H3); 5.38 (br t, *J* = 3.4 Hz, 1 H, H1); 4.96 (ddd, *J* = 10.2 Hz, 9.9 Hz, 6.0 Hz, 1 H, H4); 4.83 (dd, *J* = 9.9 Hz, 3.4 Hz, 1 H, H2); 4.20 (br, 1 H, OH); 3.88 (t, *J* = 11.0 Hz, 1 H, H5a); 3.81 (dd, *J* = 11.0 Hz, 6.0 Hz, 1 H, H5b); 2.09 (s, 3 H, H2'/H4'/H6'); 2.05 (s, 3 H, H2'/H4'/H6'); 2.05 (s, 3 H, H2'/H4'/H6').

**$^{13}$C NMR** (125 MHz, $CDCl_3$): δ [ppm] = 170.5 (C1' or C3' or C5'); 170.3 (s, C1' or C3' or C5'); 170.3 (C1' or C3' or C5'); 90.1 (d, C1); 71.3 (d, C2); 69.4 (d, C3 or C4); 69.3 (d, C3 or C4); 58.3 (t, C5); 20.8 (q, C2' or C4' or C6'); 20.8 (q, 2C, C2' or C4' or C6').

**FT-IR** (ATR) ν [cm$^{-1}$] = 3465 (br); 2961 (w); 1742 (s); 1433 (w); 1368 (m); 1219 (s); 1141 (w); 1049 (s); 940 (w); 909 (w); 756 (w); 603 (w); 486 (w); 461 (w).

**HRMS** (ESI) m/z: 299.0740 u (calc.: m/z = $[M+Na]^+$: 299.0737 u).

The analytical data are in agreement with the literature.[27]

### 3.5.3. (2*R*,3*R*,4*S*,5*R*)-2-(2,2,2-Trichloro-1-iminoethoxy)tetrahydro-2*H*-pyran-3,4,5-triyl triacetate (21)[26]

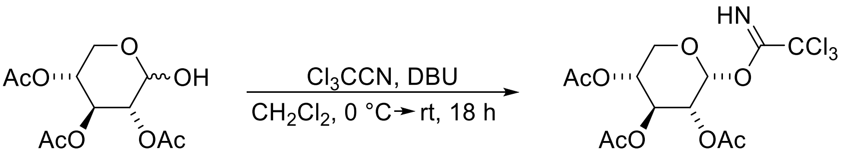


**S13** **81 %** **21**

660 mg (2.39 mmol, 1.0 eq) of 2,3,4-triacetyl-D-xylose (**S13**) were dissolved in 15 ml of dry dichloromethane under argon atmosphere and cooled to 0 °C. Afterwards, first 71 µl (0.48 mmol, 0.2 eq) of DBU and then 0.28 ml (2.80 mmol, 1.7 eq) of trichloroacetonitrile were added dropwise. The reaction mixture was stirred for 3 h at 0 °C and overnight at room temperature. After complete conversion, the solution was diluted with further 15 ml of dichloromethane and washed two times with an aqueous saturated NaCl solution. The organic layer was dried over $Na_2SO_4$ and the solvent was removed under reduced pressure. After column chromatographic purification (CyHex/EtOAc 4:1) 814 mg (1.93 mmol, 81 %) of the desired trichloroacetimidate **21** were obtained as a yellowish syrup (single diastereomer).

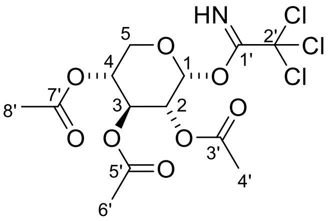

| | |
|---|---|
| **M**($C_{13}H_{16}Cl_3NO_8$) | 420.62 g/mol. |
| **R**$_f$ | 0.43 (CyHex/EtOAc 2:1). |
| **$^1$H NMR** | (400 MHz, $CDCl_3$): δ [ppm] = 8.70 (s, 1 H, NH); 6.49 (d, *J* = 3.6 Hz, 1 H, H1); 5.57 (t, *J* = 9.9 Hz, 1 H, H3); 5.12-5.05 (m, 2 H, H2/H4); 3.99 (dd, *J* = 11.2 Hz, 5.9 Hz, 1 H, H5a); 3.82 (t, *J* = 11.2 Hz, 1 H, H5b); 2.07 (s, 3 H, H4'/H6'/8'); 2.06 (s, 3 H, H4'/H6'/8'); 2.03 (s, 3 H, H4'/H6'/8'). |
| **$^{13}$C NMR** | (125 MHz, $CDCl_3$): δ [ppm] = 169.9 (C3' or C5' or C7'); 169.9 (C3' or C5' or C7'); 169.9 (C3' or C5' or C7'); 160.9 (C1'); 93.2 (C1); 90.8 (C2'); 69.9 (d, C2 or C4); 69.3 (d, C3); 68,6 (d, C2 or C4); 60.8 (t, C5); 20.8 (q, C4' or C6' or C8'); 20.7 (q, C4' or C6' or C8'); 20.5 (q, C4' or C6' or C8'). |
| **FT-IR** (ATR) | ν [cm$^{-1}$] = 3318 (br, w); 1753 (s); 1677 (w); 1369 (w); 1293 (w); 1219 (s); 1042 (m); 971 (w); 922 (w); 834 (w); 797 (w); 645 (w); 486 (w). |
| **HRMS** (ESI) | m/z: 441.9839 u (calc.: m/z = [M+Na]$^+$: 441.9834 u). |

The analytical data are in agreement with the literature.[28,29]

### 3.5.4. Synthesis of *iso*-pseudoptersoin A (iso-3) [30]

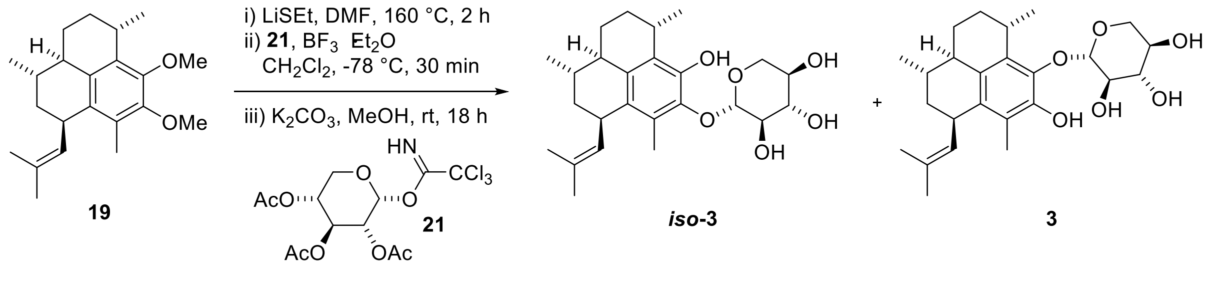


Under argon atmosphere, 0.033 ml (0.444 mmol, 12 eq) of ethanethiol were added to a solution of 0.2 ml (0.370 mmol, 10 eq, 1.6 M in hexanes) of *n*-BuLi in 0.5 ml of absolute *n*-hexane at 0 °C. After stirring for 10 min at 0 °C, the mixture was stirred for 30 min at r.t. before the solvent was removed under reduced pressure. To the colorless residue was added a solution of 12.0 mg (0.037 mmol, 1.0 eq) of **19** in 0.2 ml of DMF. Then, the reaction mixture was refluxed for 2 h. After cooling to r.t., 2 ml of aqueous 2 M HCl were added. The layers were separated and the aqueous layer was extracted three times with ethyl acetate. The combined organic layers were dried over $Na_2SO_4$, filtrated and the solvent was removed under reduced pressure. To remove traces of water the resulting crude product (**20**) was dissolved in 0.3 ml of dry toluene under argon and the solvent was removed in vacuo (oil pump). Afterwards, a solution of 20.0 mg (0.048 mmol, 1.3 Äq.) of trichloroacetimidate **21** in 0.3 ml of toluene was added and the solvent was again removed under reduced pressure. Then, 48 mg of powdered and activated molecular sieves (4 Å) and 0.5 ml of dry dichloromethane were added under argon. The suspension was stirred for 10 min, cooled to -78 °C and two drops of $BF_3$•$Et_2O$ were added. After stirring for 30 min at -78 °C, the reaction mixture was quenched with a few drops of a saturated aqueous $NaHCO_3$ solution and allowed to warm to r.t.. The suspension was filtrated over a 1:1 mixture of celite and $Na_2SO_4$ with dichloromethane. The solvent was removed under reduced pressure and the residue was dissolved in 0.5 ml MeOH and 0.3 mg (0.002 mmol, 5 mol%) of $K_2CO_3$ were added. After stirring overnight, the solvent was removed under reduced pressure and the crude product was purified by column chromatography ($CHCl_3$/MeOH 30:1) to yield 8.0 mg (0.019 mmol, 50 % over three steps) of a colorless solid. According to HPLC analysis and NMR (using a known mixture of pseudopterosin A-D as a reference) a 85:15 mixture of the product *iso*-pseudopterosin A (*iso*-**3**) and its regioisomer pseudopterosin A (**3**) was formed.

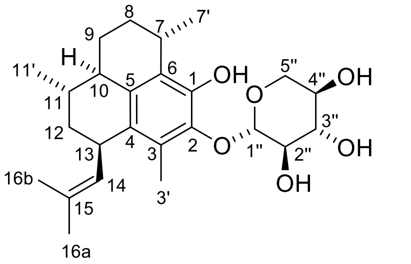

**M**($C_{25}H_{36}O_6$) 432.56 g/mol.

**$R_f$** 0.1 ($CHCl_3$/MeOH 20:1).

| | |
|---|---|
| **$^1$H NMR** | (400 MHz, $CD_3OD$): δ [ppm] = 5.04 (d, *J* = 9.0 Hz, 1 H, H14), 4.36 (d, *J* = 7.8 Hz, 1 H, H1"), 3.88 (dd, *J* = 11.4, 5.4 Hz, 1 H, H5"a), 3.60-3.52 (m, 2 H, H13/H4"), 3.49-3.45 (m, 1 H, H2"), 3.37-3.34 (m, 1 H, H3"), 3.32-3.27 (m, 1 H, H7), 3.13 (t, *J* = 11.4 Hz, 1 H, H5"b), 2.17-2.07 (m, 2 H, H8a/H9a), 2.09 (s, 3 H, H3'), 2.00-1.94 (m, 1 H, H10), 1.73 (s, 3 H, H16a), 1.68-1.55 (m, 2 H, H12), 1.66 (s, 3 H, H16b), 1.54-1.49 (m, 1 H, H11), 1.48-1.38 (m, 1 H, H8b), 1.16 (d, *J* = 7.0 Hz, 3 H, H7'), 1.05-0.98 (m, 1 H, H9b), 1.02 (d, *J* = 6.1 Hz, 3 H, H11'). |
| **$^{13}$C NMR** | (125 MHz, $CD_3OD$): δ [ppm] = 146.1 (C1), 143.3 (C2), 135.7 (C4/5), 131.6 (d, C14), 130.7 (C4/5), 129.9 (C15), 128.2 (C3), 128.1 (C6), 108.3 (d, C1"), 78.0 (d, C3"), 75.4 (d, C2"), 71.0 (d, C4"), 67.4 (t, C5"), 44.8 (d, C10), 40.8 (t, C12), 36.6 (d, C13), 32.0 (t, C8), 31.2 (d, C11), 29.6 (t, C9), 28.5 (d, C7), 25.9 (q, C16b), 23.5 (q, C7'), 21.4 (q, C11'), 17.8 (q, C16a), 12.2 (q, C3'). |
| **FT-IR** (ATR) | ν [$cm^{-1}$] = 3342 (br), 2923 (m), 2864 (m), 1642 (w), 1597 (w), 1455 (m), 1430 (m), 1375 (m), 1313 (m), 1284 (m), 1260 (w), 1233 (w), 1040 (s), 1021 (s). |
| **HRMS** (ESI) | m/z: 455.24062 u (calc.: m/z = $[M+Na]^+$: 455.24041 u). |
| **$[\alpha]_\lambda^{20}$** | -91.15 ° (436 nm); -44.59 ° (546 nm); -37,38 ° (579 nm); -41,31 ° (589 nm) (0.15 g/100 ml in $CHCl_3$). |

**References for section 3**


[1] S. Werle, T. Fey, J. M. Neudörfl, H.-G. Schmalz, *Org. Lett.* **2007**, *9*, 3555–3558.

[2] T. S. Kaufman, *J. Chem. Soc., Perkin Trans. 1* **1993**, 403–404.

[3] D. J. Mans, G. A. Cox, T. V. Rajanbabu, *J. Am. Chem. Soc.* **2011**, *133*, 5776–5779.

[4] H. Kiuchi, D. Takahashi, K. Funaki, T. Sato, S. Oi, *Org. Lett.* **2012**, *14*, 4502–4505.

[5] T. Hayashi, M. Konishi, K. I. Yokota, M. Kumada, *J. Organomet. Chem.* **1985**, *285*, 359–373.

[6] J. D. White, P. R. Blakemore, N. J. Green, E. B. Hauser, M. A. Holoboski, L. E. Keown, C. S. Nylund Kolz, B. W. Phillips, *J. Org. Chem.* **2002**, *67*, 7750–7760.

[7] W. Lölsberg, S. Werle, J.-M. Neudörfl, H.-G. Schmalz, *Org. Lett.* **2012**, *14*, 5996–5999.

[8] G. Wang, E. I. Negishi, *Eur. J. Org. Chem.* **2009**, 1679–1682.

[9] Y. Xiao, P. Liu, *Angew. Chem. Int. Ed.* **2008**, *47*, 9722–9725.

[10] G. A. Kraus, I. Jeon, *Org. Lett.* **2006**, *8*, 5315–5316.

[11] R. R. Cesati, J. de Armas, A. H. Hoveyda, *J. Am. Chem. Soc.* **2004**, *126*, 96–101.

[12] A. I. Meyers, D. Stoianova, *J. Org. Chem.* **1997**, *3263*, 5219–5221.

[13] T. G. Elford, S. Nave, R. P. Sonawane, V. K. Aggarwal, *J. Am. Chem. Soc.* **2011**, *133*, 16798–16801.

[14] J. S. Yadav, A. K. Basak, P. Srihari, *Tetrahedron Lett.* **2007**, *48*, 2841–2843.

[15] A. D. Rodríguez, C. Ramírez, *J. Nat. Prod.* **2001**, *64*, 100–102.

[16] M. F. Probst, A. M. Modro, T. A. Modro, *Can. J. Chem.* **1997**, *75*, 1131–1135.

[17] K. Nishikawa, H. Nakahara, Y. Shirokura, Y. Nogata, E. Yoshimura, T. Umezawa, T. Okino, F. Matsuda, *Org. Lett.* **2010**, *12*, 904–907.

[18] J. P. Cooksey, P. J. Kocieński, A. W. Schmidt, T. N. Snaddon, C. A. Kilner, *Synthesis* **2012**, *44*, 2779–2785.

[19] A. Majdalani, H.-G. Schmalz, *Synlett* **1997**, *1997*, 1303–1305.

[20] P. J. Kocienski, A. Pontiroli, L. Qun, *J. Chem. Soc. Perkin Trans. 1* **2001**, 2356–2366.

[21] A. Majdalani, H.-G. Schmalz, **1997**, *38*, 4545–4548.

[22] E. J. Corey, S. E. Lazerwith, *J. Am. Chem. Soc.* **1998**, *120*, 12777–12782.

[23] D. E. Long, P. Karmakar, K. A. Wall, S. J. Sucheck, *Bioorganic Med. Chem.* **2014**, *22*, 5279–5289.

[24] X. Wei, Y. Ma, Q. Wu, J. Zhang, Z. Cai, M. Lu, V. Ferro, *Molecules* **2015**, *20*, 21681–21699.

[25] M. Mori, Y. Ito, T. Ogawa, *Carbohydr. Res.* **1990**, *195*, 199–224.

[26] Y. Nishi, N. Yamane, T. Tanimoto, *Carbohydr. Res.* **2007**, *342*, 2173–2181.

[27] B. Beagley, D. S. Larsen, R. G. Pritchard, R. J. Stoodley, *J. Chem. Soc. Perkin Trans. 1* **1990**, 3113–3127.

[28] N. Konishi, T. Shirahata, M. Yokoyama, T. Katsumi, Y. Ito, N. Hirata, T. Nishino, K. Makino, N. Sato, T. Nagai, et al., *J. Org. Chem.* **2017**, *82*, 6703–6719.

[29] H. Liu, Y. Zhang, R. Wei, G. Andolina, X. Li, *J. Am. Chem. Soc.* **2017**, *139*, 13420–13428.

[30] J. M. Pletcher, F. E. McDonald, *Org. Lett.* **2005**, *7*, 4749–4752.

# 4. $^1$H and $^{13}$C NMR Spectra

$^1$H and $^{13}$C NMR spectra of compound **S1**

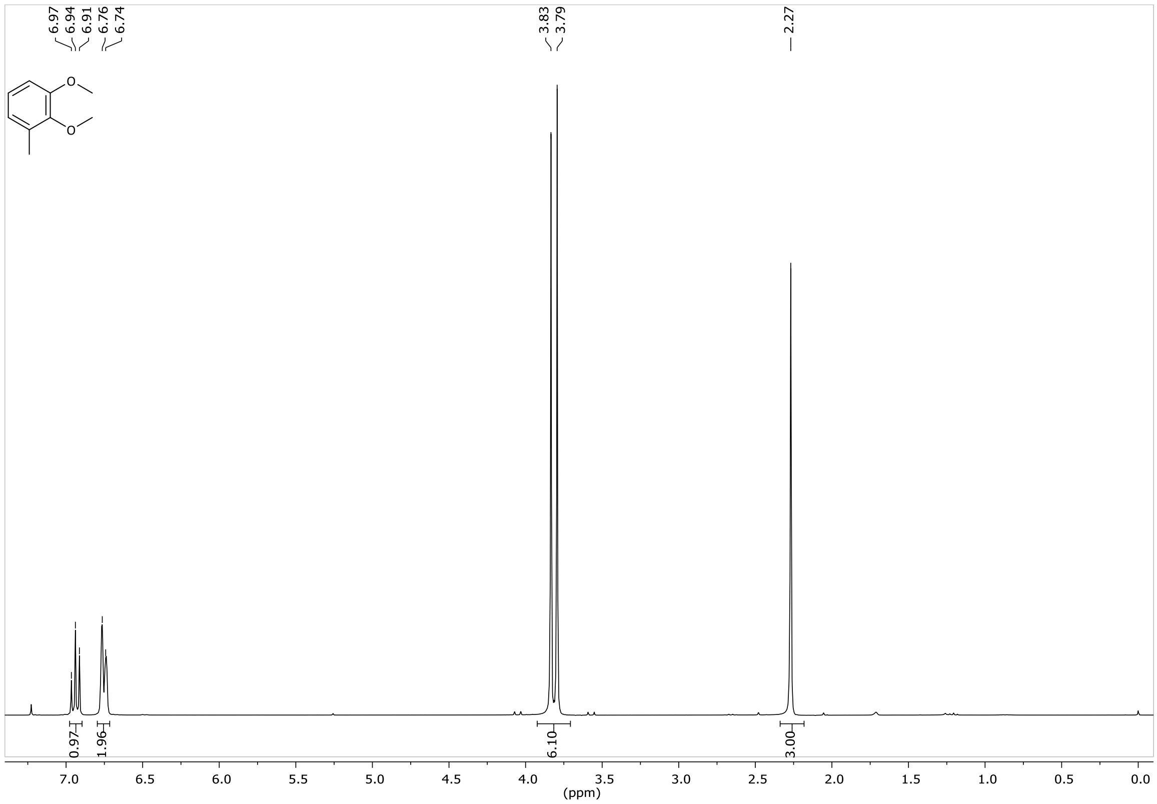


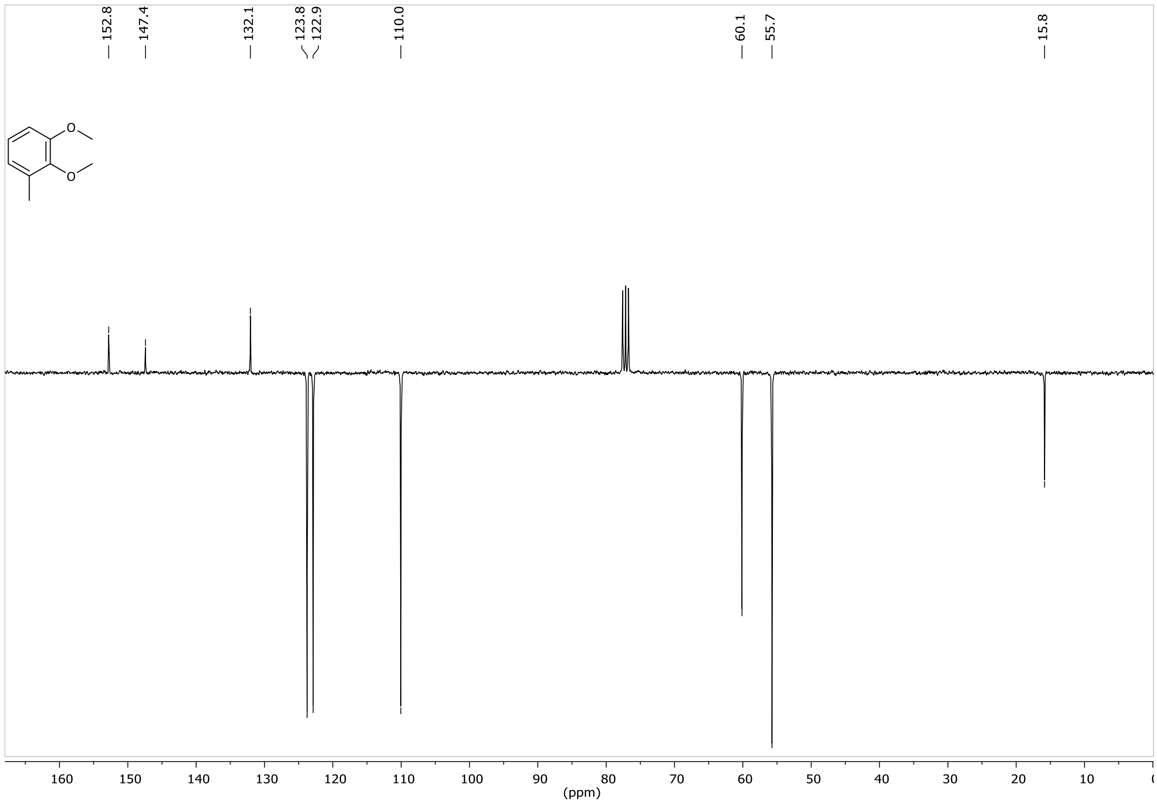

$^1H$ and $^{13}C$ NMR spectra of compound **S3**

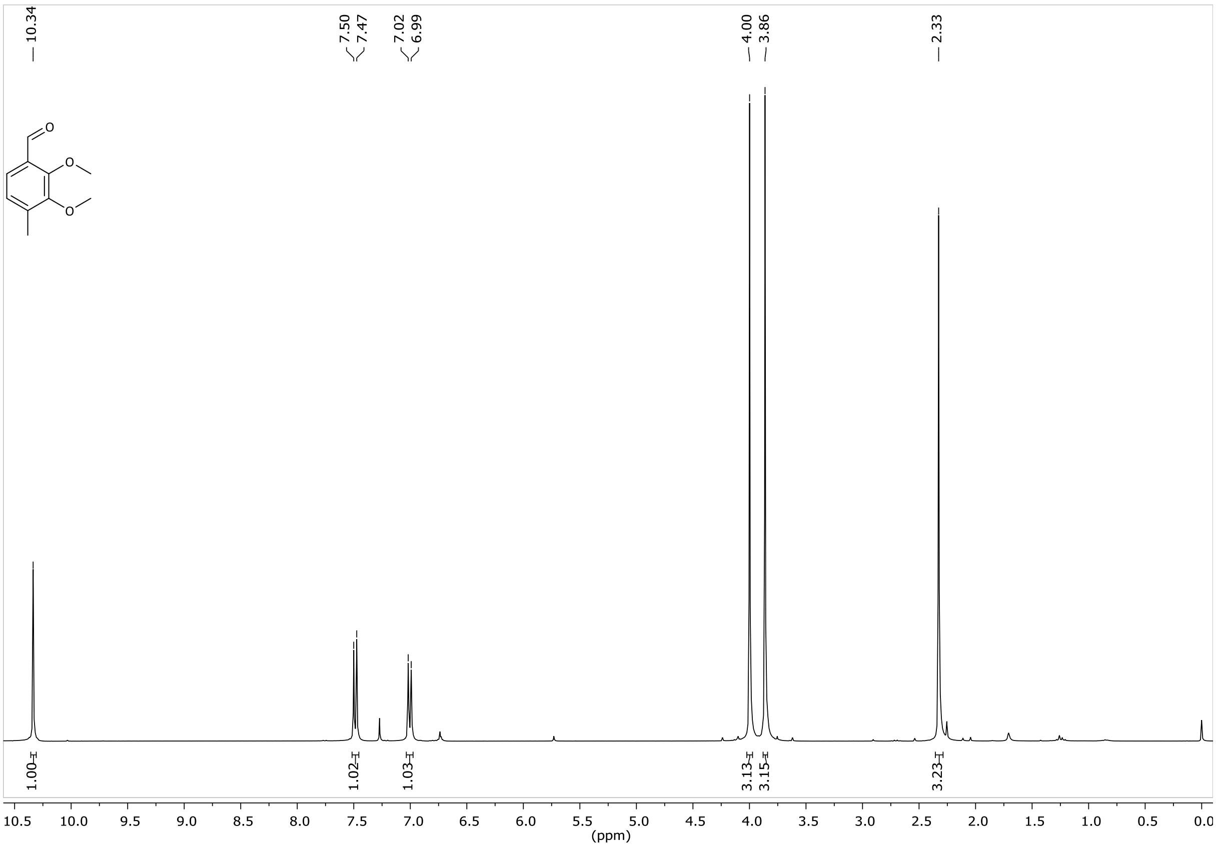


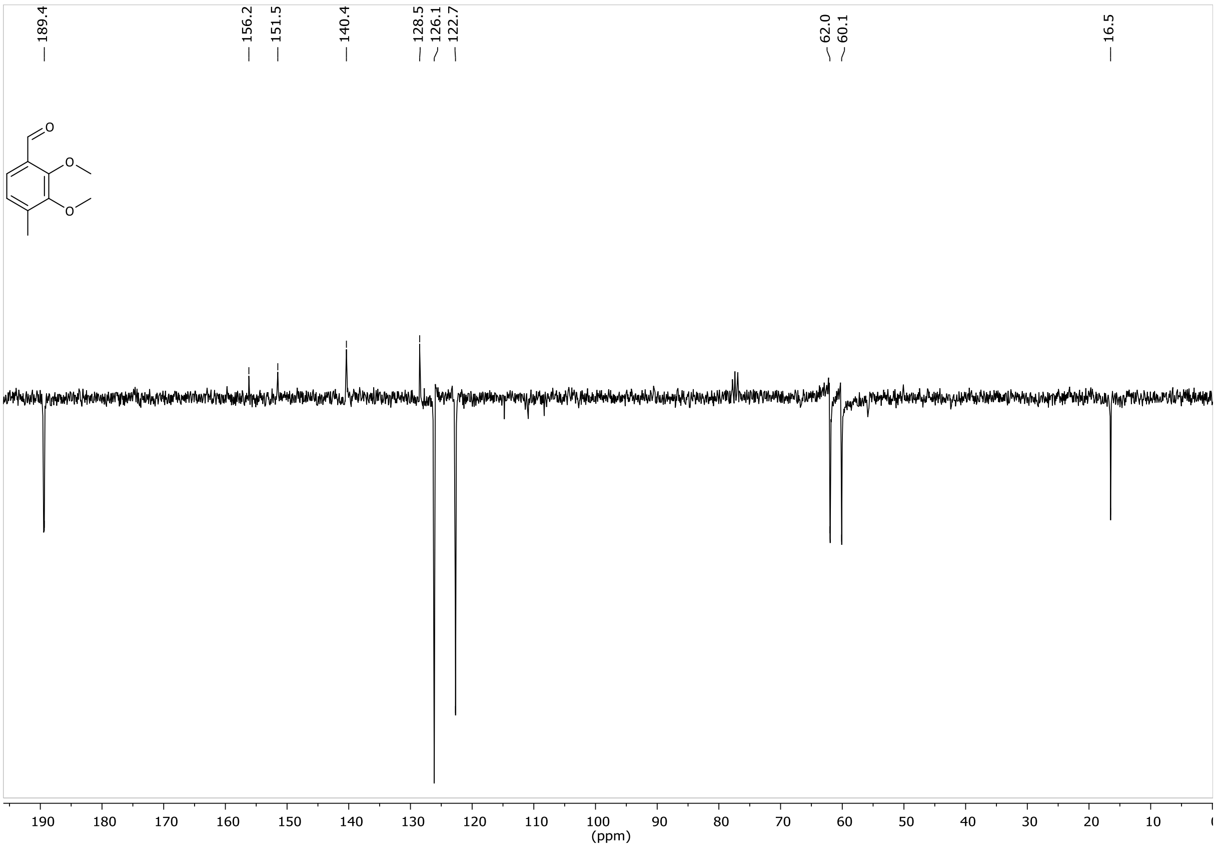

$^1H$ and $^{13}C$ NMR spectra of compound **9b**

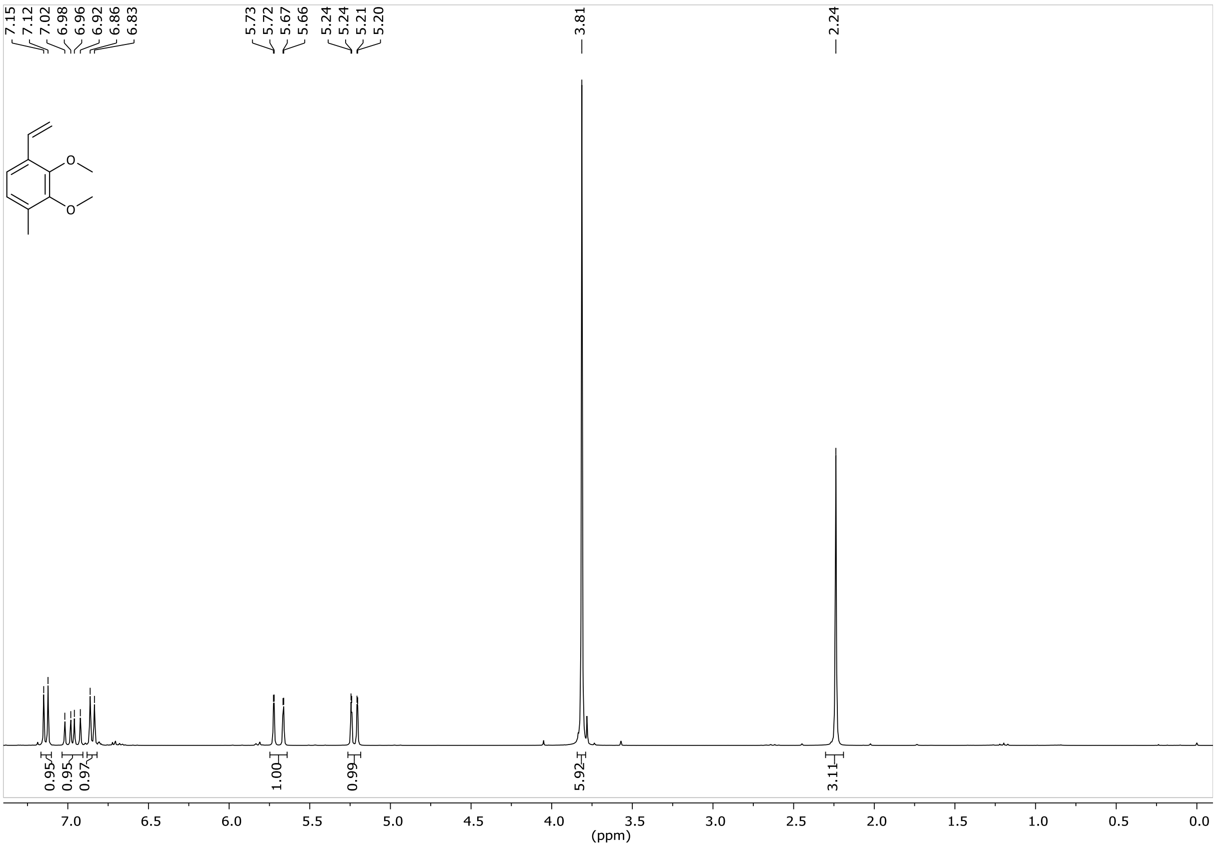


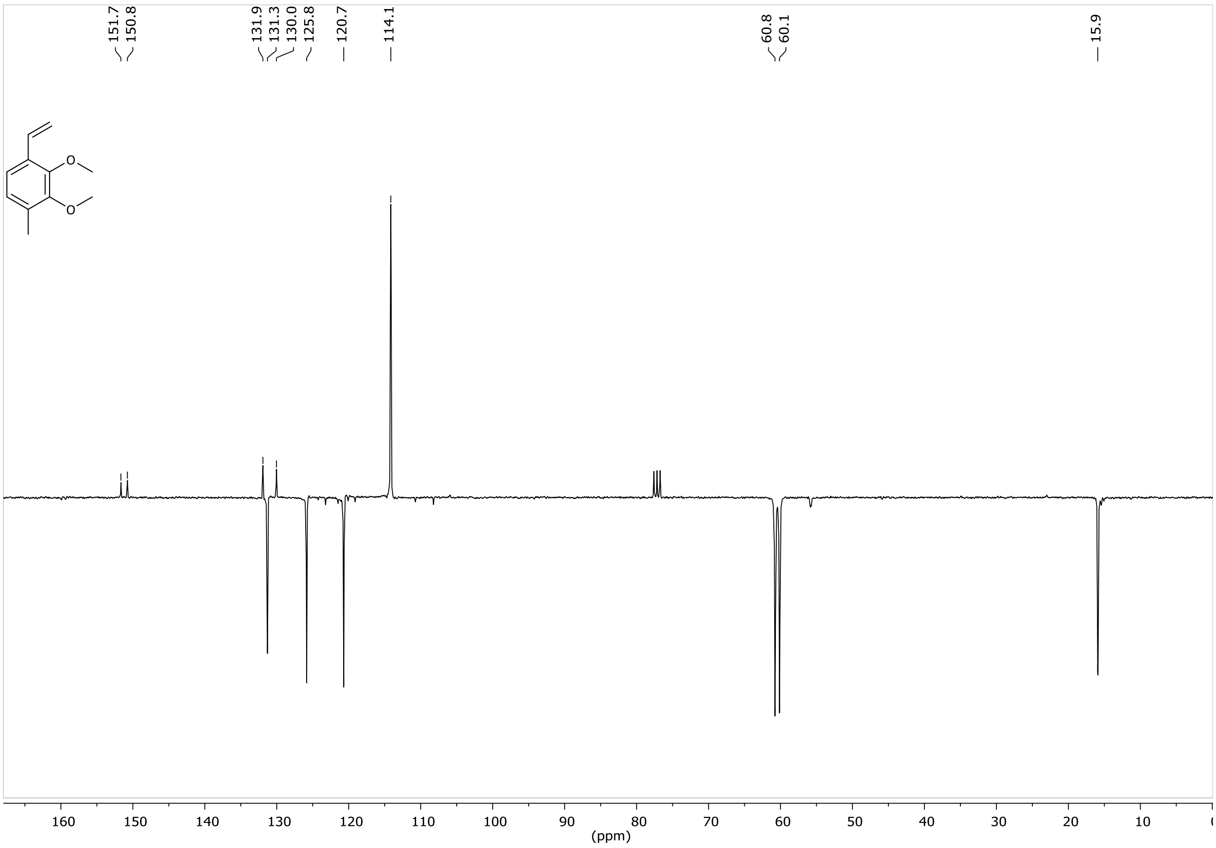

$^{1}$H and $^{13}$C NMR spectra of compound **8a**

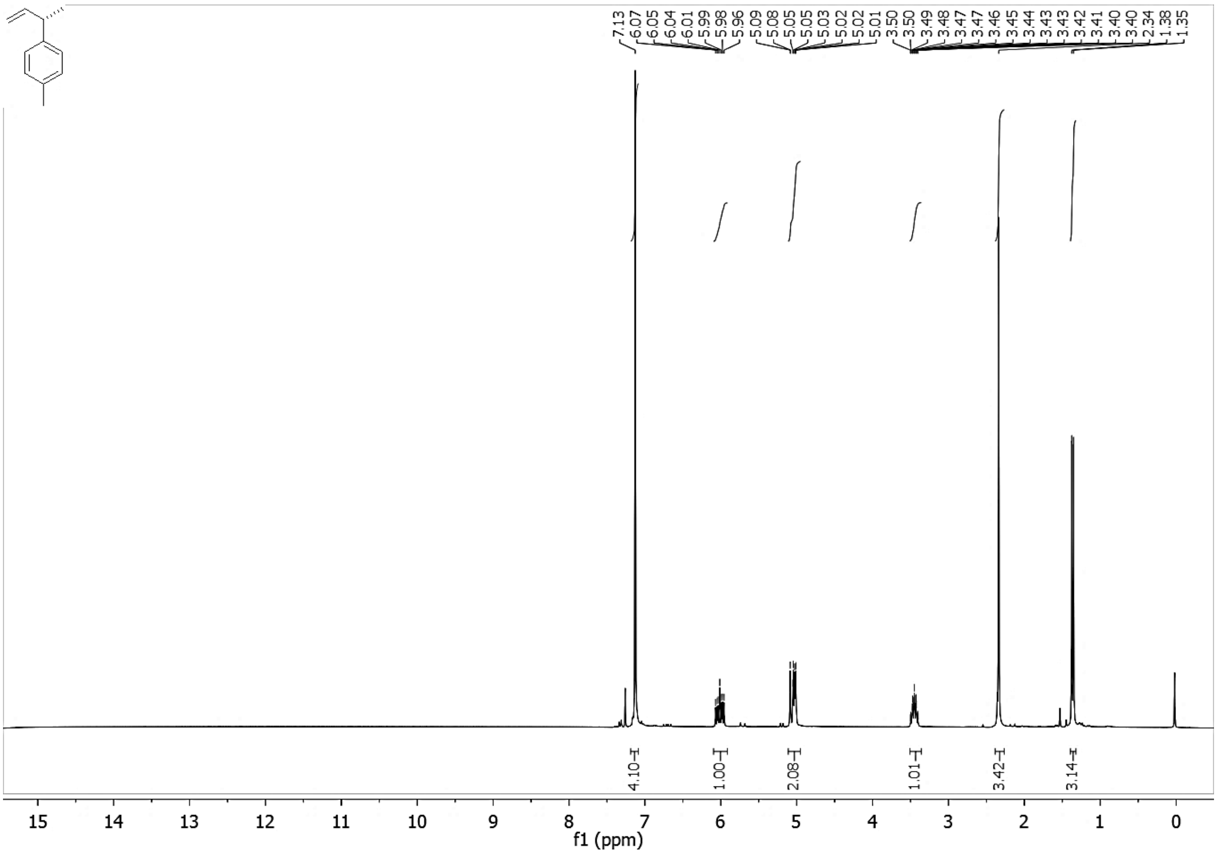


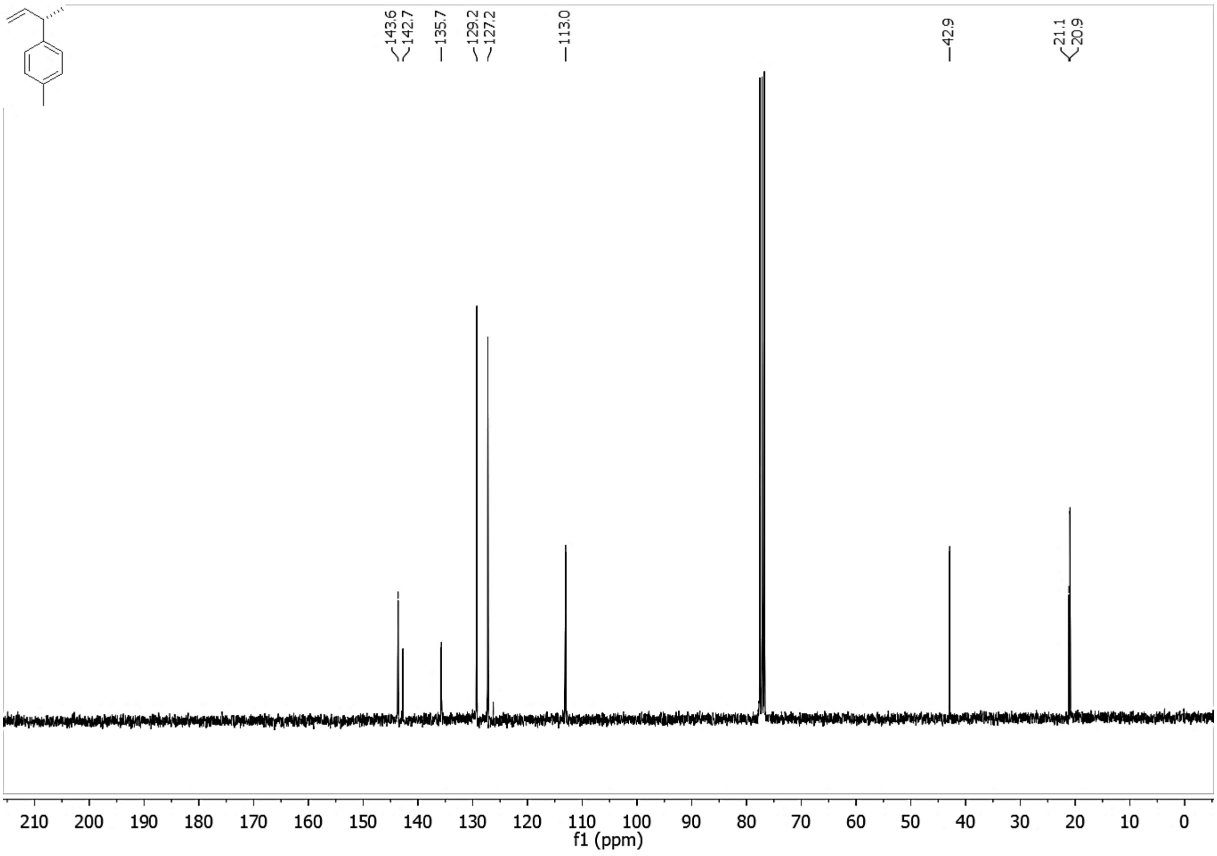

$^{1}H$ and $^{13}C$ NMR spectra of compound **8b**

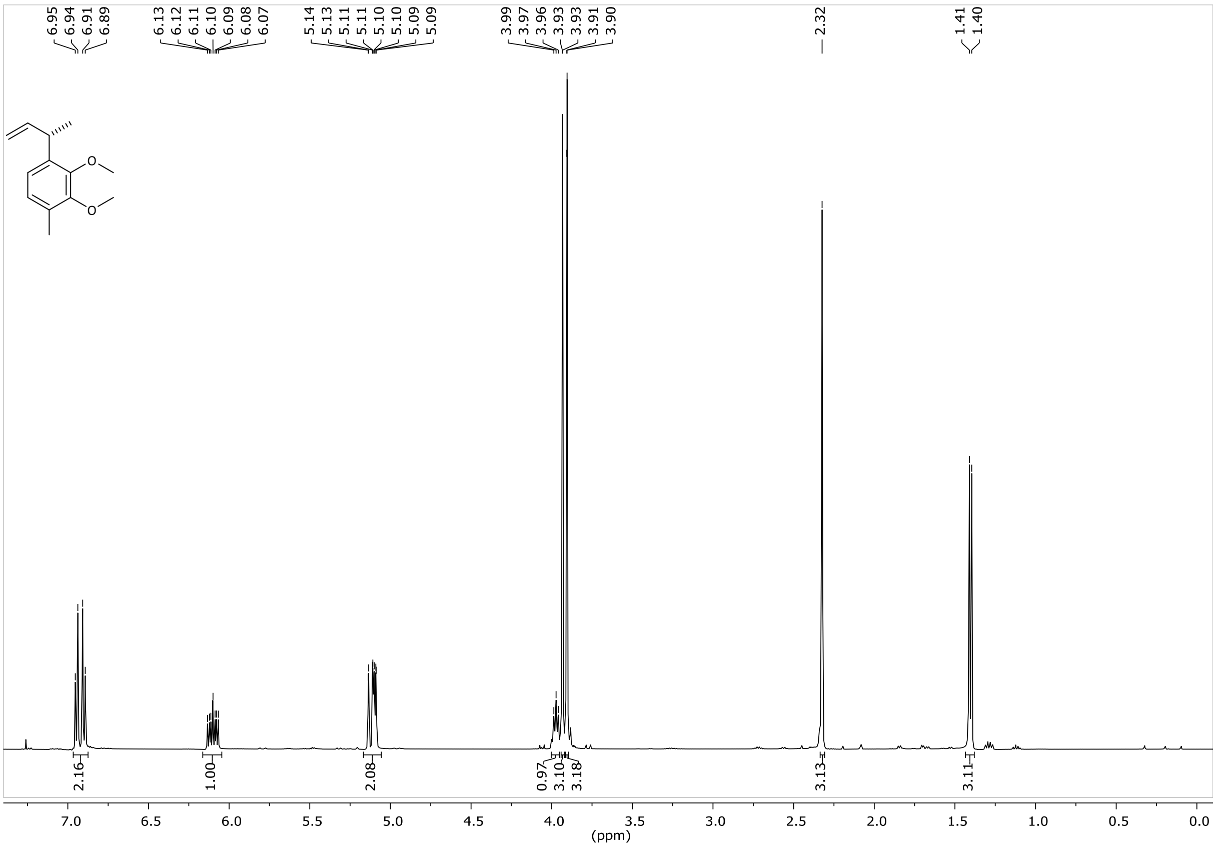


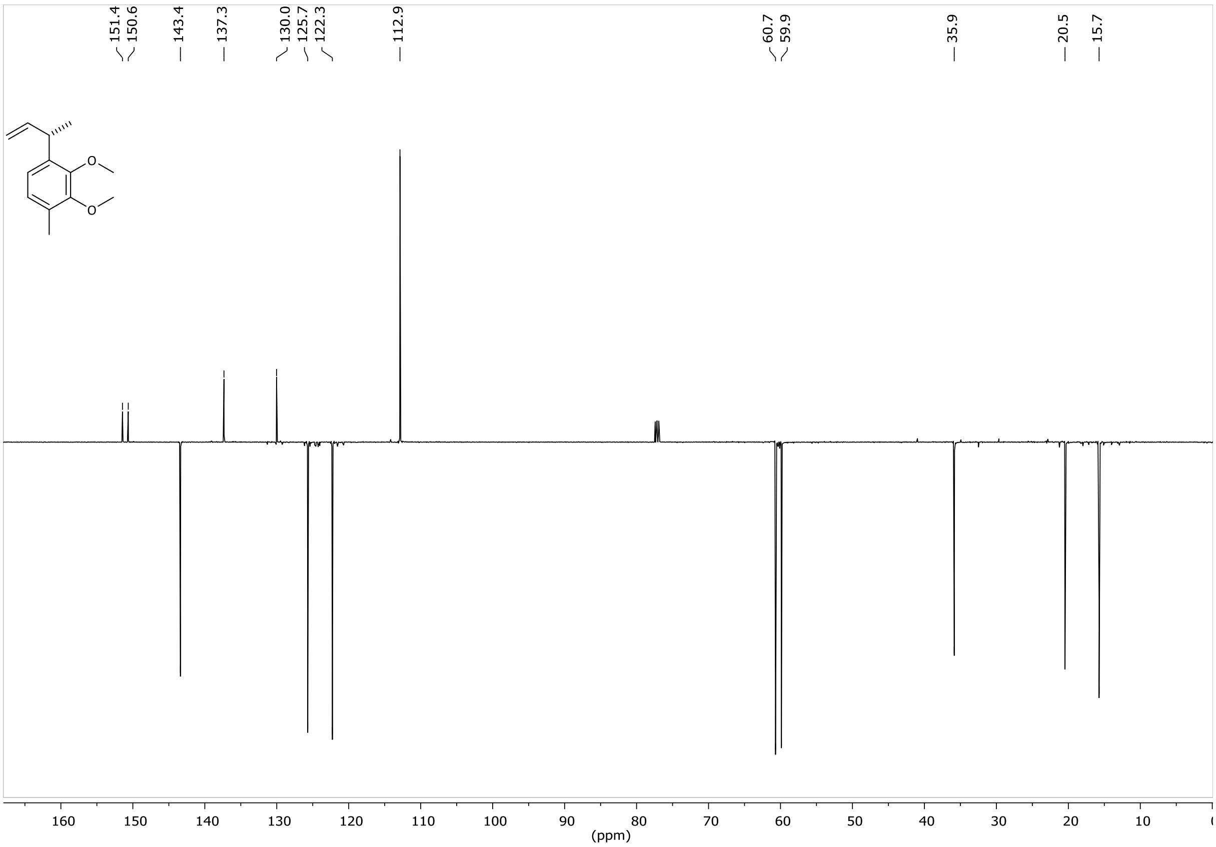

$^1H$ and $^{13}C$ NMR spectra of compound **12**

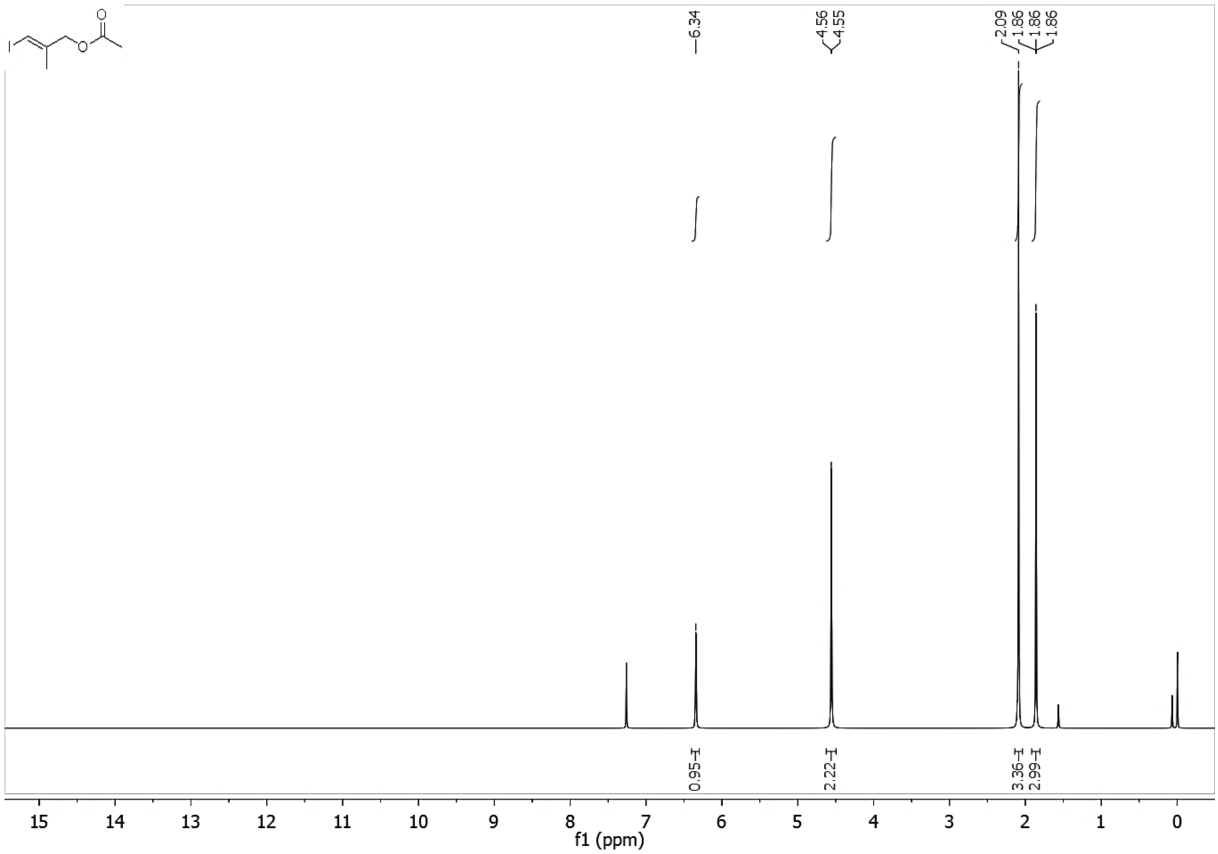


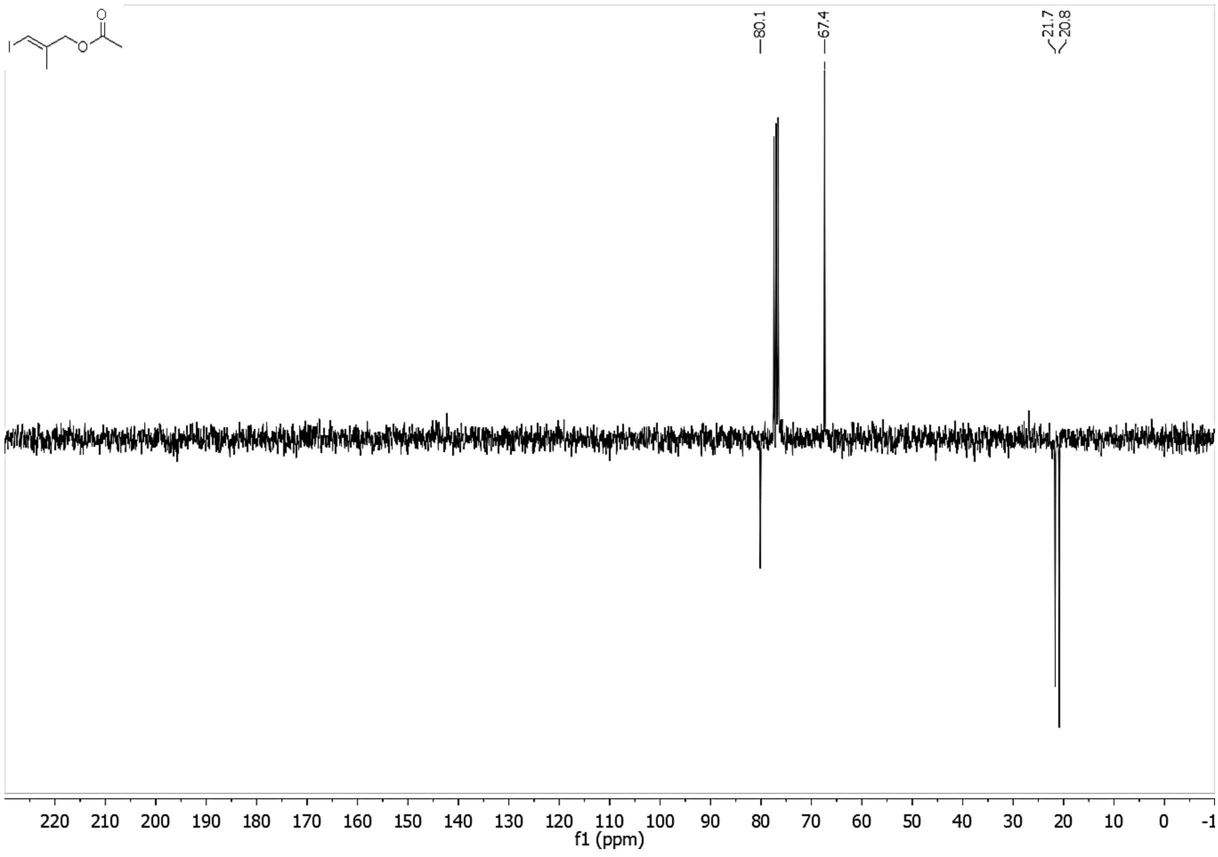

$^{1}$H and $^{13}$C NMR spectra of compound **7a**

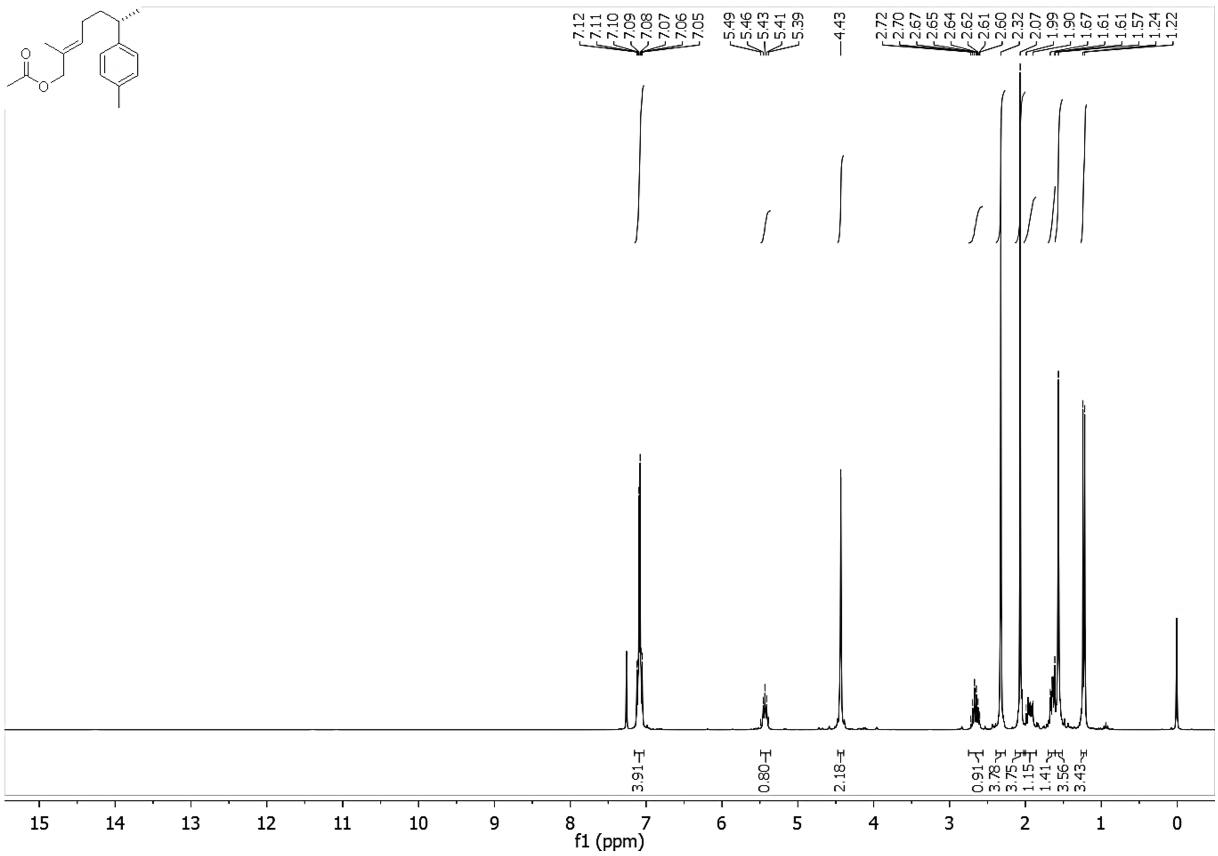


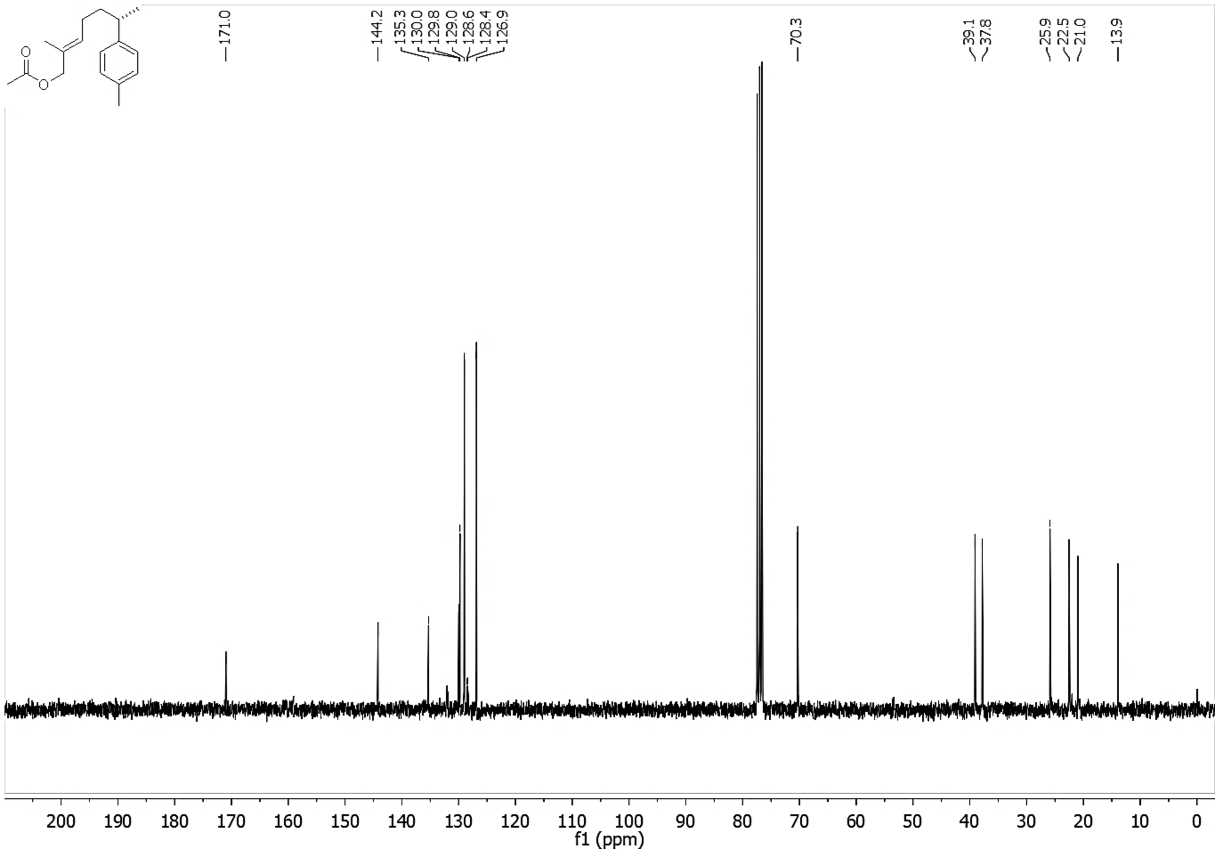

$^{1}$H and $^{13}$C NMR spectra of compound **7b**

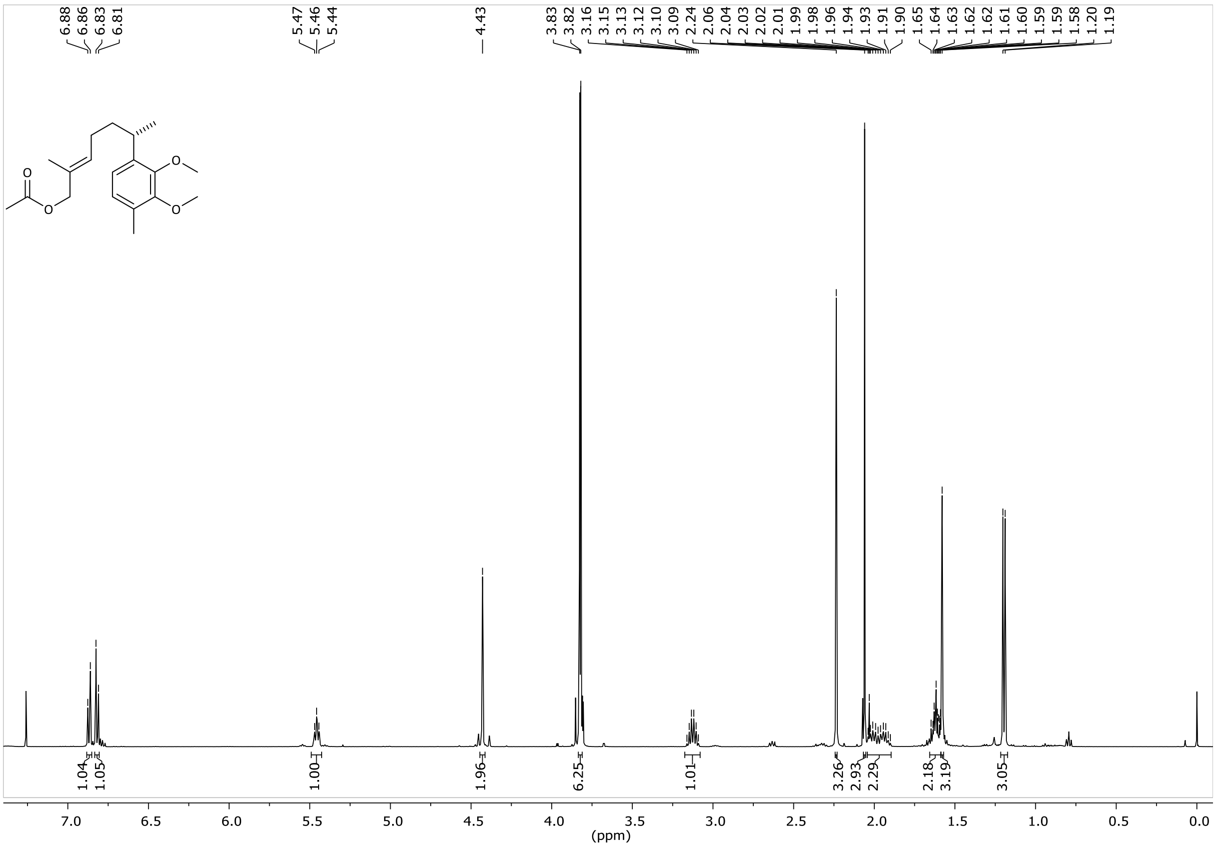


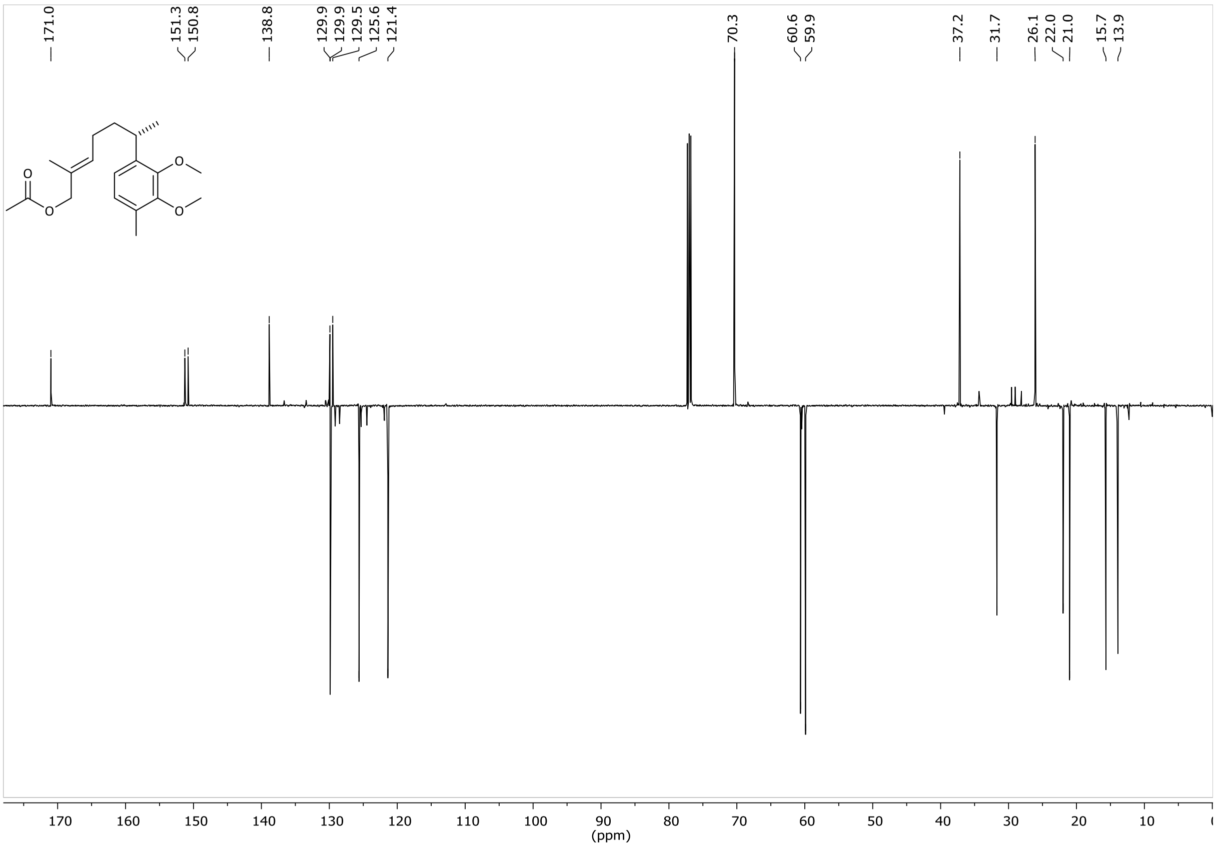

$^{1}$H and $^{13}$C NMR spectra of compound **6a** (as a diastereomeric mixture of *trans*-**6a**/*cis*-**6a** 90:10)

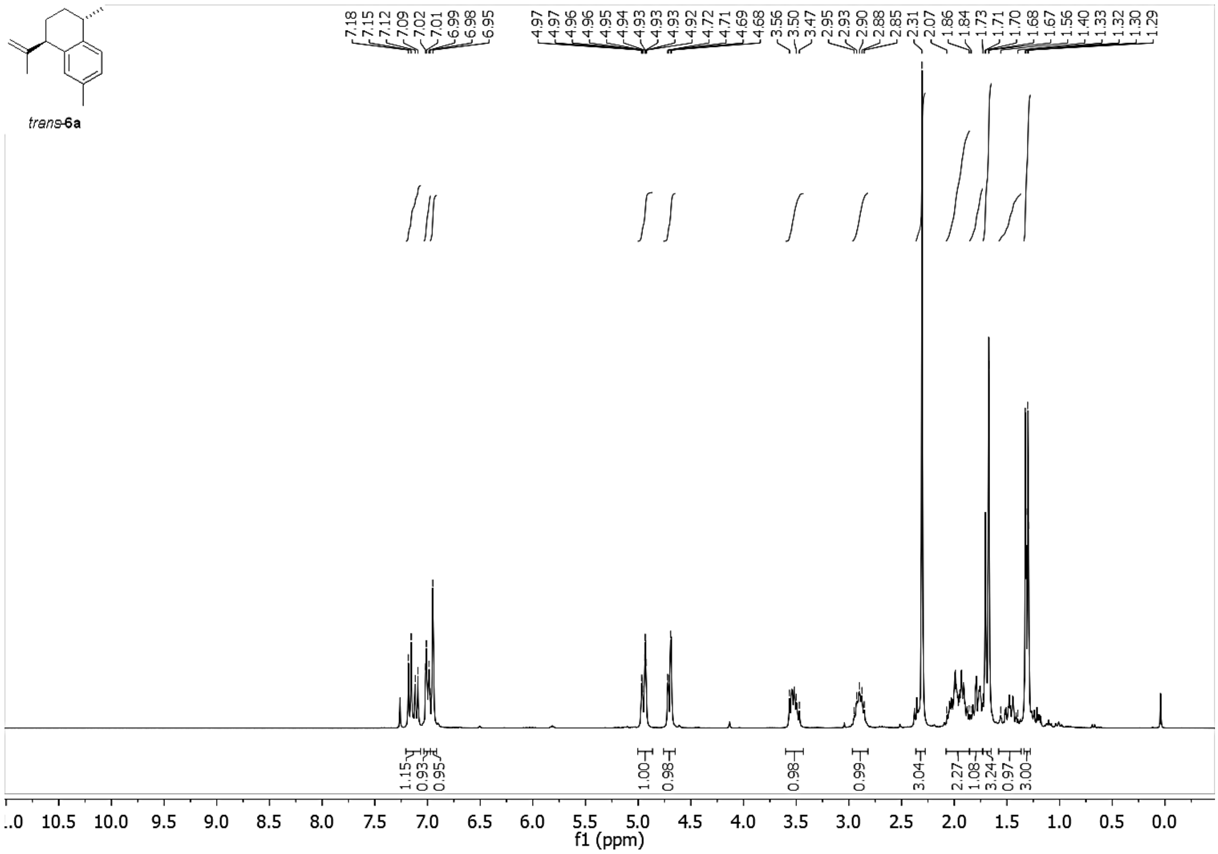


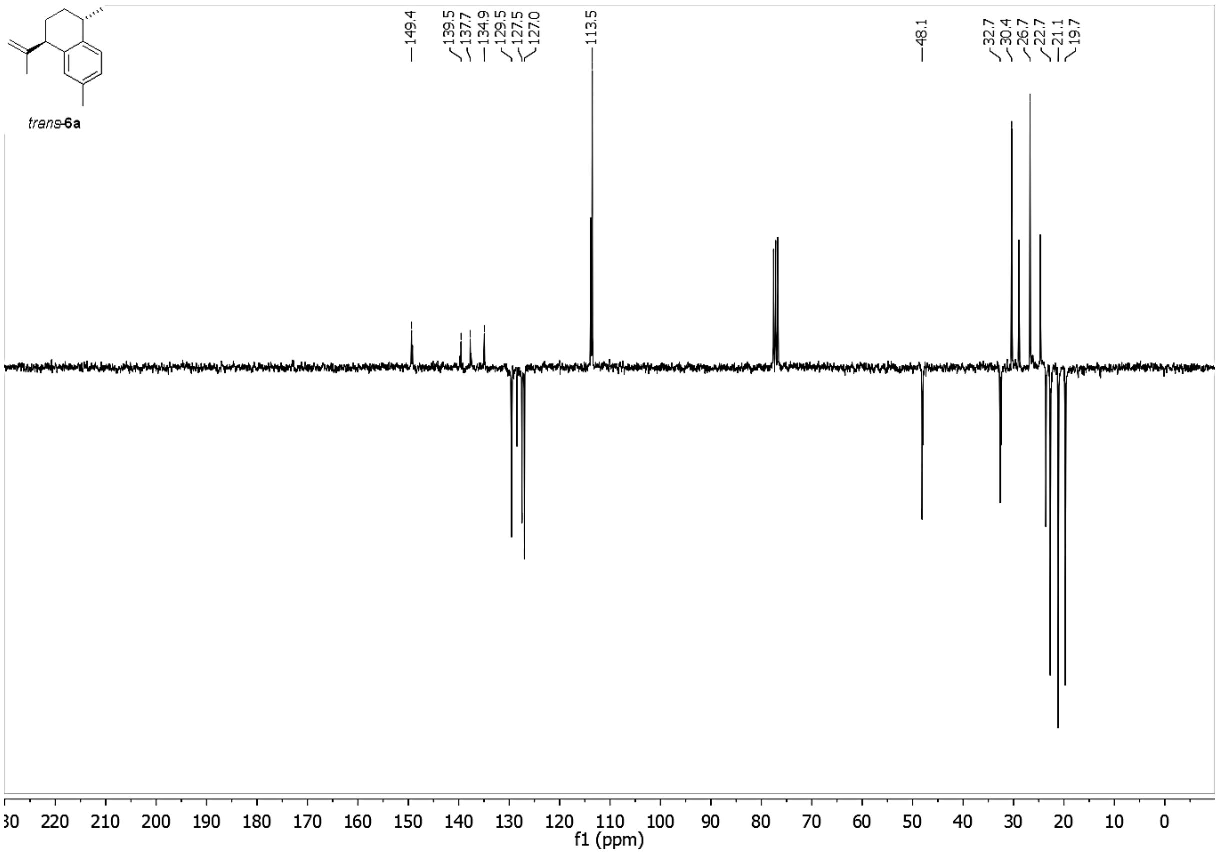

$^{1}$H and $^{13}$C NMR spectra of compound **6b** (as a diastereomeric mixture of *trans*-**6b**/*cis*-**6b** 90:10)

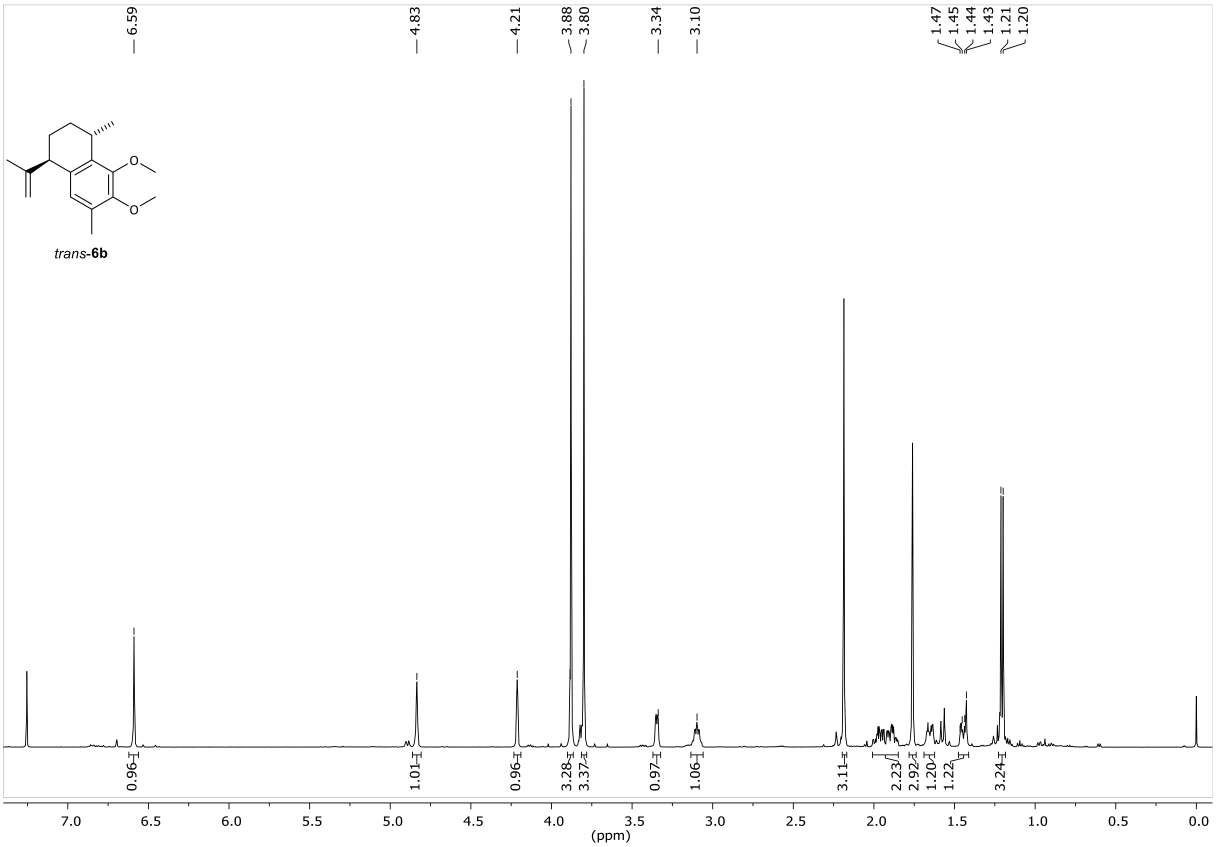


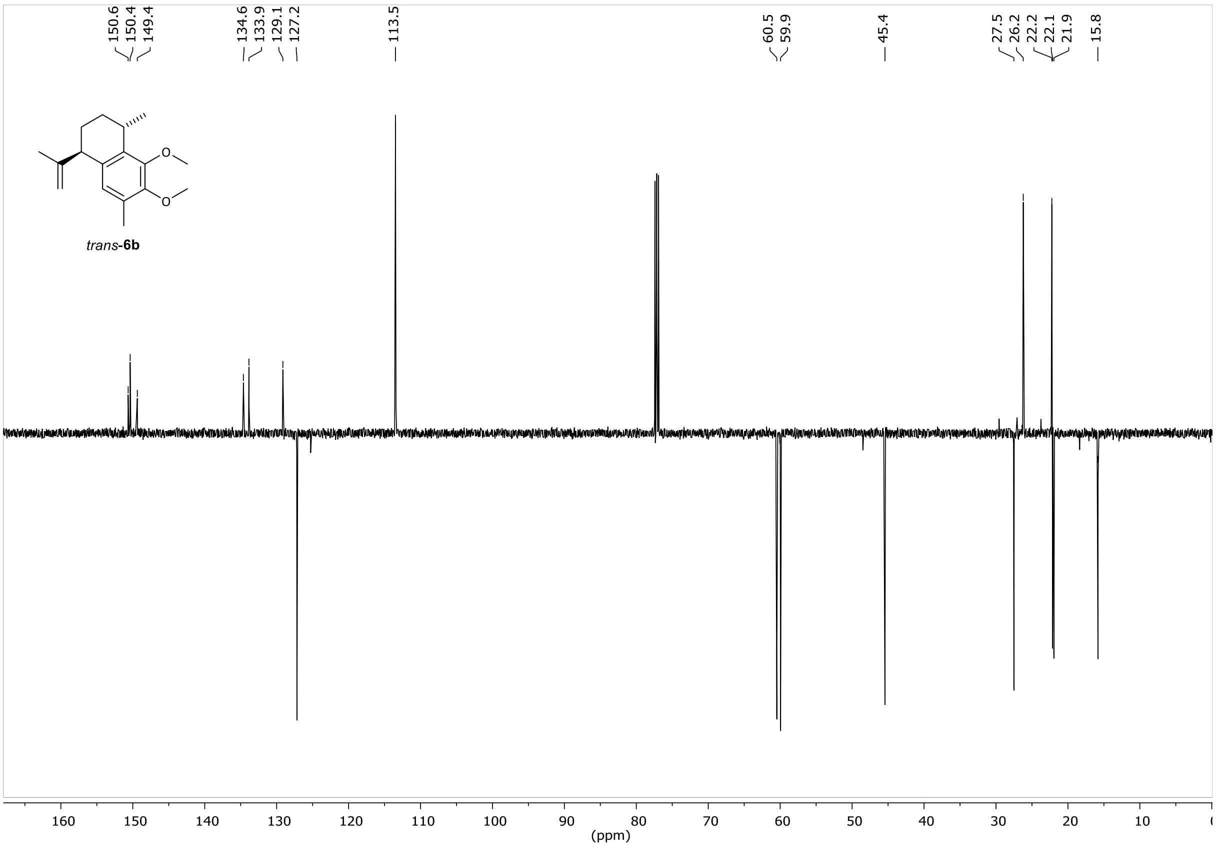

<sup></sup>

$^{1}$H and $^{13}$C NMR spectra of compound **13a**

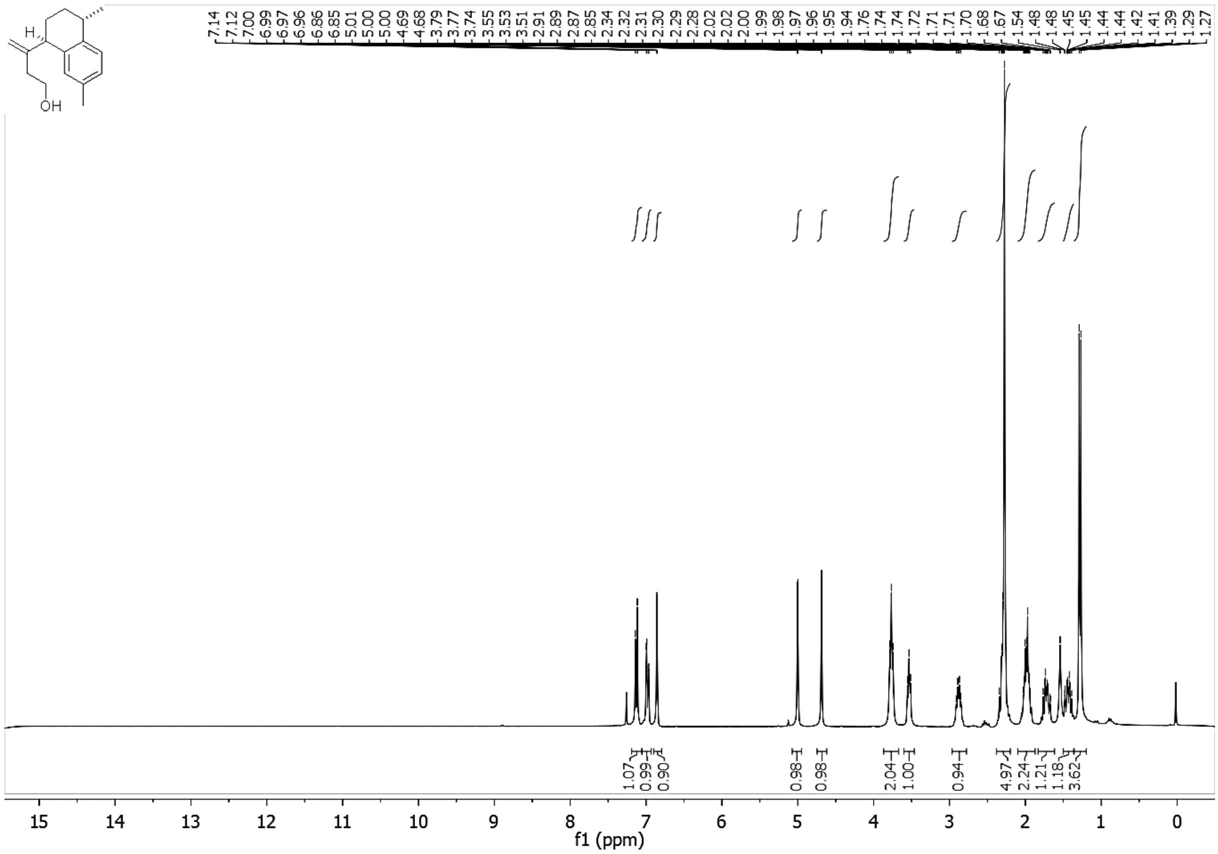


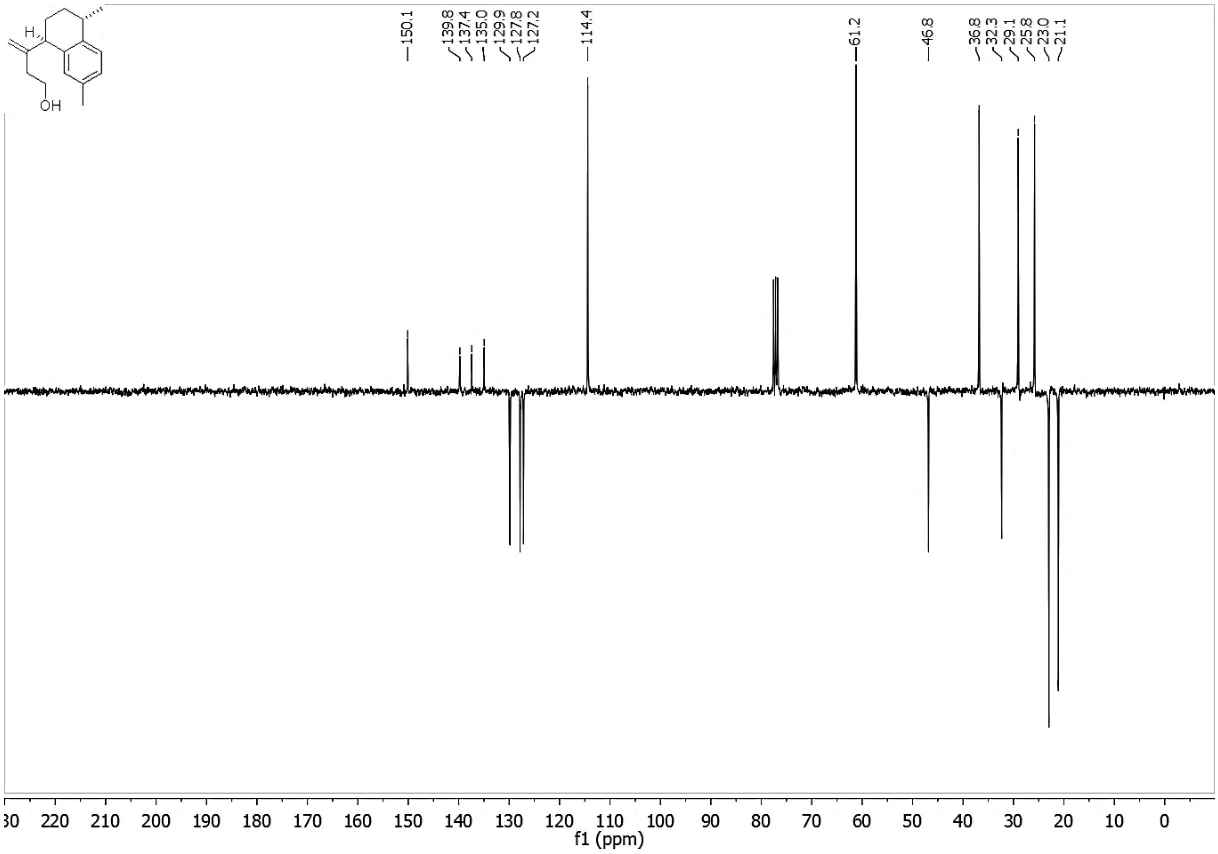

$^{1}$H and $^{13}$C NMR spectra of compound **13b**

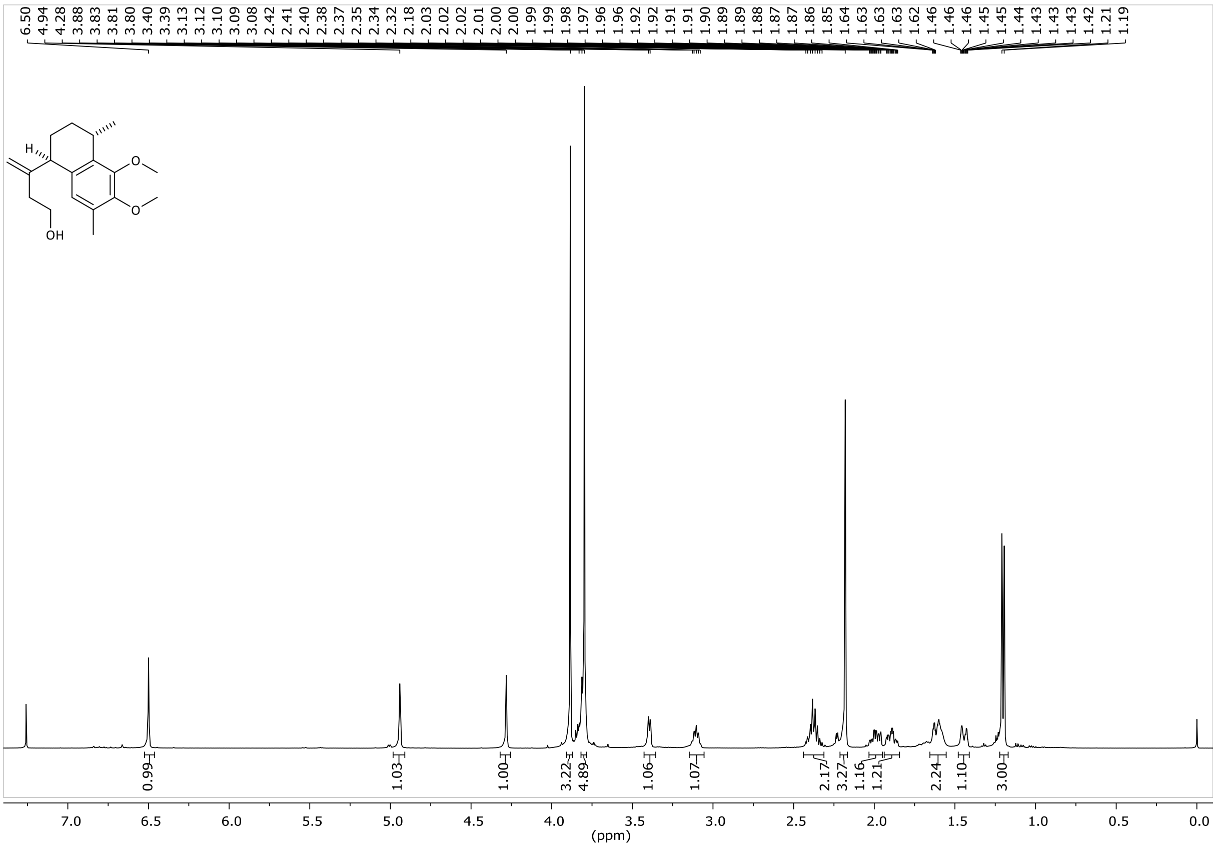


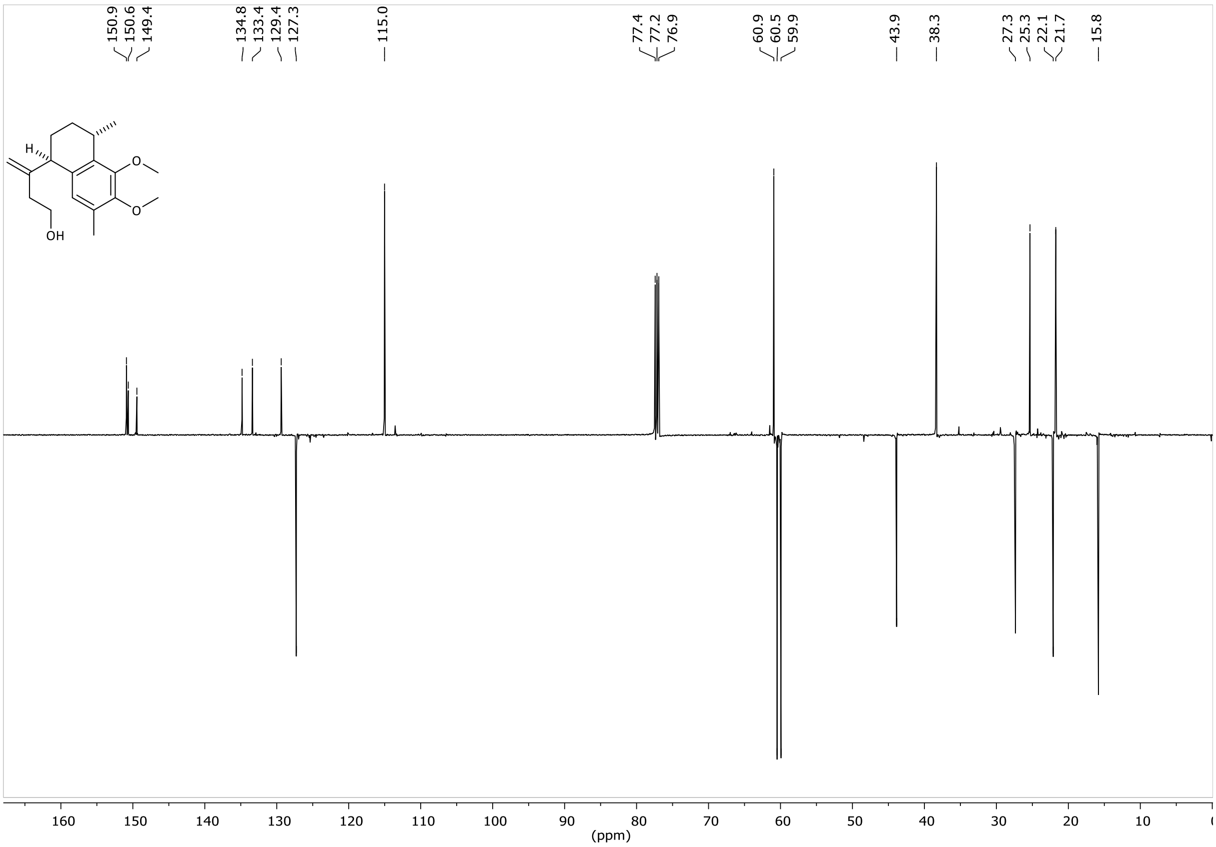

<sup></sup>

$^{1}$H and $^{13}$C NMR spectra of compound **15a**

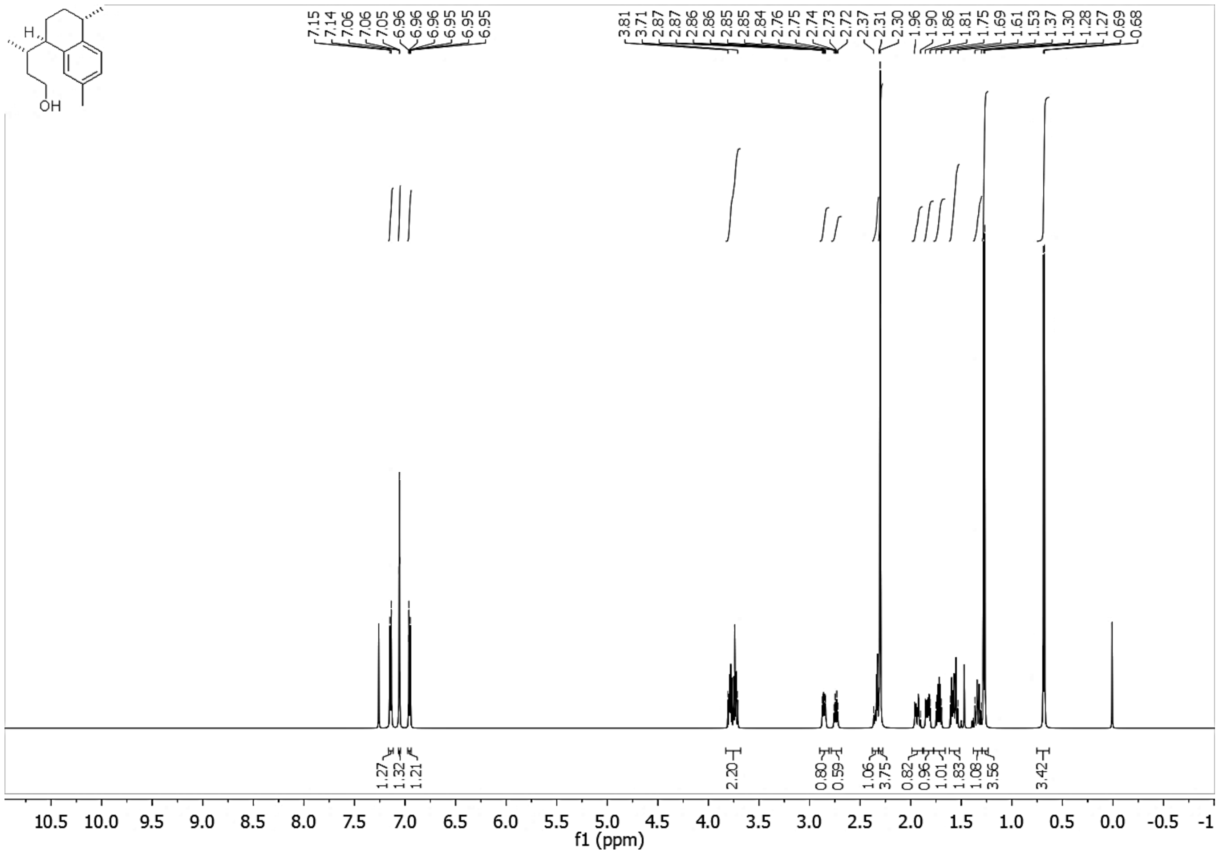


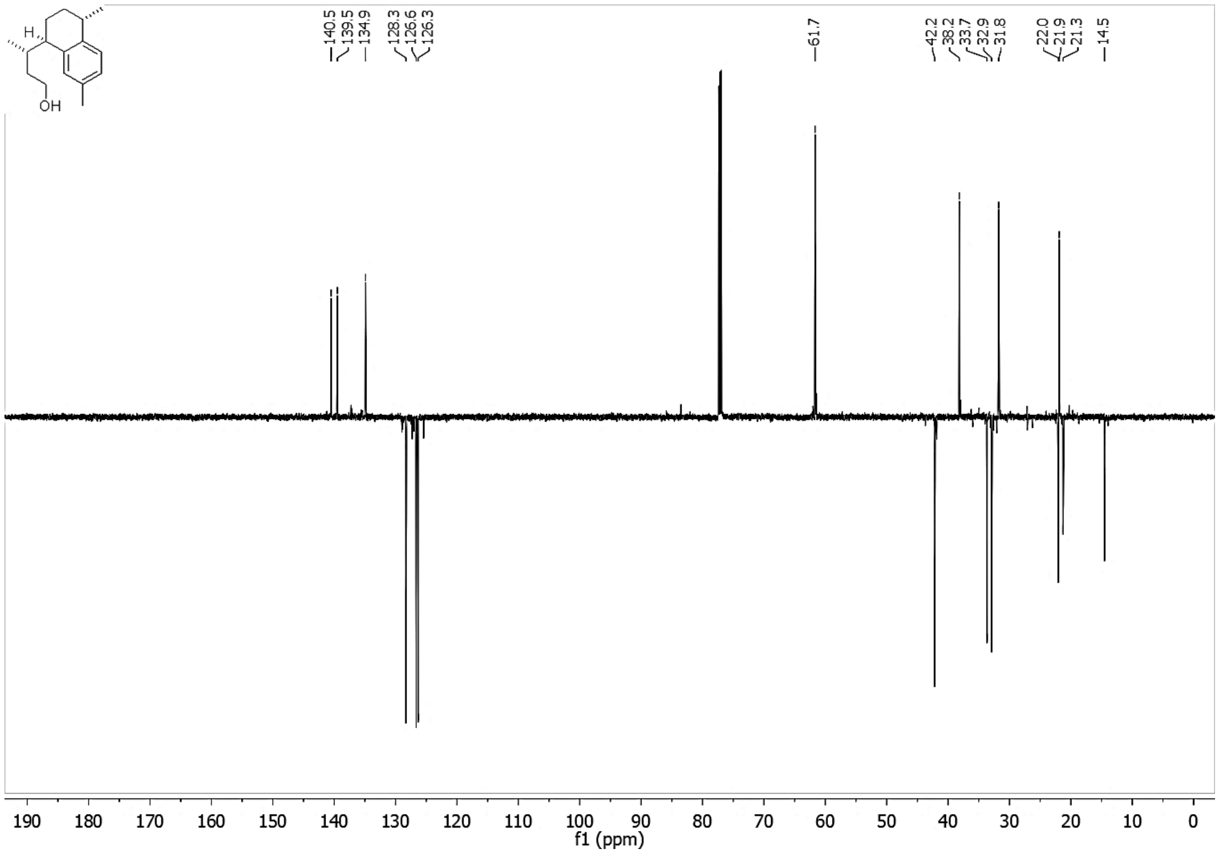

<sup></sup>

$^1$H and $^{13}$C NMR spectra of compound **15b**

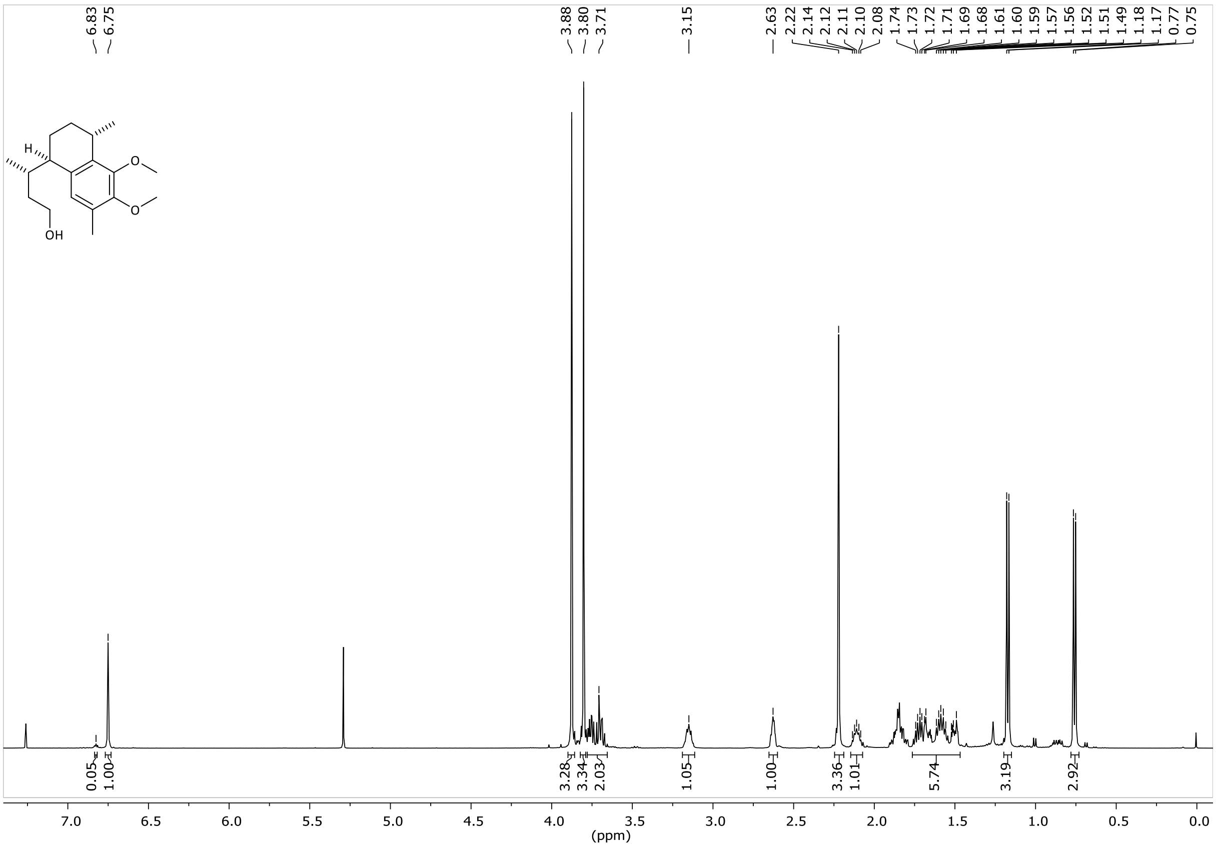


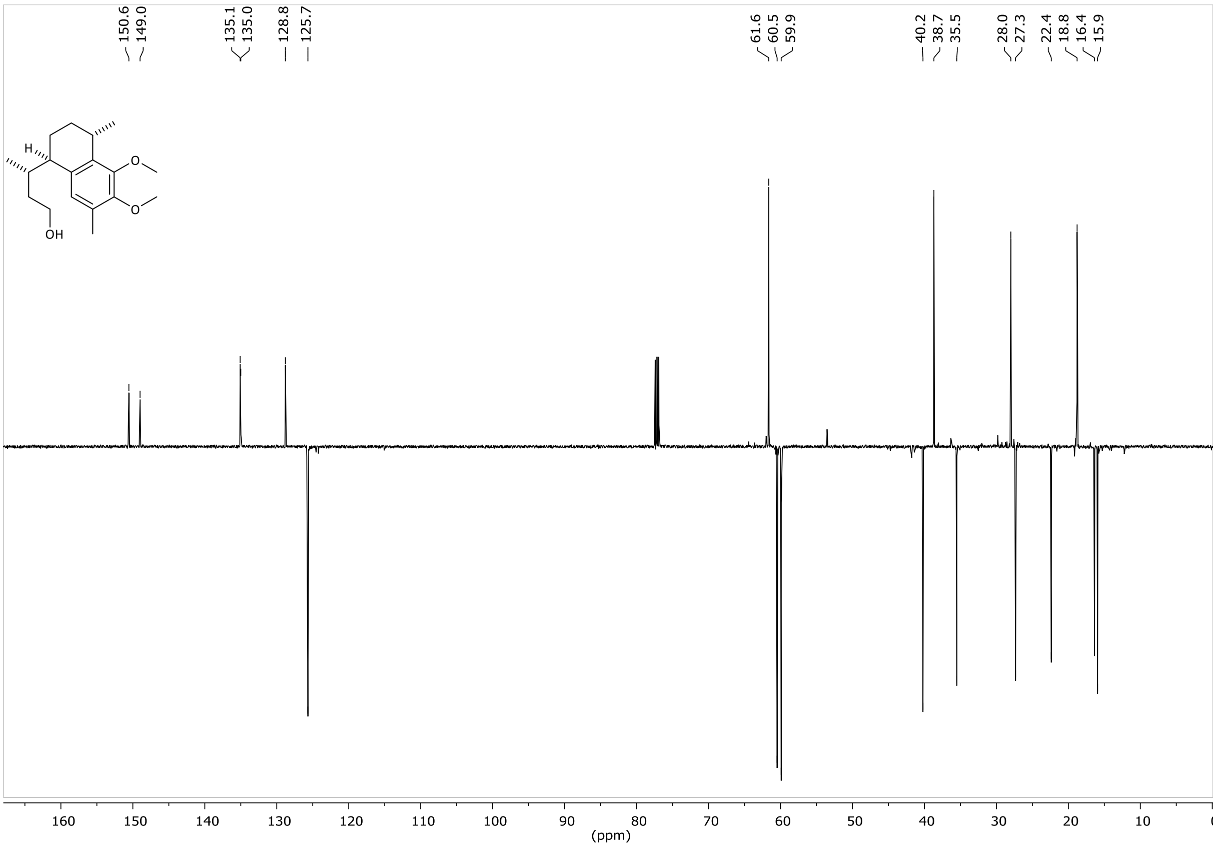

$^1H$ and $^{13}C$ NMR spectra of compound **16**

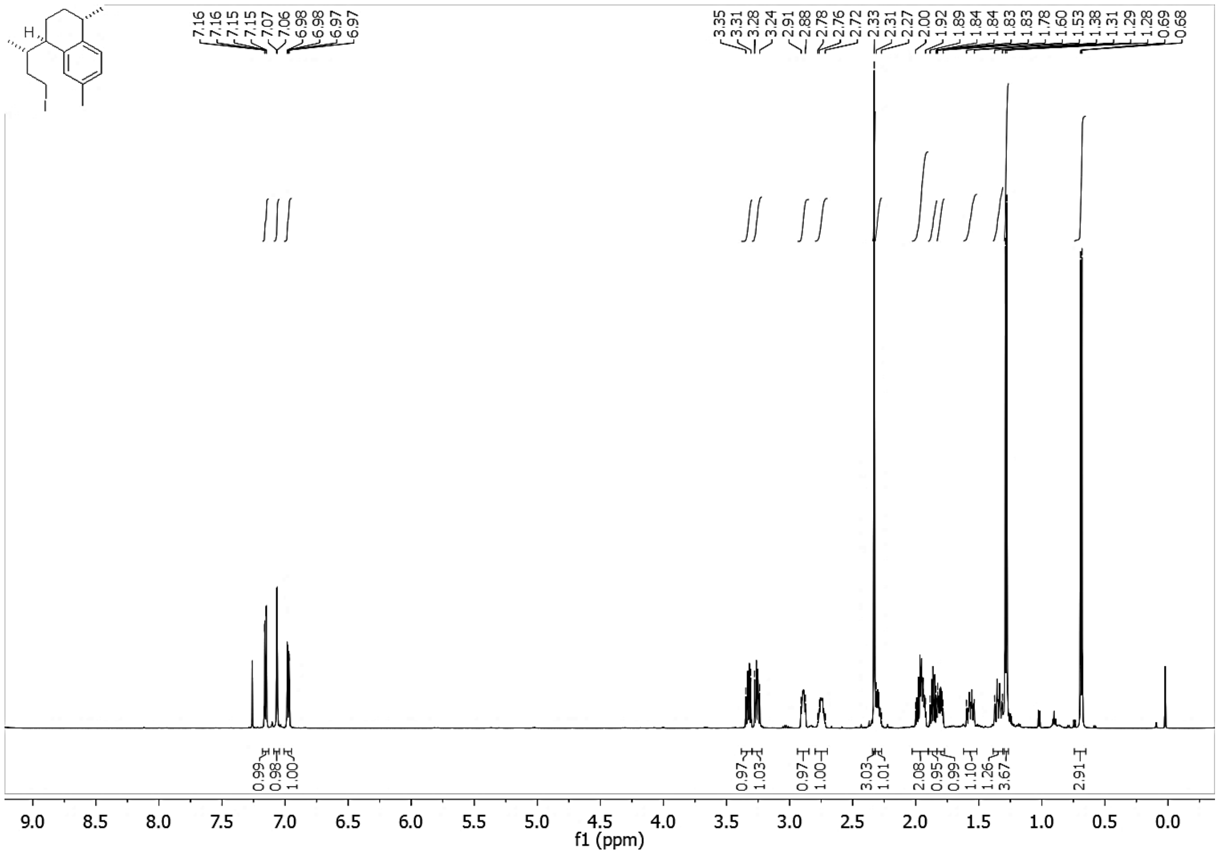


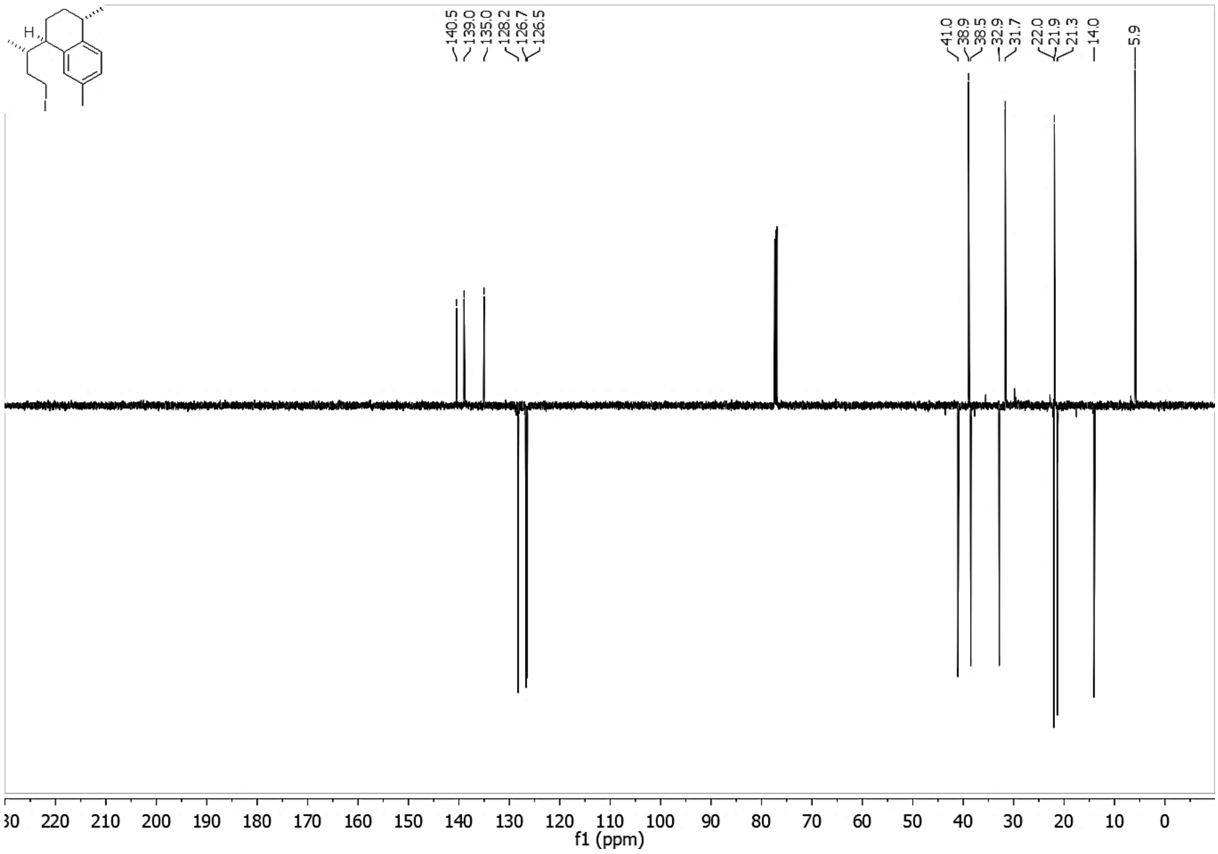

$^{1}H$ and $^{13}C$ NMR spectra of compound **1**

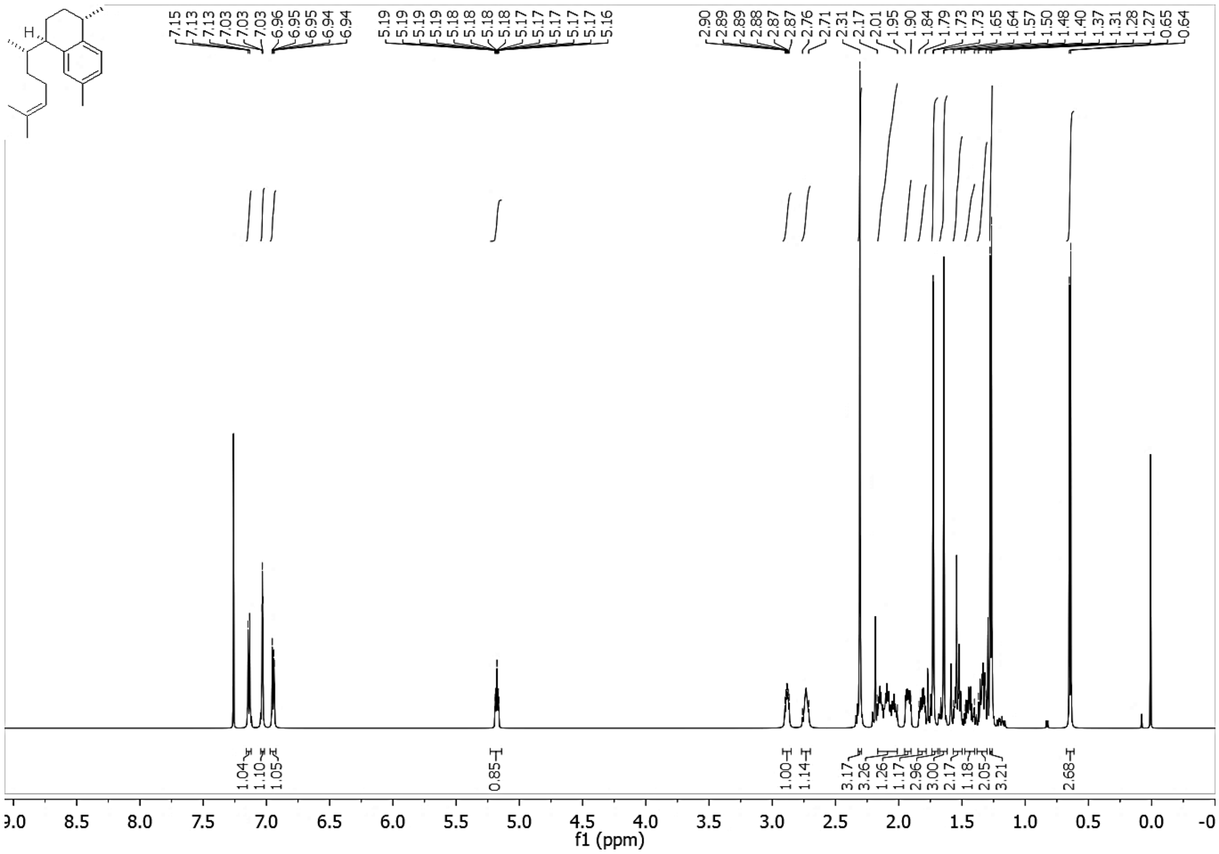

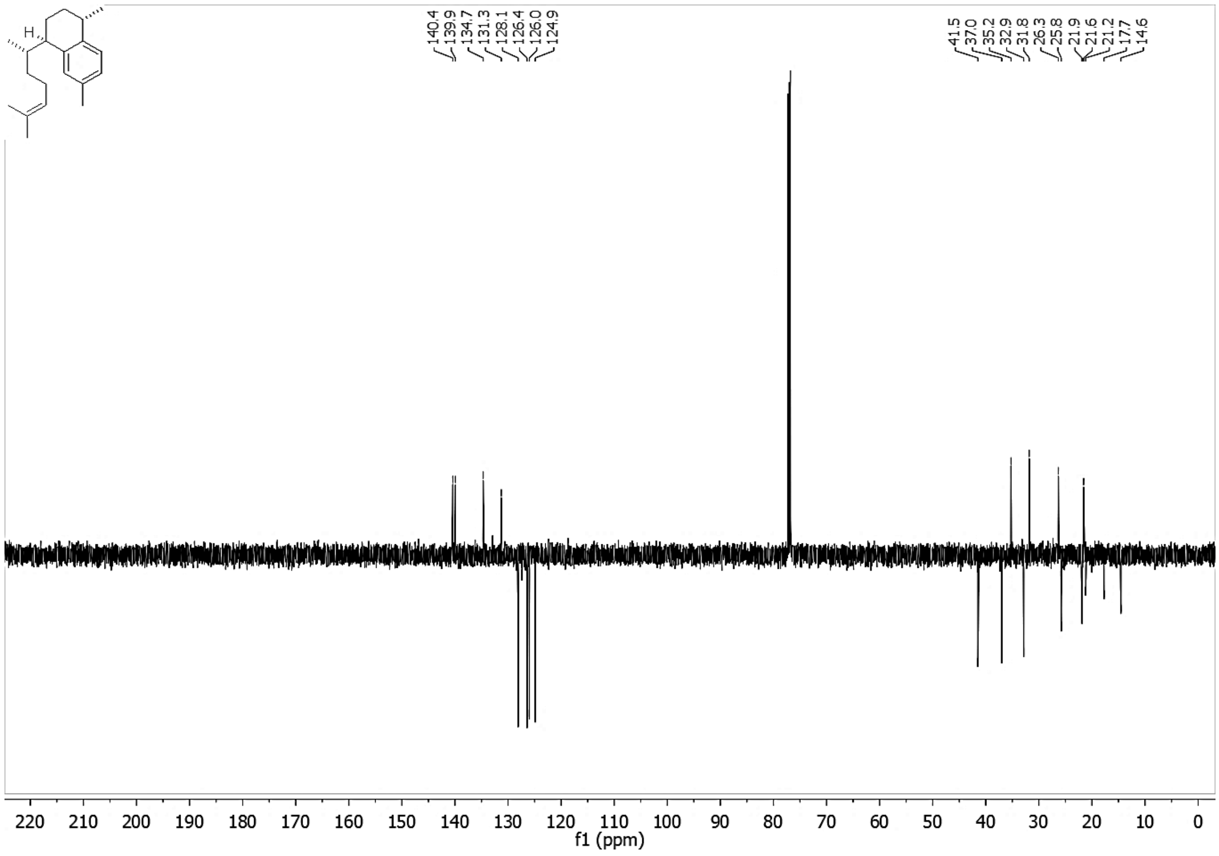

$^{1}$H and $^{13}$C NMR spectra of compound **17**

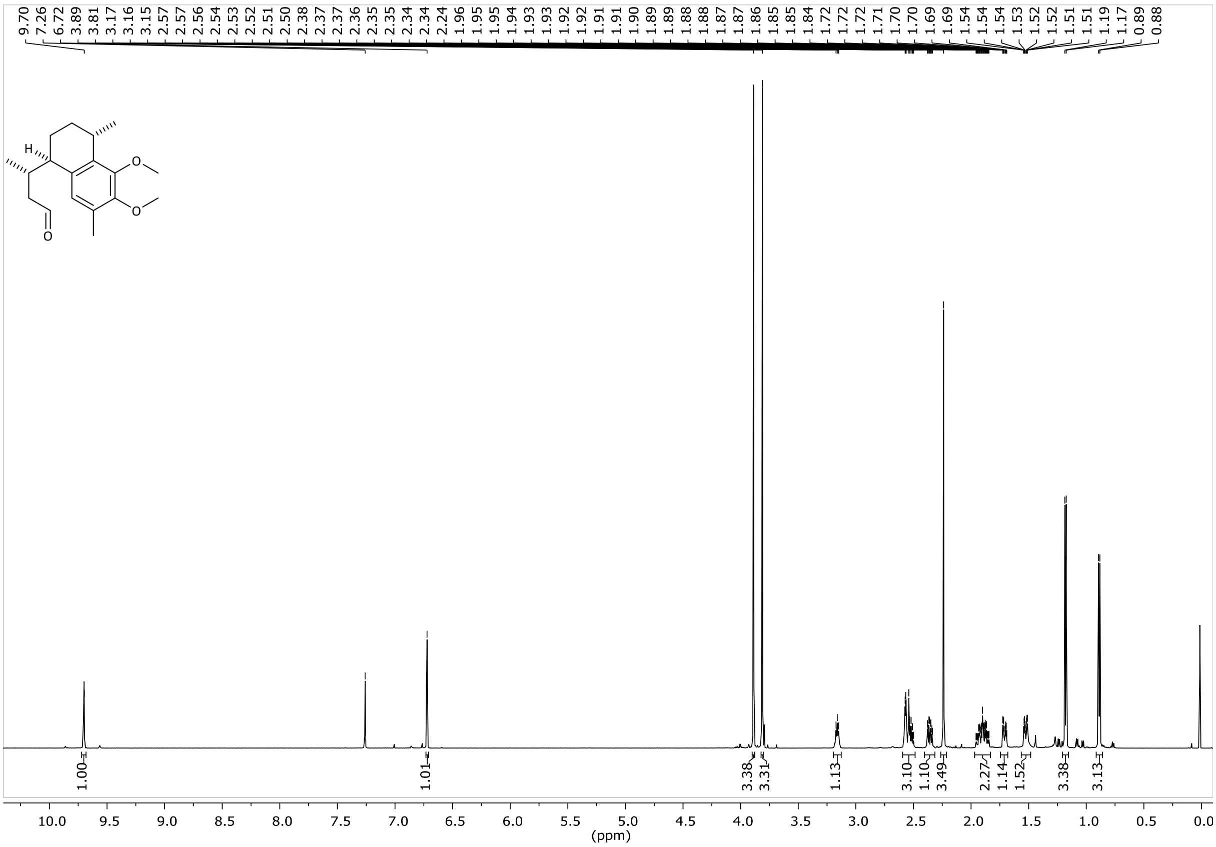


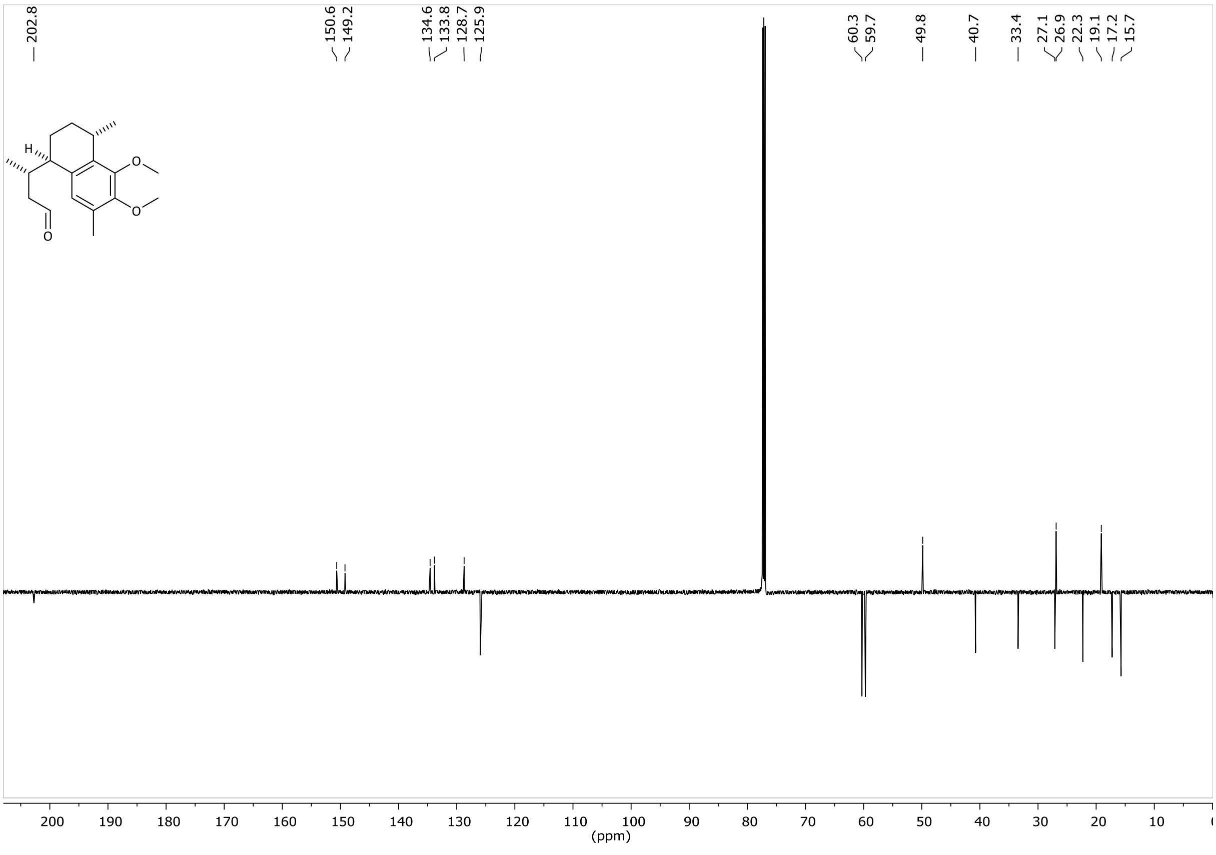

$^{1}H$ and $^{13}C$ NMR spectra of compound **18**

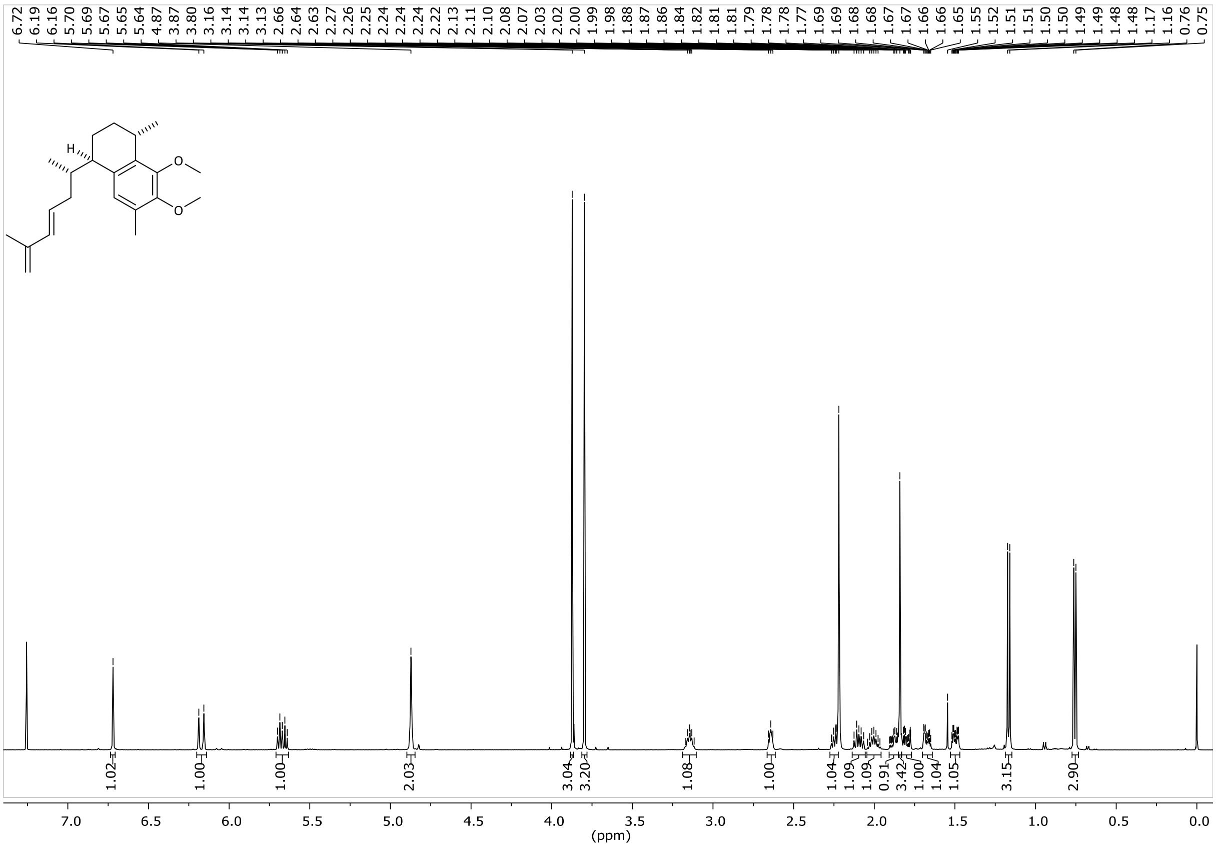


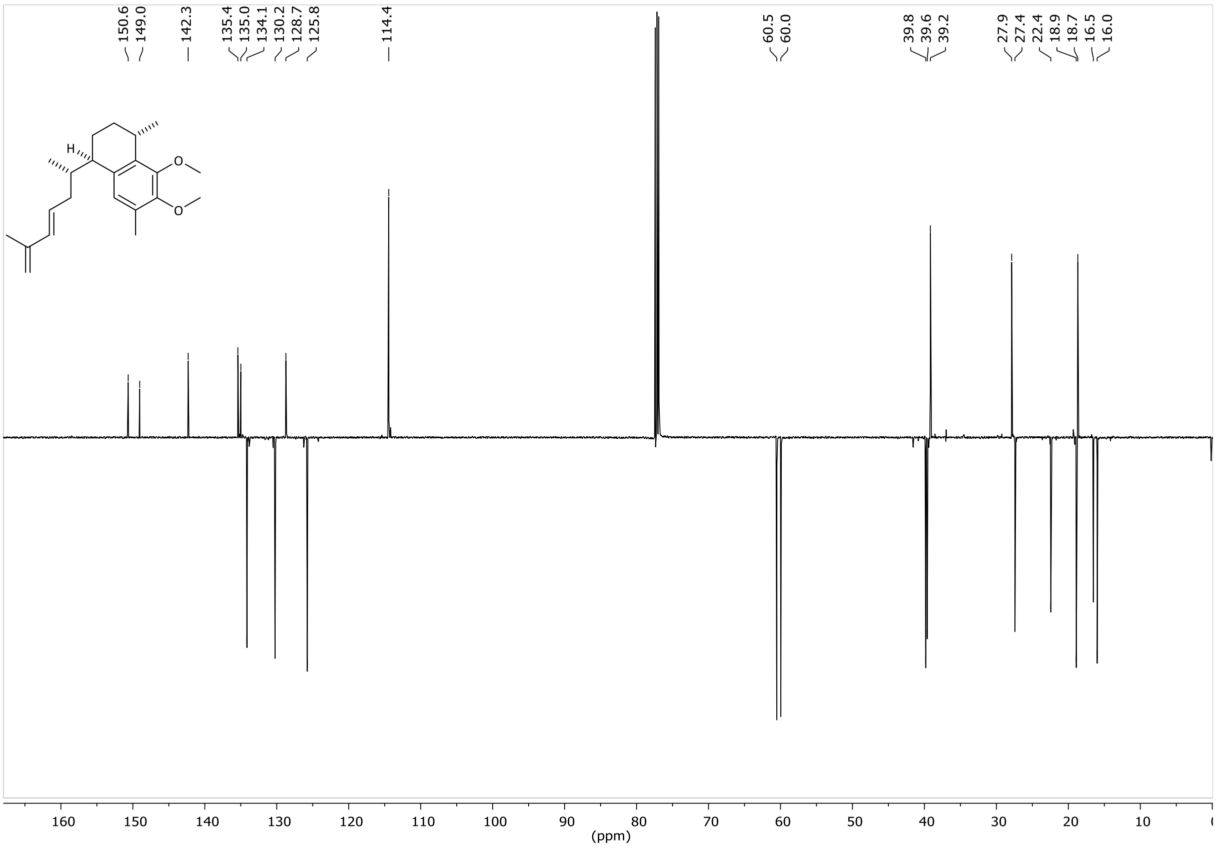

$^{1}H$ and $^{13}C$ NMR spectra of compound **19**

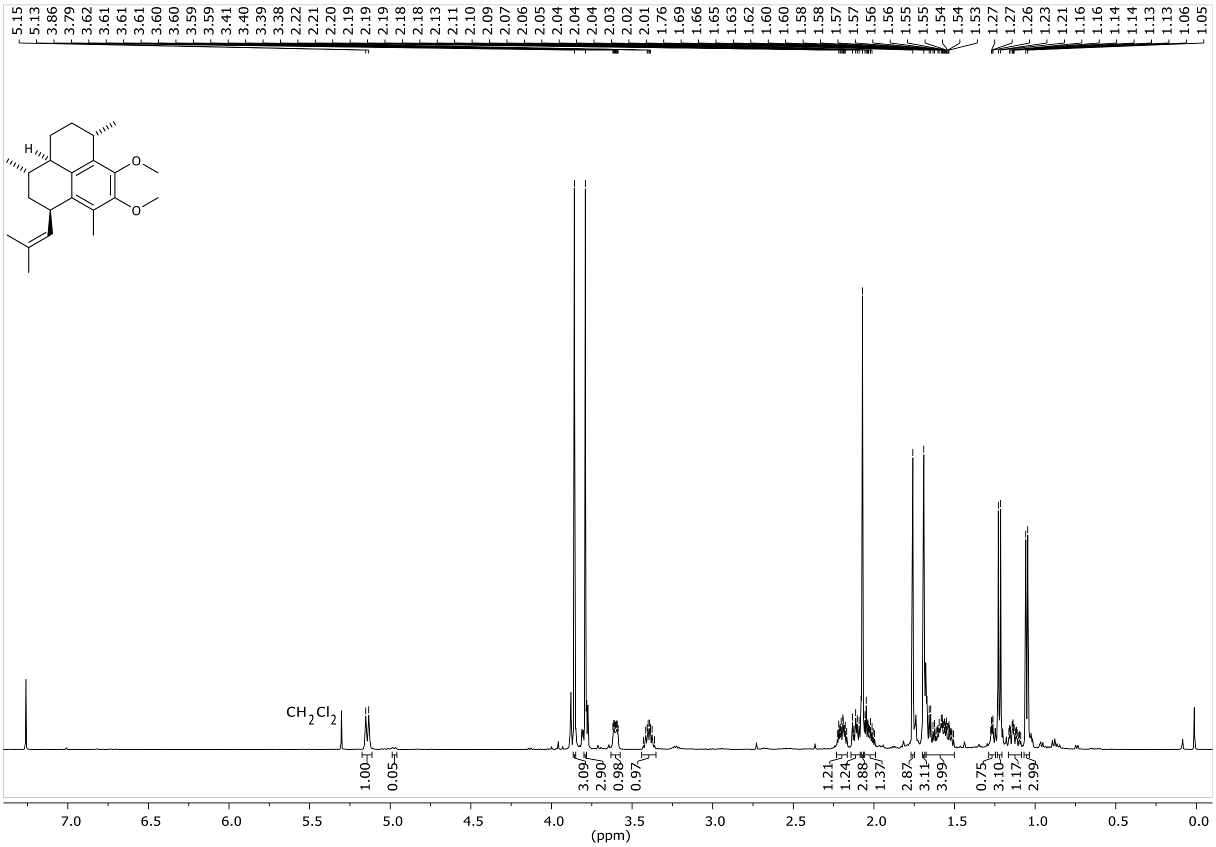


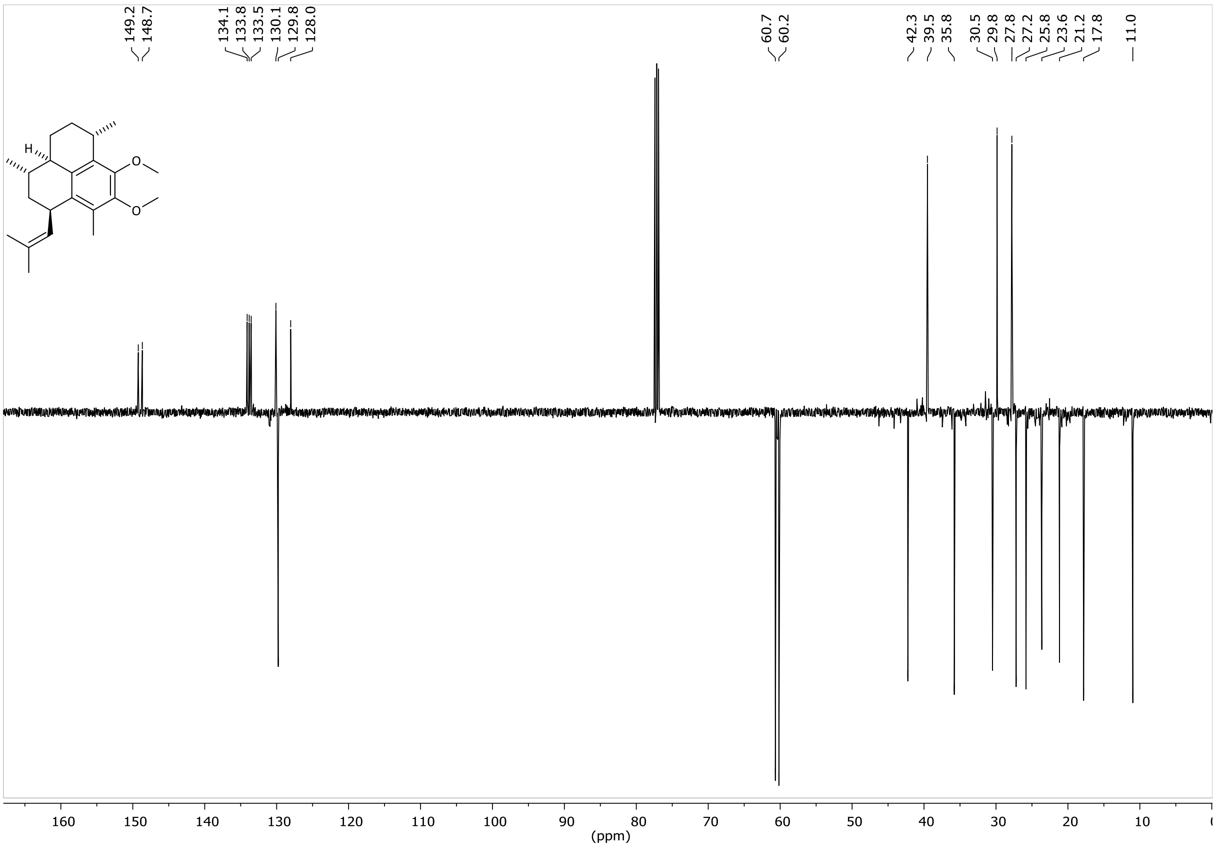

$^{1}$H and $^{13}$C NMR spectra of compound **20**

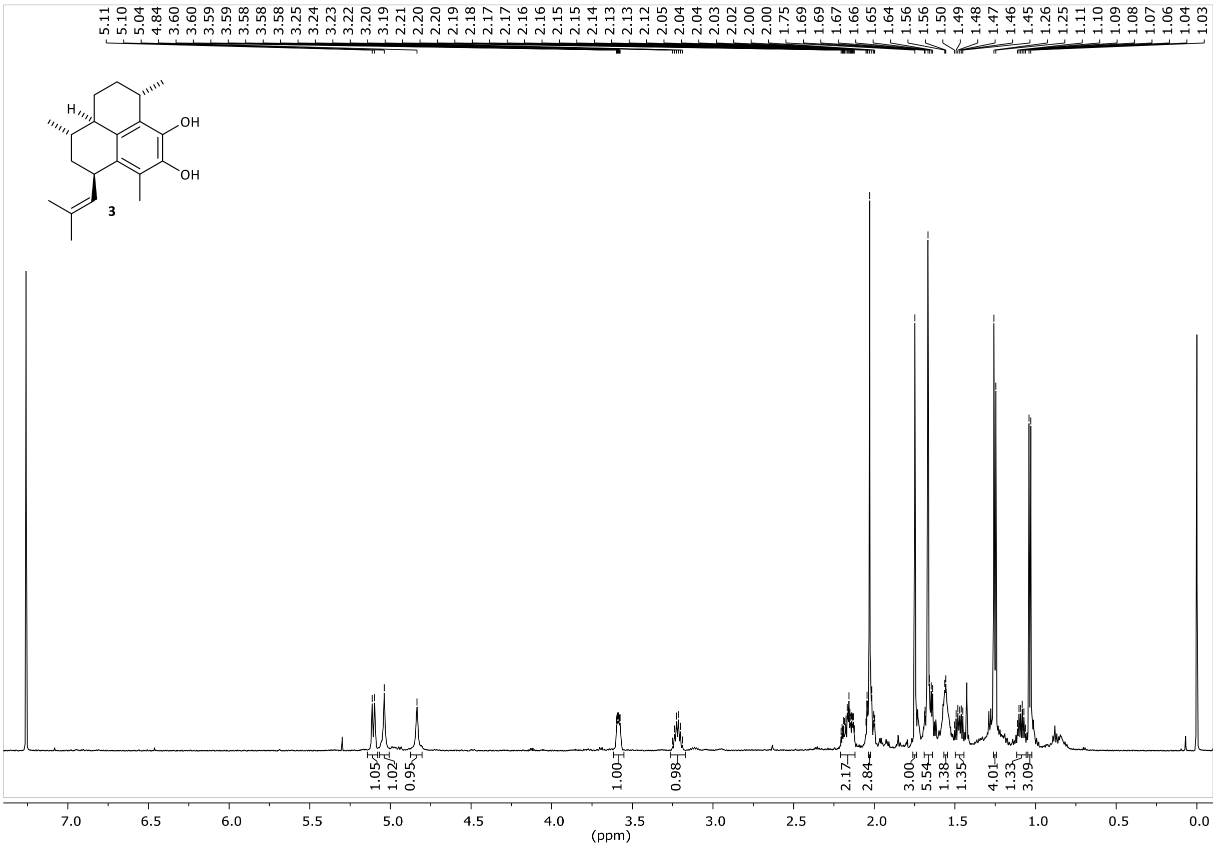


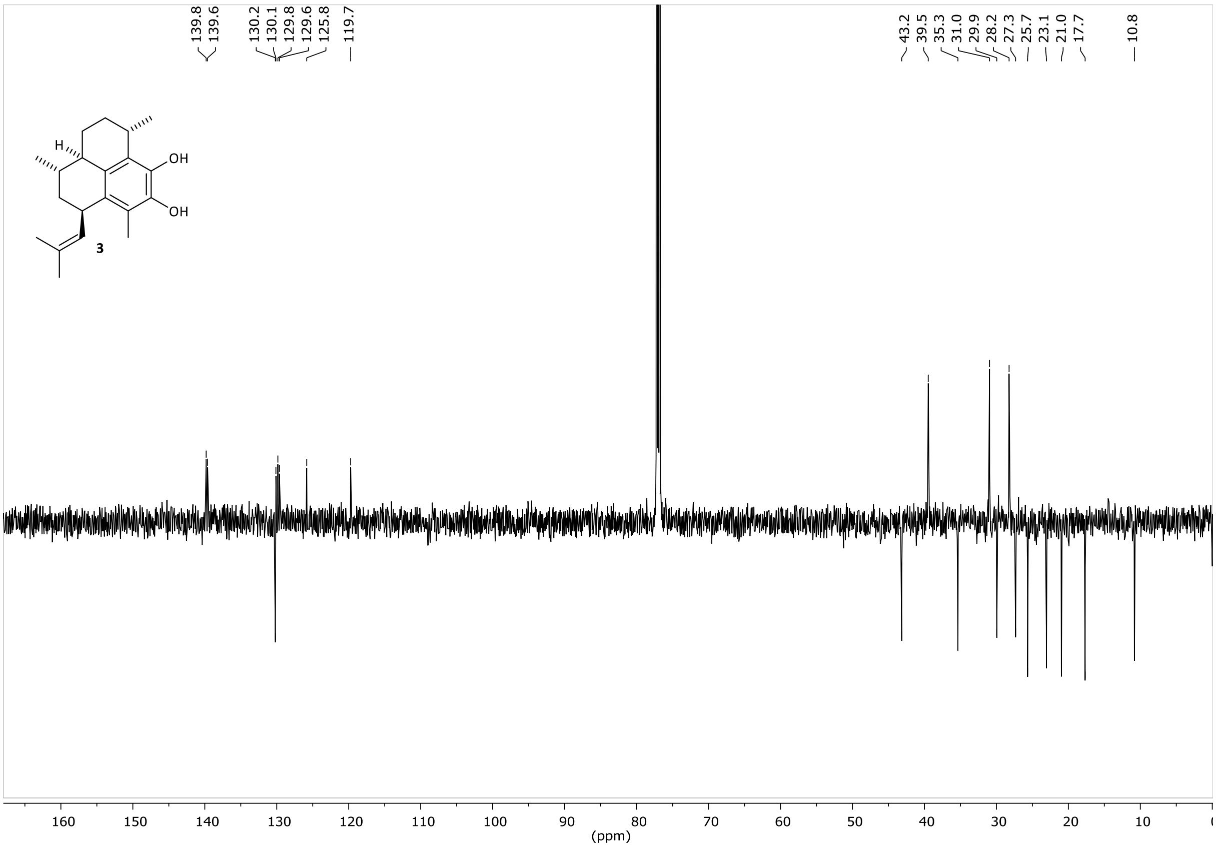

$^{1}$H and $^{13}$C NMR spectra of compound **21**

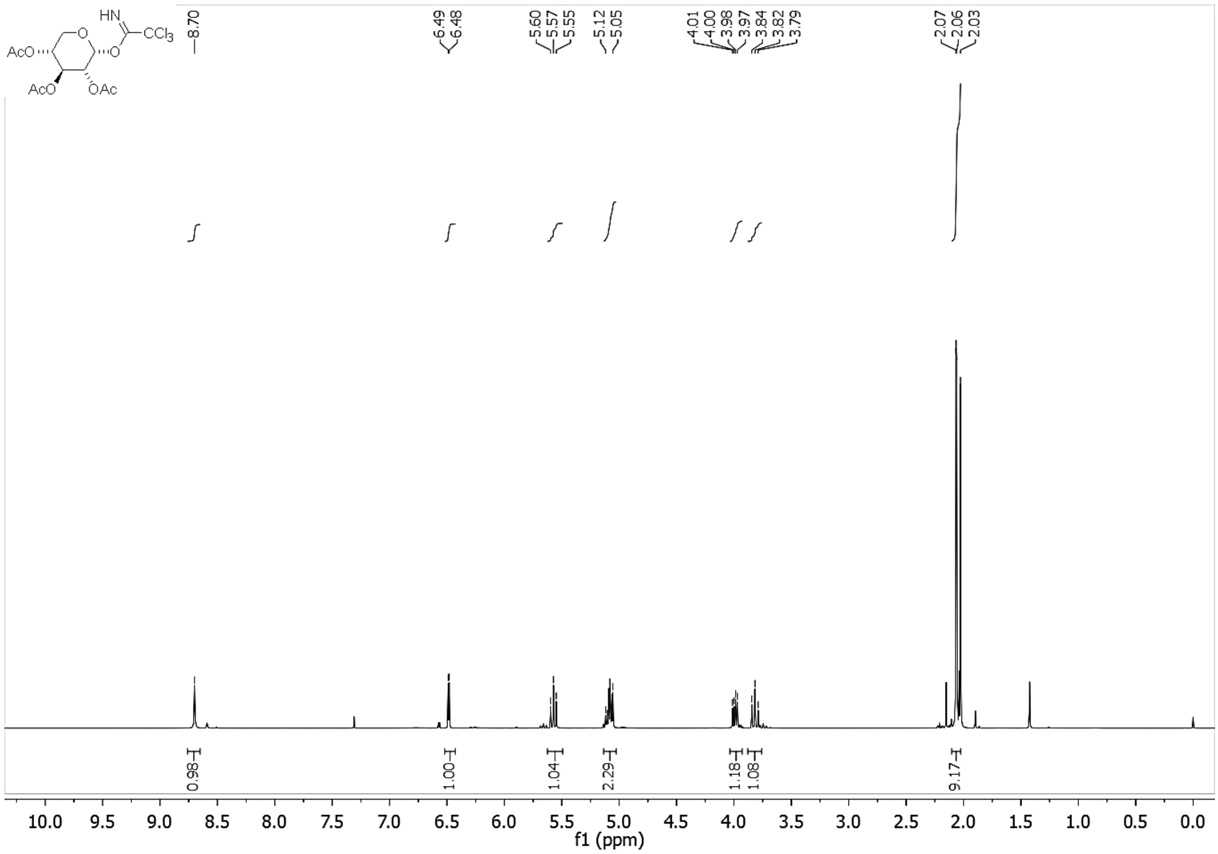


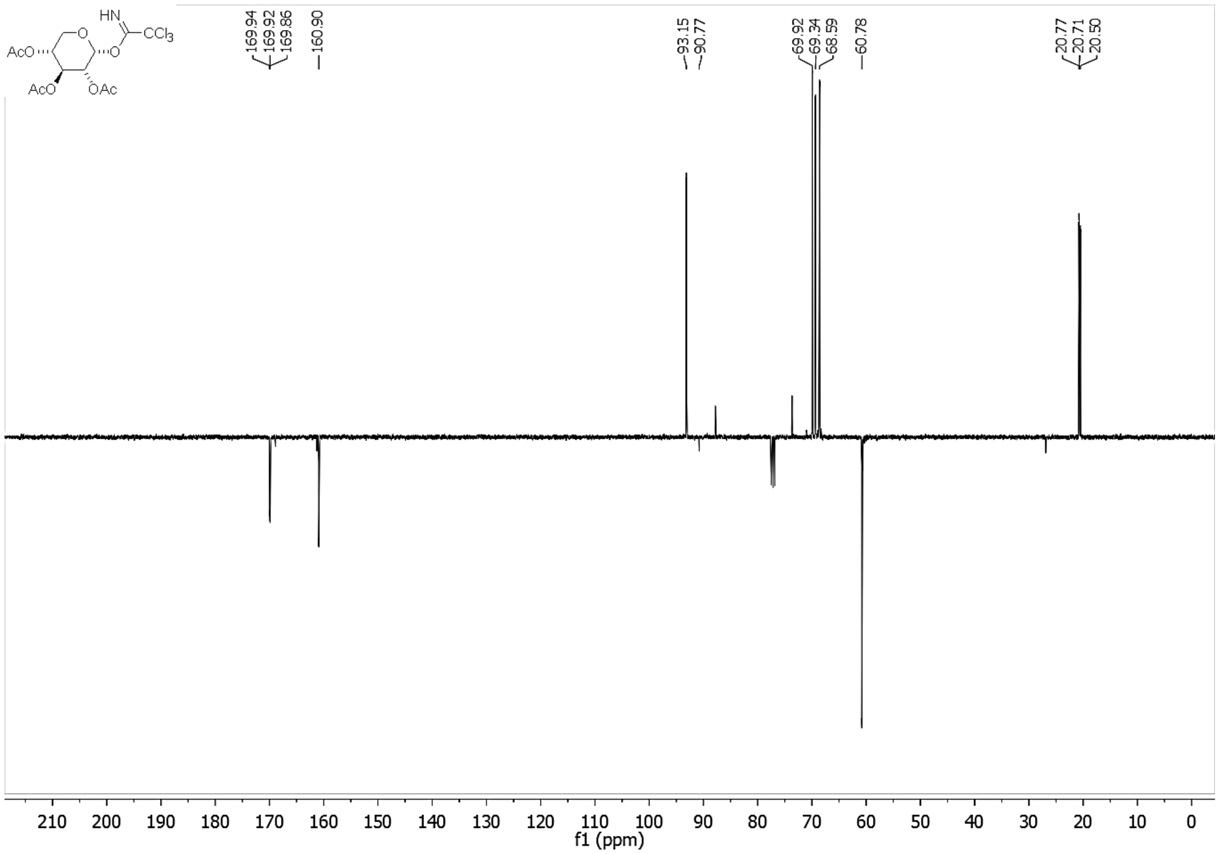

$^{1}$H and $^{13}$C NMR spectra of compound ***iso*-3**

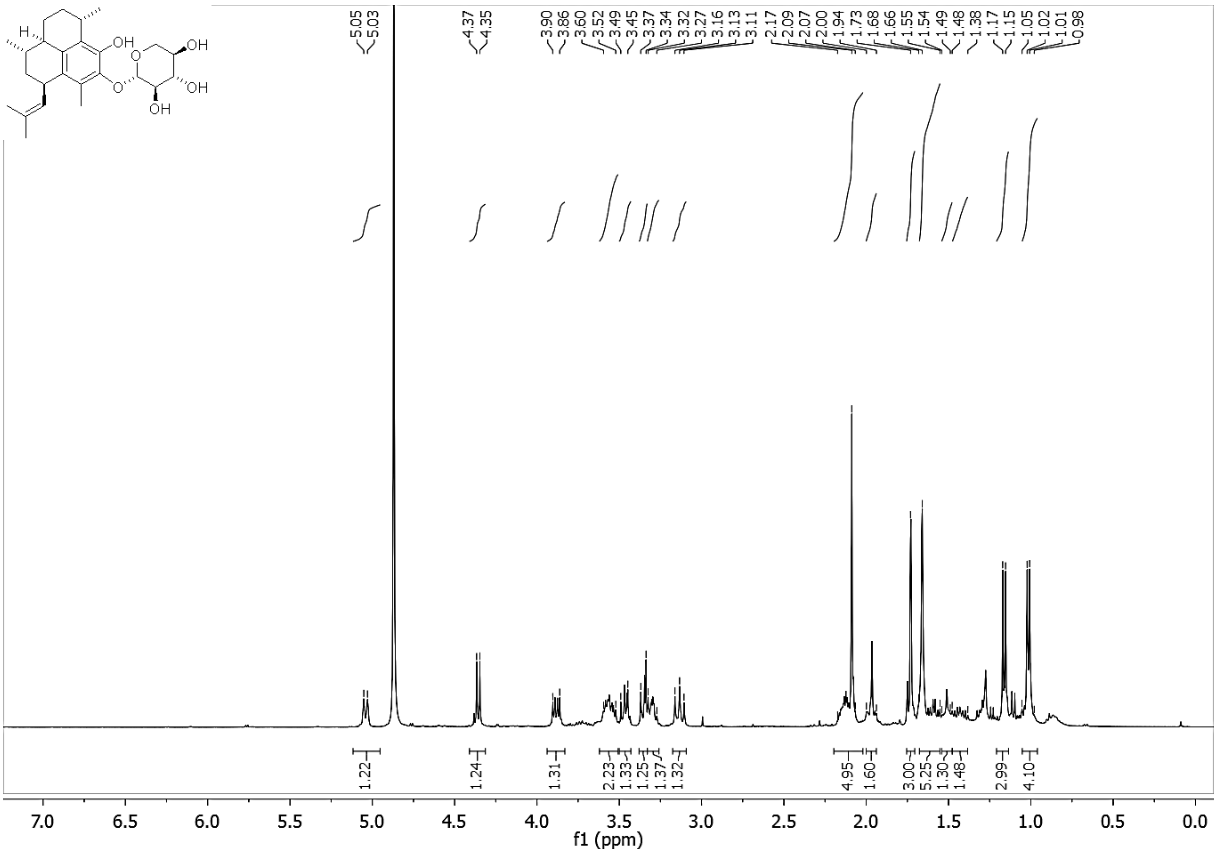


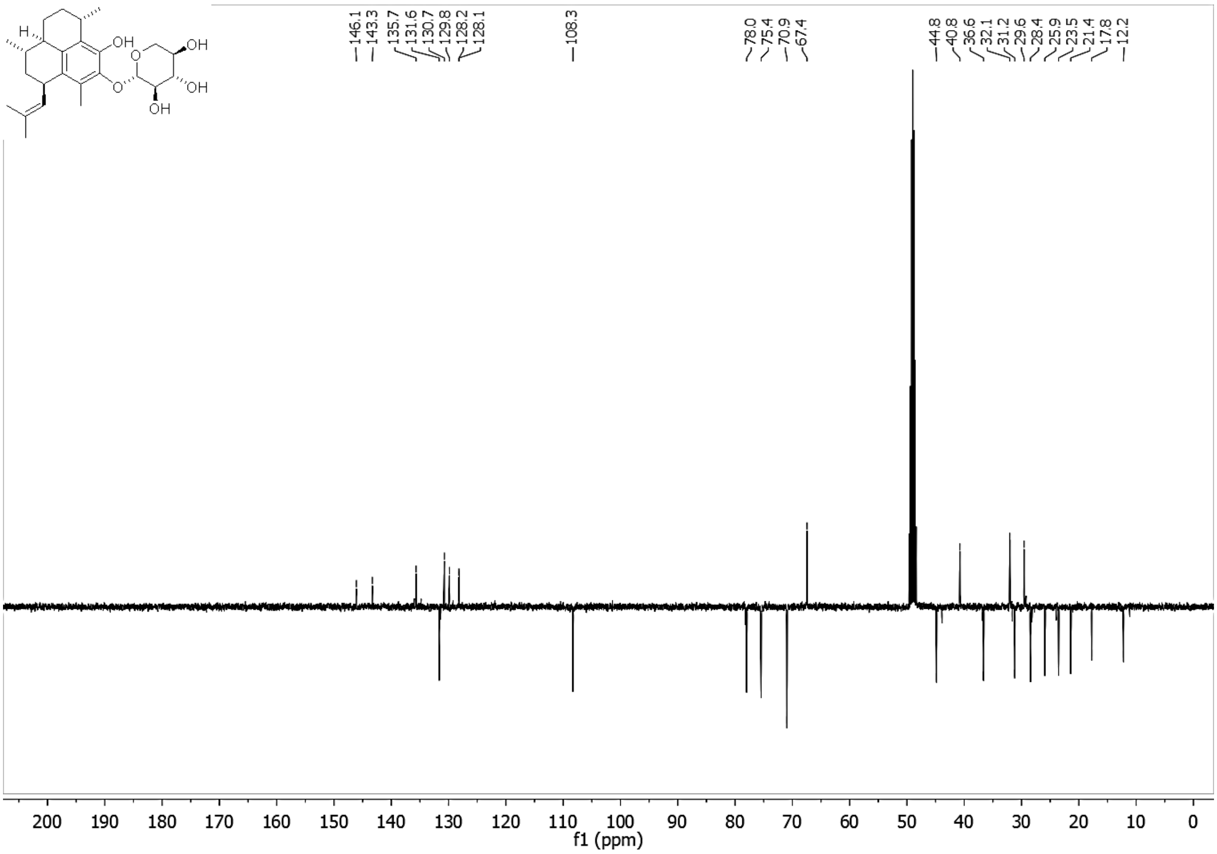

# 5. X-Ray Crystal Data

## 5.1 X-ray crystal data of compound 19

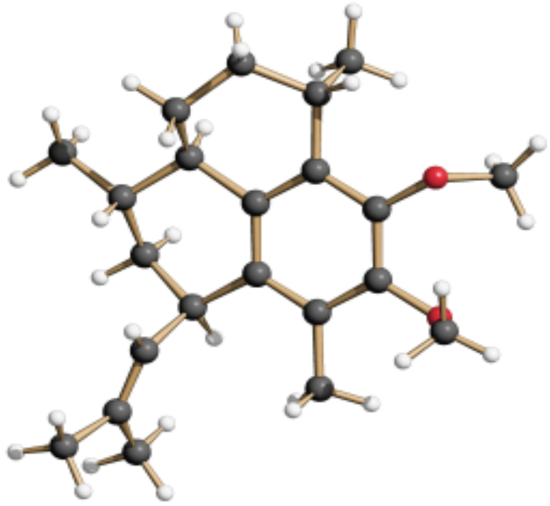

| | | |
|---|---|---|
| Identification code | susu797 | |
| Empirical formula | $C_{22}H_{32}O_2$ | |
| Formula weight | 328.47 | |
| Temperature | 100(2) K | |
| Wavelength | 1.54178 Å | |
| Crystal system | Triclinic | |
| Space group | P1 | |
| Unit cell dimensions | a = 10.019(9) Å | a= 90.09(9)° |
| | b = 10.125(8) Å | b= 100.36(10)° |
| | c = 19.95(3) Å | g = 108.11(9)° |
| Volume | 1889(4) $Å^3$ | |
| Z | 4 | |
| Density (calculated) | 1.155 $Mg/m^3$ | |
| Absorption coefficient | 0.552 $mm^{-1}$ | |
| F(000) | 720 | |
| Crystal size | 0.150 x 0.070 x 0.030 $mm^3$ | |
| Theta range for data collection | 4.515 to 72.672° | |
| Index ranges | -12<=h<=12, -12<=k<=12, -24<=l<=24 | |
| Reflections collected | 24376 | |
| Independent reflections | 12987 [R(int) = 0.0509] | |
| Completeness to theta = 67.679° | 97.4 % | |
| Absorption correction | None | |
| Refinement method | Full-matrix least-squares on $F^2$ | |
| Data / restraints / parameters | 12987 / 3 / 893 | |
| Goodness-of-fit on $F^2$ | 1.032 | |
| Final R indices [I>2sigma(I)] | R1 = 0.0904, wR2 = 0.2484 | |
| R indices (all data) | R1 = 0.0959, wR2 = 0.2561 | |
| Absolute structure parameter | 0.03(10) | |
| Extinction coefficient | n/a | |
| Largest diff. peak and hole | 0.891 and -0.398 e.$Å^{-3}$ | |

## 6. Enantiomeric analysis through chiral GC

**Figure S-6.1.** Gaschromatograms of compound **8a** using a chiral stationary phase. Top: enantio-enriched sample (≥98% *ee*); Bottom: racemic reference sample (containing small impurities resulting from hydrogenation/isomerization).

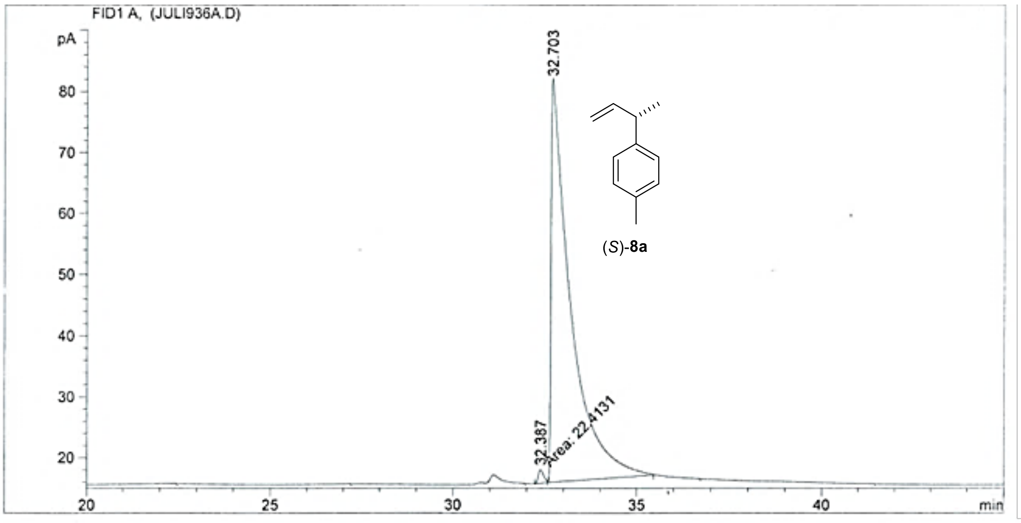


Signal 1: FID1 A,

| Peak # | RetTime [min] | Type | Width [min] | Area [pA*s] | Height [pA] | Area % |
|---|---|---|---|---|---|---|
| 1 | 32.387 | MM | 0.1653 | 22.41306 | 2.26016 | 0.91692 |
| 2 | 32.703 | VB | 0.4518 | 2421.98022 | 66.05611 | 99.08308 |
| Totals : | | | | 2444.39329 | 68.31627 | |

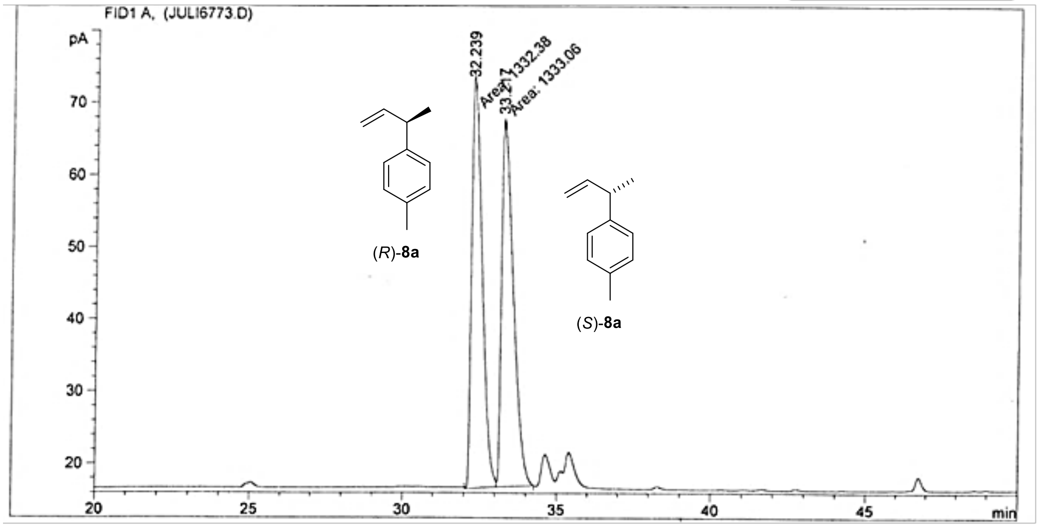


Signal 1: FID1 A,

| Peak # | RetTime [min] | Type | Width [min] | Area [pA*s] | Height [pA] | Area % |
|---|---|---|---|---|---|---|
| 1 | 32.239 | MF | 0.3859 | 1332.37927 | 57.54541 | 49.98720 |
| 2 | 33.217 | FM | 0.4351 | 1333.06177 | 51.06683 | 50.01280 |
| Totals : | | | | 2665.44104 | 108.61225 | |

**Figure S-6.2.** Gaschromatograms of compound **8b** using a chiral stationary phase. Top: enantio-enriched sample (>83% *ee*); Bottom: racemic reference sample.

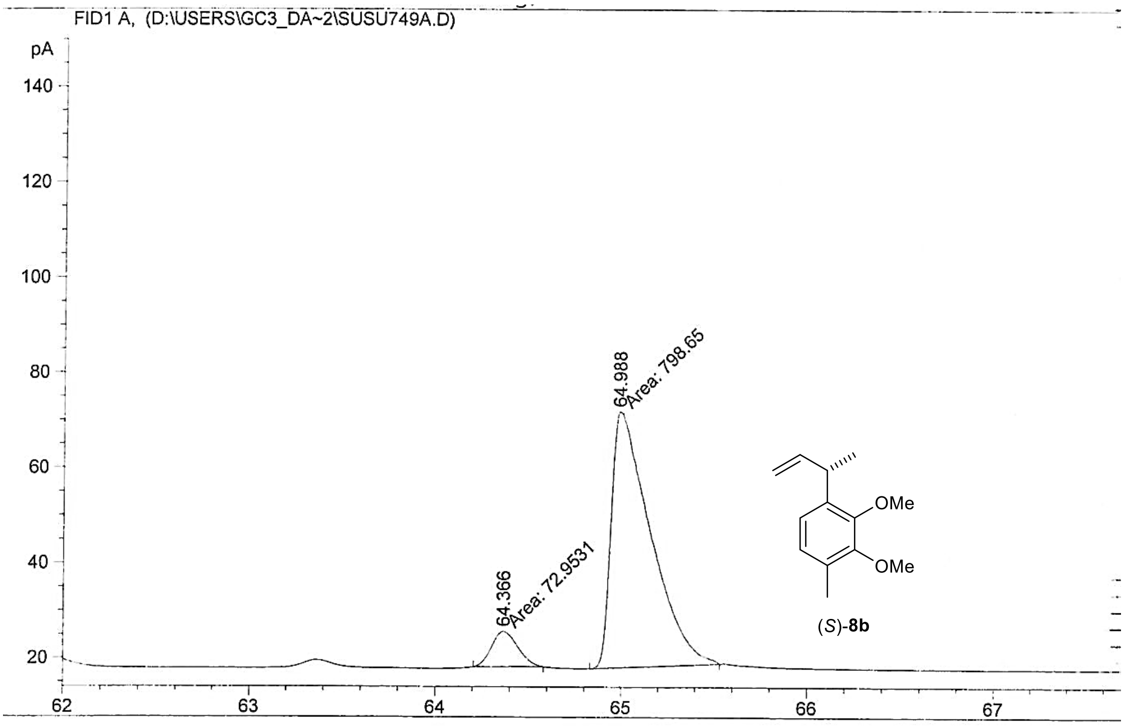


Signal 1: FID1 A,

| Peak # | RetTime [min] | Type | Width [min] | Area [pA*s] | Height [pA] | Area % |
|---|---|---|---|---|---|---|
| 1 | 64.366 | MM | 0.1633 | 72.95306 | 7.44431 | 8.36999 |
| 2 | 64.988 | MM | 0.2475 | 798.65002 | 53.77990 | 91.63001 |
| Totals : | | | | 871.60309 | 61.22421 | |

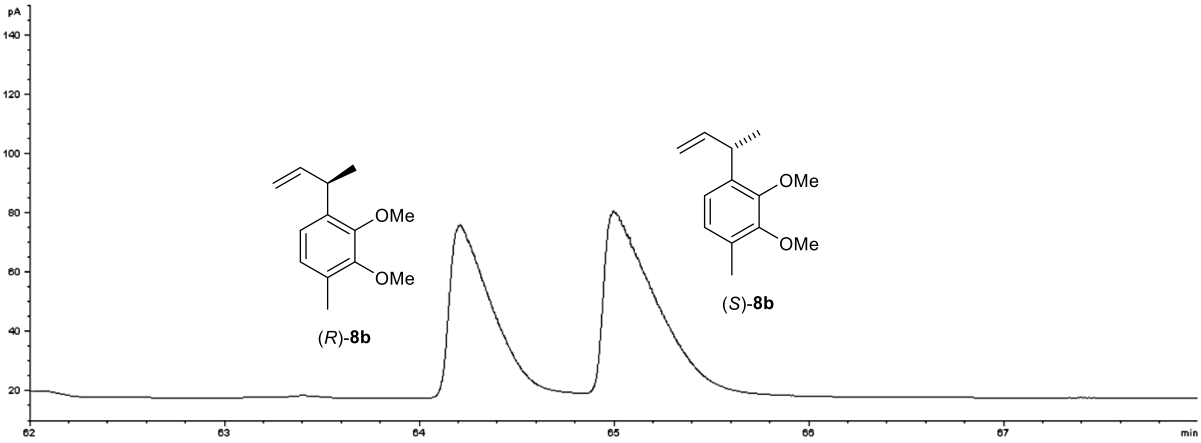

**Figure S-6.3.** Gaschromatograms of compounds *trans*-**6a**/*cis*-**6a** using a chiral stationary phase. Top: enantio-enriched sample (≥98% *ee*) of a ca. 87:13 mixture; Bottom: racemic reference sample of a 2:1 mixture.

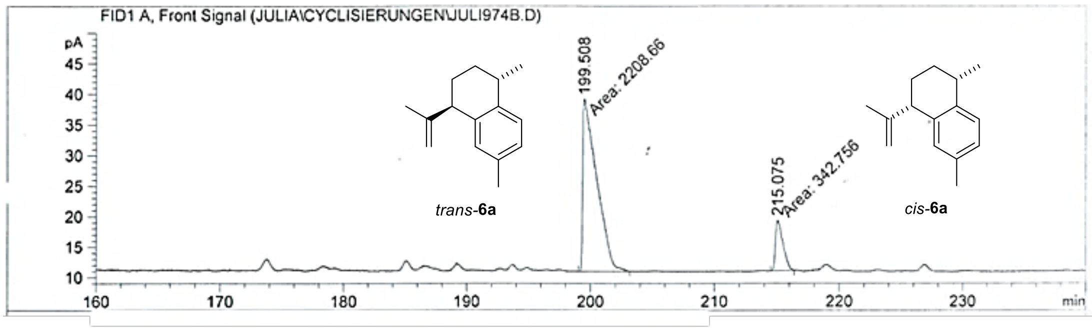


Signal 1: FID1 A, Front Signal

| Peak # | RetTime [min] | Type | Width [min] | Area [pA*s] | Height [pA] | Area % |
|---|---|---|---|---|---|---|
| 1 | 199.508 | MM | 1.3094 | 2208.66235 | 28.11250 | 86.56606 |
| 2 | 215.075 | MM | 0.6882 | 342.75589 | 8.30031 | 13.43394 |
| Totals : | | | | 2551.41824 | 36.41281 | |

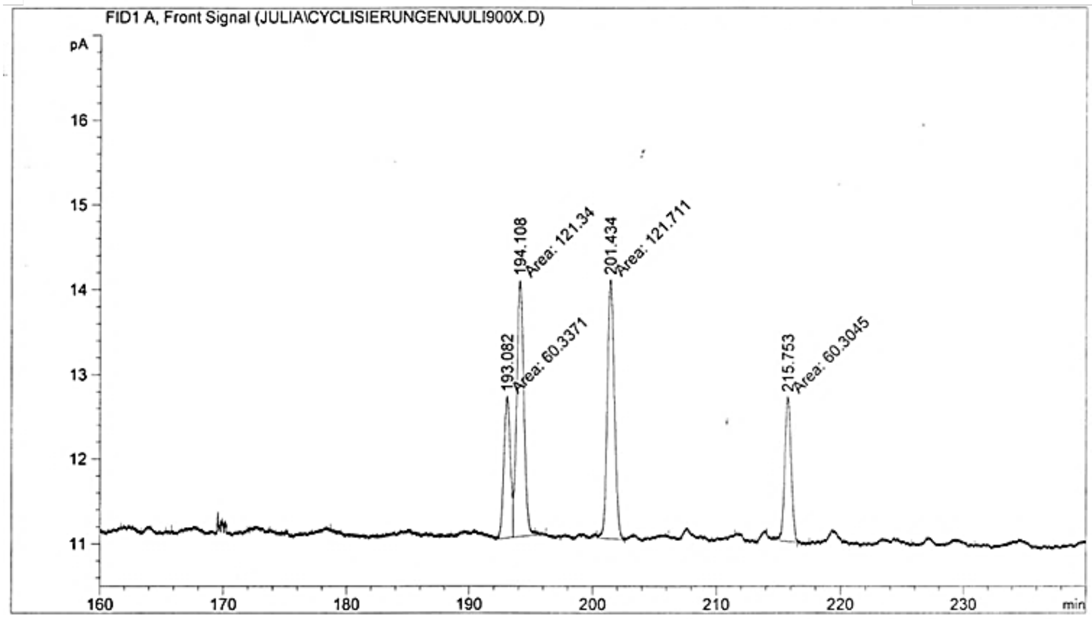


| Peak # | RetTime [min] | Type | Width [min] | Area [pA*s] | Height [pA] | Area % |
|---|---|---|---|---|---|---|
| 1 | 193.082 | MF | 0.6012 | 60.33708 | 1.67263 | 16.59014 |
| 2 | 194.108 | FM | 0.6678 | 121.34007 | 3.02815 | 33.36337 |
| 3 | 201.434 | MM | 0.6623 | 121.71083 | 3.06297 | 33.46532 |
| 4 | 215.753 | MM | 0.5867 | 60.30448 | 1.71317 | 16.58117 |
| Totals : | | | | 363.69246 | 9.47691 | |

# 7. Determination of anti-inflammatory activities

## 7.1 NFκB reporter gene assay with one hour incubation of LPS/ TNFα

MDA-MB-231 breast cancer cells were transfected with the vector pNL3.2.NF-κB-RE[NlucP/NF-κB/Hygro] from *Promega* so that the expression of a luciferase reporter gene is under control of a NF-κB CMV promoter, as previously described in an earlier publication.[1] Cells were cultivated in DMEM-medium (supplemented with 10% FCS and 1% Pen/Strep) and seeded with a density of $5x10^5$ cells/ml in 384-well plates by use of the CyBio® pipetting robot (*Analytic Jena AG*). After 24 h of incubation at 37 °C, the test compound, in six dilution stages (1:2, starting with 100 μM), was added and further 20 min incubated. Subsequently, the cells were treated with 1 μg/ml LPS or 6 ng/ml TNFα for 1 h. The luciferase activity was detected with the NanoGlo Luciferase Assay from *Promega*. All NanoGlo substrates and buffers were premixed in a 1:50 ratio and reagent was added to the wells in a 1:1 ratio. The luminescence was determined immediately (*Tecan M200 Reader*).


[1] J. Sperlich, R. Kerr, N. Teusch, *Mar. Drugs* **2017**, *15*, 262.
